# Decoder-FFPE-seq enables sensitive, genome-wide spatial transcriptomics of archival tissues at single-cell resolution

**DOI:** 10.1126/sciadv.ady5085

**Published:** 2026-10-30

**Authors:** Jiao Cao, Jun Zeng, Yiwei Meng, Shanqing Huang, Jing Zhang, Xing Fang, Yang Guo, Weixiong Shi, Yutong Liu, Zhong Zheng, Jianchen Fang, Xuepeng Xiong, Gang Chen, Lingling Wu, Chaoyong Yang

**Affiliations:** ^1^ Institute of Molecular Medicine, Shanghai Key Laboratory for Nucleic Acid Chemistry and Nanomedicine, Department of Urology, Department of pathology, Renji Hospital, Shanghai Jiao Tong University School of Medicine, Shanghai 200127, China.; ^2^ State Key Laboratory of Oral & Maxillofacial Reconstruction and Regeneration, Key Laboratory of Oral Biomedicine Ministry of Education, Hubei Key Laboratory of Stomatology, School & Hospital of Stomatology, TaiKang Center for Life and Medical Sciences, Frontier Science Center for Immunology and Metabolism, Wuhan University, Wuhan 430079, China.; ^3^ The MOE Key Laboratory of Spectrochemical Analysis & Instrumentation, State Key Laboratory of Physical Chemical of Solid Surfaces, Department of Chemical Biology, College of Chemistry and Chemical Engineering, Xiamen University, Xiamen 361005, China.; ^4^ Suzhou Dynamic Biosystems Co. Ltd., Suzhou 215200, China.

## Abstract

Formalin-fixed paraffin-embedded (FFPE) tissue–compatible spatial transcriptomics (ST) is transforming biology and histopathology, but achieving high sensitivity, genome-wide coverage, single-cell resolution, and researcher accessibility remains challenging. To address these challenges, we here present a dendrimeric DNA coordinate barcoding design for FFPE tissue–adapted spatial RNA sequencing (Decoder-FFPE-seq) by integrating RNA-to-DNA probe conversion with blotting-assisted barcoding. Decoder-FFPE-seq provides single-cell resolution with a >2.6-fold sensitivity increase in whole-transcriptome mouse brain and archived human sample profiling over existing platforms, resolving potential cellular identity of low-abundance genes, finer tissue architecture, and capturing an epithelial-mesenchymal transition–associated signature for prognostic value. With expanded probe libraries, Decoder-FFPE-seq captures long noncoding and viral RNAs, revealing their spatial coregulatory networks with messenger RNAs in oral squamous cell carcinomas (OSCCs). In an OSCC immunotherapy cohort, two CD8^+^ T cell subpopulations were respectively identified in pre- and postchemoimmunotherapy samples, potentially linked to treatment response. Decoder-FFPE-seq establishes a robust, high-resolution, and accessible framework for sensitive ST profiling of archival tissues in fundamental and translational studies.

## INTRODUCTION

Spatial transcriptomics (ST) uncovers cellular architecture and molecular networks within native tissue context, advancing insights into biology, organogenesis, and disease pathology (*1*–*5*). In oncology, ST provides unprecedented resolution to deconstruct the tumor microenvironment (TME), revealing its cellular composition, spatial organization, and interactions, unraveling the underlying molecular mechanisms in tumor initiation, progression, and therapy resistance (*6*, *7*). Translating these insights into clinical practice requires ST platforms compatible with formalin-fixed paraffin-embedded (FFPE) samples, the foundation of pathology archives (*8*). These vast repositories offer unparalleled opportunities to link spatial molecular landscapes with annotated clinical outcomes, thereby driving precision oncology. However, RNAs within FFPE samples undergo cross-linking, fragmentation, and degradation during fixation and long-term storage. These alterations induce polyadenylate [poly(A)] tail loss and adduct formation between nucleic acids and biomolecules (*9*), undermining poly(T)-based RNA capture and enzymatic reactions (*10*), hampering the ST application to archival specimens.

Effective ST profiling in FFPE samples requires four key capabilities: (i) specific recovery of genome-wide transcripts, spanning mRNAs, regulatory RNAs, and even pathogenic RNAs, while excluding interference from genomic DNA and highly abundant (∼80%) but information-limited ribosomal RNAs (rRNAs) (*11*, *12*), to reconstruct accurate spatial gene expression and coregulatory networks; (ii) sensitive detection of low-abundance or severely degraded transcripts in archived tissues for the identification of biomarkers and rare cell types, thereby facilitating a deeper understanding of physiological and pathological mechanisms; (iii) single-cell resolution for resolving structural details and cell-cell interactions; and (iv) accessibility, adoptability, and reproducibility for widespread use across research communities and feasibility for large-scale longitudinal cohort studies. Despite several pioneer explorations, options for FFPE-compatible ST methods that satisfy these requirements are limited.

Imaging-based ST methods such as multiplexed fluorescence in situ hybridization (FISH)/sequencing platforms [e.g., 10x Genomics’ Xenium (*13*), NanoString’s CosMx (*14*, *15*), and Vizgen’s MERSCOPE (*16*)] achieve subcellular resolution and high sensitivity in FFPE tissues (*17*), but their limited throughput, labor-intensive workflows, and imaging signal drift preclude transcriptome-wide scalability. Array-based ST platforms, including commercial systems such as 10x Genomics’ Visium CytAssist_FFPE (*13*) and Visium HD (*18*), as well as emerging methods like Stereo-seq V2 (*19*) and Patho-DBiT (*20*), bypass the throughput ceiling of imaging systems by enabling whole-transcriptome in situ capture and spatial barcoding. Nevertheless, each retains intrinsic constraints: Visium offers only mRNA analysis, exhibits modest sensitivity and locks users into closed-source reagent kits. Stereo-seq V2 and Patho-DBiT, while covering total RNAs, suffer from promiscuous capture of genomic DNAs and rRNAs using random primers or in situ polyadenylation. This compromises their sensitivity and specificity, particularly for detection of low-abundance and severely degraded transcripts. In addition, Patho-DBiT is hindered by tissue deformation and cross-contamination risks across microchannels, while Stereo-seq V2 requires complex position decoding, both of which restrict accessibility.

We previously developed Decoder-seq (*21*), using dendrimeric DNA slides (Decoder slides) containing high-density deterministic spatial barcodes for highly sensitive ST analysis in fresh-frozen (FF) tissues. Here, we report FFPE tissue–adapted Decoder-seq (Decoder-FFPE-seq), which converts RNA fragments into encodable and amplifiable DNA probes and leverages blotting-assisted spatial barcoding on high–barcode-density Decoder slides. Decoder-FFPE-seq incorporates several key advances, achieving sensitive, high-resolution, whole-transcriptome profiling of archival tissues in a widely accessible manner. First, we broadened ST scope to include wider RNA species for a deep understanding of spatial biology. Multiple split-probe libraries were designed for specific and unbiased recovery of whole mRNAs, key regulatory long noncoding RNAs (lncRNAs), and pathogenic RNAs, effectively minimizing nonspecific capture of DNAs and rRNAs. Second, we implemented blotting-assisted spatial barcoding on Decoder slides to markedly improve gene detection sensitivity. The blotting-assisted strategy separates tissue pretreatment and probe labeling on independent glass slides, thereby preventing barcoded DNA loss and potential contamination and fully leveraging the high barcoding capacity of Decoder slides. Third, we further increased the spatial resolution of Decoder slides, enabling Decoder-FFPE-seq to achieve single-cell resolution (8-μm spots) for high-definition mapping of transcriptional and cellular landscapes. Fourth, we established an easy-to-follow workflow with a detailed protocol, spanning tissue pretreatment, probe targeting, and blotting-assisted spatial barcoding to maximize RNA recovery while ensuring reproducibility and accessibility. Together with the openly available fabrication process of Decoder slides (*21*), Decoder-FFPE-seq ensured broad accessibility and adoptability across research communities.

With these innovations, Decoder-FFPE-seq achieved a >2.6-fold sensitivity increase at single-cell resolution in mouse brain and an archived oral squamous cell carcinoma (OSCC) sample compared to existing ST platforms (e.g., 10-μm bin Stereo-seq V2 or 8-μm bin Visium HD). We effectively detected low-abundance nonsensory G protein–coupled receptors (GPCRs), revealed biologically meaningful colocalization patterns that potentially defined the cell identity of *Gpr83*-expressing spots, and resolved sparse inhibitory neuron subtypes within fine-grained mouse tissue structures. Our approach demonstrated robust performance in spatially profiling transcriptomes of challenging human tissues preserved up to 6 to 20 years, even in a 20-year-old OSCC with a distribution value at 200 nucleotides (nt) (DV200) of only 17%. In an OSCC sample with stromal-invasive tumor, 8-μm–spot Decoder-FFPE-seq reliably resolved the dysplasia-stroma interface and a tumor subcluster, identifying an epithelial-mesenchymal transition (EMT)–associated signature to provide prognostic value across patients with head and neck squamous cell carcinoma (HNSC), cervical squamous cell carcinoma (CESC), and esophageal squamous cell carcinoma (ESCA). By spatial coprofiling of mRNA-lncRNA and host mRNA-pathogenic RNA, Decoder-FFPE-seq uncovered their interaction networks that regulated tumor progression. Application to an immune checkpoint blockade (ICB) trial of OSCC samples revealed spatial TME heterogeneity across pathology response groups and tracked spatiotemporal dynamics of key cellular and molecular compartments pre- and postchemoimmunotherapy. Collectively, Decoder-FFPE-seq provides a powerful and accessible platform for single-cell resolution, genome-wide ST analysis in archival tissues, opening promising avenues for both fundamental biology and translational research.

## RESULTS

### Workflow of Decoder-FFPE-seq

Decoder-FFPE-seq consists of three main steps: (a) RNA-to-DNA probe conversion, (b) blotting-assisted spatial barcoding, and (c) sequencing and bioinformatic analysis (Fig. 1A). To recover cross-linked, fragmented, and degraded mRNAs, lncRNAs, and viral RNAs from FFPE tissues, we designed split-probe panels that hybridize each transcript at one or more adjacent sites. Each left probe carries a polymerase chain reaction (PCR) handle (Read 2) and a 25-nt targeting sequence, while the right probe contains a 5-phosphorylated 25-nt targeting sequence and a poly(A) tail (Fig. 1A-a and fig. S1A). Only correctly paired probes hybridizing adjacently are ligated into an encodable [3′ poly(A) tail] and amplifiable (5′ Read 2) sequence. This split-probe design eliminates nonspecific binding of single probes, ensuring highly specific and accurate RNA detection in Decoder-FFPE-seq.

**Fig. 1.**
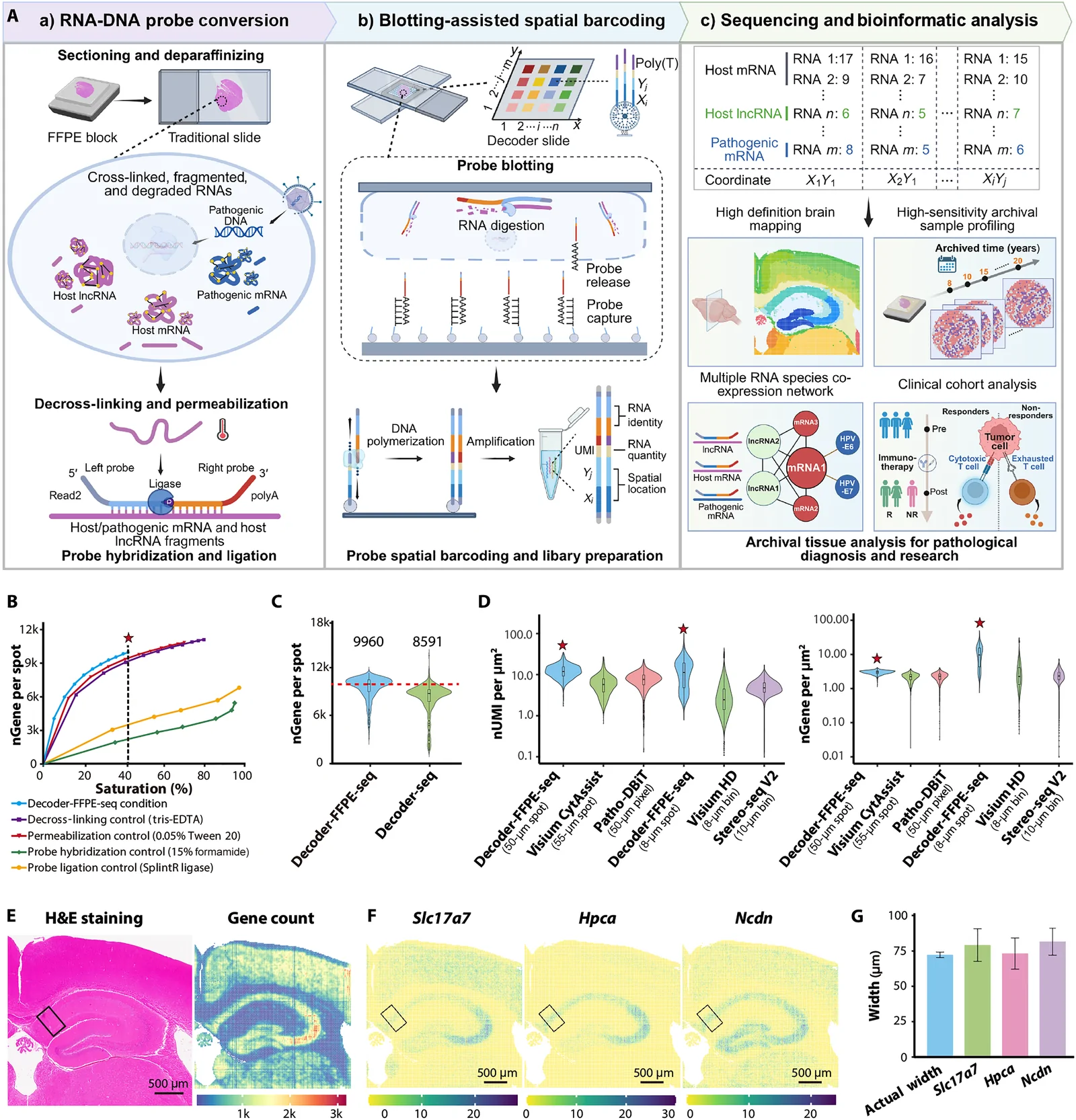
Workflow and performance of Decoder-FFPE-seq in mouse tissues. (**A**) Schematic workflow. (a) RNA-to-DNA probe conversion. FFPE sections are processed by deparaffinization, de–cross-linking, and permeabilization, followed by probe hybridization and ligation. (b) Blotting-assisted spatial barcoding. Tissue is aligned with barcoded DNA array. Ligated probes are released, captured, and extended with unique molecular identifiers (UMIs), spatial barcodes, and PCR handles for next-generation sequencing (NGS). (c) Sequencing and bioinformatics analysis. Probe sequences, UMIs, and spatial barcodes are extracted to decode RNA identity, abundance, and location for applications of mouse models, clinically archival samples, and ICB-treated clinical cohorts. Created in BioRender. J.Ze. (2026); https://BioRender.com/x817ooc. (**B**) Median number of genes (nGene) per spot across conditions tested for de–cross-linking, permeabilization, probe hybridization, and ligation in mouse olfactory bulbs (MOBs). (**C**) Comparisons of the median nGene detected in the Decoder-FFPE-seq and Decoder-seq datasets. (**D**) Distribution of number of UMIs (nUMI; left) and nGene (right) per square micrometer of FFPE mouse brains from Decoder-FFPE-seq versus other FFPE-compatible ST platforms (Visium CytAssist_FFPE, Patho-DBiT, Stereo-seq V2, and Visium HD). In Patho-DBiT, only mRNA counts were used. Red stars denote Decoder-FFPE-seq’s performance. (**E**) Hematoxylin and eosin (H&E) staining image (left) and gene (right) count maps of the mouse brain from 8-μm–spot Decoder-FFPE-seq. (**F**) Expression of regional marker genes in the mouse hippocampus. Black boxes indicate regions selected for diffusion analysis. (**G**) Comparison of actual cornu ammonis 1 (CA1) width from fig. S8D versus full width at half maximum (FWHM) of marker gene expression from (F) (*n* = 6; details in Materials and Methods).

We developed a blotting-assisted strategy to transfer ligated probes onto Decoder slides for spatial barcoding (Materials and Methods, Fig. 1A-b, and fig. S1B). By manual probe-blotting or using a custom-built alignment instrument (Materials and Methods and fig. S2), the tissue section is precisely matched with the Decoder slide’s barcode array to trigger reactions of RNA digestion and tissue removal. Then, ligated probes are released, captured by the barcoded DNAs, and enzymatically extended to incorporate unique molecular identifiers (UMIs), spatial barcodes, and Read 1 for amplification, library preparation, and sequencing (fig. S1, C and D). This blotting strategy attaches tissues to traditional slides rather than Decoder slides, which avoids barcoded DNA loss from high-temperature treatment, such as tissue baking and de–cross-linking, and prevents poly(A) tailing-probe blocking (fig. S3). Meanwhile, selective probe blotting precludes DNA and rRNA contamination during spatial barcoding. These cocontribute to enhanced gene detection sensitivity. With next-generation sequencing (NGS) and bioinformatics analysis, RNAs are remapped to their original spatial coordinates, achieving mouse brain architecture reconstruction, TME profiling in long-term archival samples, spatial RNA coregulatory network discovery in OSCCs, and spatial cellular dynamics tracking in clinical cohorts undergoing ICB therapy (Fig. 1A-c).

To optimize transcript recovery from FFPE tissues, we systematically evaluated de–cross-linking, permeabilization, probe hybridization, and ligation conditions using mouse olfactory bulbs (MOBs) as a model (Materials and Methods). The optimized protocol, combined with a 21,178 split-probe panel covering the entire mouse protein-coding transcriptome (table S1), detected ∼19,297 unique genes with a median of 60,466 UMIs and 9960 genes per 50-μm spot at ∼40% sequencing saturation (Fig. 1B and fig. S4A). Decoder-FFPE-seq exhibited a substantial improvement of gene detection sensitivity compared to those under control treatment conditions and even outperformed our published Decoder-seq data on FF MOBs (Fig. 1C). Moreover, the protein-coding gene counts exhibited a high concordance between the Decoder-FFPE-seq and Decoder-seq datasets (*R* = 0.88; fig. S4, B and C), accompanied by consistent spatial distributions of layer-specific marker genes (fig. S4D). Unsupervised clustering unveiled clusters whose spatial distribution faithfully recapitulated the layered MOB structure (fig. S4E). These results confirmed that Decoder-FFPE-seq achieves sensitive and accurate spatial profiling of FFPE tissues, comparable to FF samples. To enhance accessibility, adoptability, and reproducibility for the broader research community, we provide a step-by-step protocol in the Supplementary Materials, ranging from tissue pretreatment, probe targeting, blotting-assisted spatial barcoding (both manual and instrument-based), library construction, and sequencing.

### Evaluation and benchmarking of Decoder-FFPE-seq

We benchmarked the performance of 50-μm–spot Decoder-FFPE-seq in mouse brain profiling against other FFPE-compatible ST platforms of similar spatial resolution, including 55-μm–spot Visium CytAssist_FFPE and 50-μm-pixel Patho-DBiT (Fig. 1D). Decoder-FFPE-seq (50-μm–spot) detected a median of 29,907 UMIs and 7571 genes per spot in the mouse brain, outperforming both Visium CytAssist_FFPE and Patho-DBiT (fig. S5, A to C). After normalizing UMI and gene counts across sequencing depths, Decoder-FFPE-seq still showed superior sensitivity (fig. S5D). Reproducibility between replicates was high (*R* = 0.99; fig. S5E), and region-specific marker genes were robustly spatially mapped, faithfully reflecting the corresponding anatomical domains (fig. S5, F and G).

To enable high-definition spatial mapping, we advanced Decoder-FFPE-seq to single-cell resolution. We fabricated 8-μm–spot Decoder slides containing 36,864 barcoded spots by narrowing the barcoding channel width from 50 to 8 μm and scaling the channel number from 50 to 192 (fig. S6). This width reduction substantially increased flow resistance and fabrication challenge. We thus optimized the microfluidic chip design by incorporating rounded corners and variable channel widths to reduce flow resistance (fig. S6, A to D). Fluorescence-based quality control demonstrated robust fabrication reproducibility and yield (fig. S6, E to H), which together with the cost-effective nature of this barcoding strategy (table S2; consumable costs), supported scalability of 8-μm–spot Decoder slides for routine ST applications. Despite the reduction in spot size, 8-μm–spot Decoder-FFPE-seq maintained superior detection sensitivity, yielding a median of 722 UMIs and 617 genes per 8-μm spot (Fig. 1, D and E). This markedly outperformed comparable high-resolution platforms, such as Stereo-seq V2 (10-μm bin: 429 UMIs and 232 genes) and Visium HD (8-μm bin: 156 UMIs and 145 genes). To account for platform-specific variations in spatial resolution, array architecture, and barcoded-active area fraction, we systematically compared these features and further normalized UMI and gene counts to per unit barcoded area (in square micrometer) for fair sensitivity comparison (Fig. 1D and table S3). Decoder-FFPE-seq achieved a 2.63- to 4.63-fold higher gene detection sensitivity than the two methods at matched sequencing reads (Fig. 1D and fig. S7, A and B), underscoring its potential to detect low-abundance transcripts. While current effective barcoded capture area represented 25% of the total barcoded regions due to large interchannel spacing, limiting the captured information from tissues, the fraction of barcoded-active area can be improved through optimizing microfluidic chip design with minimized channel spacing. Last, replicate datasets exhibited high concordance (*R* = 0.98; fig. S7C), confirming the platform’s robustness.

Spatial accuracy of RNA detection, another key quality parameter for high-resolution ST, was evaluated at 8-μm resolution. Focusing on the hippocampal cornu ammonis 1 (CA1) region, which contains a densely packed pyramidal cell layer, we assessed lateral mRNA diffusion by calculating the full width at half maximum (FWHM) of the expression plots of three region-enriched genes (*Slc17a7*, *Hpca*, and *Ncdn*) (Fig. 1F, fig. S8, A to C, and Materials and Methods). Their FWHM values closely matched the actual width of corresponding CA1 regions observed in hematoxylin and eosin (H&E) staining (Fig. 1G and fig. S8D), confirming the well-controlled diffusion of Decoder-FFPE-seq for accurate spatial gene mapping. Compared with other ST platforms, the FWHM values of Decoder-FFPE-seq were much smaller than those of Stereo-seq V2 but larger than those of Visium HD and Slide-seq V2 (fig. S8, A to C). These differences likely arise from multiple factors such as tissue-dependent effects and permeabilization conditions (*22*). For example, the CA1 region in the tissue of Visium HD data was found to be narrower than that in our dataset, which may contribute to a smaller FWHM (fig. S8, D to F). To further minimize lateral RNA diffusion and enhance spatial precision, future iterations of this protocol could optimize permeabilization and incorporate electrophoresis-based probe blotting. Collectively, these data demonstrate the high sensitivity and spatial accuracy of Decoder-FFPE-seq for ST profiling.

### Decoder-FFPE-seq profiles the mouse brain with high sensitivity and accuracy at single-cell resolution

We next applied 8-μm–spot Decoder-FFPE-seq to profile low-abundance genes, fine structural details, and sparse neuronal subtypes in the mouse brain. Nonsensory GPCRs (∼300 to 400 genes), which are essential for brain development, neural signaling, immunity, and metabolism, are difficult to be profiled due to low expression levels (*23*). Decoder-FFPE-seq detected 340 nonsensory GPCRs (table S4), with UMI counts per square micrometer markedly higher than Visium HD and Stereo-seq V2 (Fig. 2A and tables S5 and S6). The expression profiles of nonsensory GPCR detected by our platform showed a stronger correlation with high-sensitivity MERFISH data (*R* = 0.90) compared to the other two methods (Fig. 2B and fig. S9A), and Gene Ontology (GO) analysis showed expected enrichment of biological processes (fig. S9B). Spatial distributions of representative genes, including *Gpr83* and *Gpr6,* closely mirrored MERFISH patterns (Fig. 2C and fig. S9C). Notably, *Gpr83* is recognized for its region-specific expression and pivotal roles in the mouse brain, but the cellular identity expressing this gene remains unclear (*24*). We observed *Gpr83* enrichment in cortical and hippocampal regions, consistent with MERFISH data and previous reports (Fig. 1C) (*24*, *25*). We compared *Gpr83*-positive (*Gpr83*^+^) and *Gpr83*-negative (*Gpr83*^−^) spots to characterize *Gpr83*-expressing cells using differential expression and GO enrichment analyses. *Gpr83*^+^ spots showed up-regulation of *Neurod6*, *Adra1d*, and *Pvalb* and down-regulation of *Mbp*, *Mobp*, and *Apoe*, indicating neuronal and inhibitory interneuron features with reduced glial signatures (fig. S9D). Enriched pathways further highlighted active neuronal and GABA-releasing (GABAergic) synaptic processes (fig. S9E). Spatial colocalization analysis further demonstrated that *Gpr83* colocalized with inhibitory interneuron markers, such as *Gad1*, *Gad2*, *Reln*, and *Pvalb*, supporting its board association with GABAergic interneurons (Fig. 1D and fig. S9F). In contrast, this colocalization was not detectable in Stereo-seq V2 and Visium HD, likely due to their limited sensitivity in detecting low-abundance transcripts (fig. S9F). Although MERFISH offers high sensitivity, its limited target panel design also constrained identification of such colocalization patterns.

**Fig. 2.**
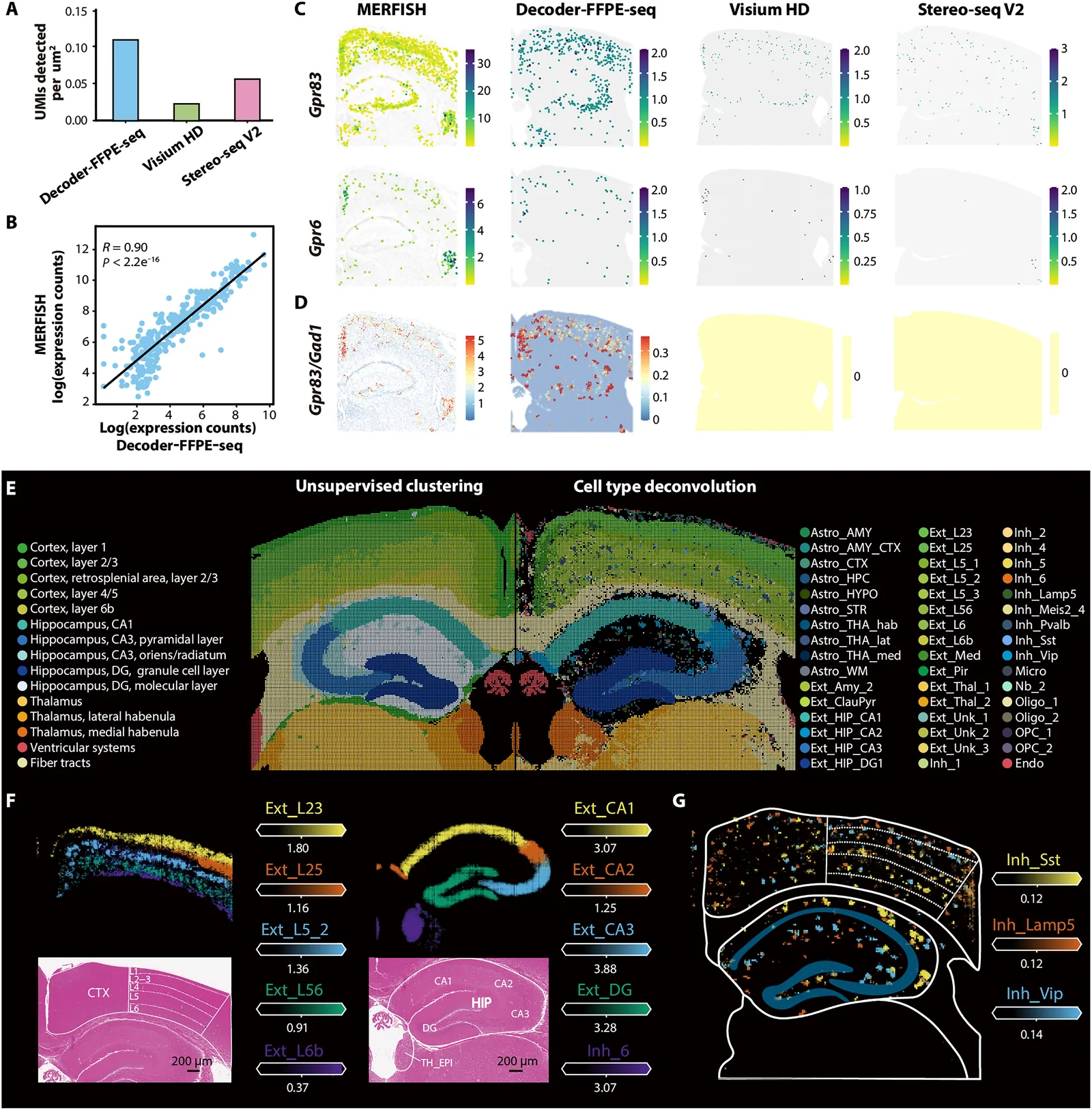
Decoder-FFPE-seq profiles the mouse brain with high sensitivity and accuracy at single-cell resolution. (**A**) Nonsensory GPCR UMIs detected per square micrometer from 8-μm–spot Decoder-FFPE-seq, Visium HD, and Stereo-seq V2. (**B**) Correlation of GPCR expression between 8-μm–spot Decoder-FFPE-seq and MERFISH data. (**C**) Spatial distribution of representative GPCR genes (*Gpr83* and *Gpr6*) across ST platforms. (**D**) Spatial colocalization of *Gpr83* and *Gad1* across ST platforms. The scale bar represents the level of colocalization between the two genes. (**E**) Unsupervised clustering of spots (left) and spatial visualization of deconvolved cell types (right) from the mouse hemibrain section in Decoder-FFPE-seq. (**F**) Fine-grained cortical layers and hippocampal subdivisions. Brain structures were manually annotated on the H&E image, and the magnified panel is a zoomed-in region from Fig. 1E. (**G**) Spatial distribution of sparse inhibitory neurons.

Using a single-nucleus RNA sequencing (snRNA-seq) reference for cell-type deconvolution (*26*), 82.5% of spots were assigned to a single cell type (fig. S10A), confirming the single-cell resolution of Decoder-FFPE-seq. We identified 50 cell types, and the spatial distribution of cell type-deconvoluted spots closely corresponded with spatial clusters, revealing fine structural details of the brain that recapitulate its anatomical morphology (Fig. 2E). For example, spatial mapping reconstructed canonical brain architectures, including six cortical layers and hippocampal subregions [CA1, CA2, CA3, dentate gyrus (DG), and the habenula; Fig. 2F]. Subregional subtype distributions matched the spatial expression of marker genes (fig. S10, B and C). Within fine-grained tissue structures, rare inhibitory neuron subtypes were also effectively resolved (Fig. 2G). Lamp5 and Vip interneurons were enriched in the superficial cortex and CA stratum radiatum, while Sst interneurons localized to deeper cortical layers and CA stratum oriens (Fig. 2G), consistent with classical marker expression and prior studies (fig. S10D) (*27*). Replicate datasets confirmed reproducibility in deconvolution results (*R* = 0.84; fig. S10E). Moreover, we compared Decoder-FFPE-seq, Stereo-seq V2, Visium HD, and snRNA-seq by evaluating marker gene expression across the three subtypes. Decoder-FFPE-seq demonstrated higher quantitative marker gene expression and showed the closest agreement with snRNA-seq in marker gene–positive cell proportions, outperforming Stereo-seq V2 and Visium HD (fig. S11). Collectively, these results highlight the high sensitivity and transcriptome-wide coverage of Decoder-FFPE-seq, suggesting its potential for resolving the cellular identity of low-abundance transcripts, fine structural details, and rare subtypes.

### Decoder-FFPE-seq characterizes TME heterogeneity associated with clinical outcomes in archived clinical tissues

To evaluate clinical applicability, we applied Decoder-FFPE-seq to diverse archived pathological tissues using human whole-transcriptome probe panels (Fig. 3, A and B; fig. S12; and tables S7 and S8). In FFPE breast cancer sections, 50-μm–spot Decoder-FFPE-seq outperformed 55-μm–spot Visium CytAssist_FFPE in UMI and gene counts detected per spot. Its sensitivity in breast cancer and lymph node FFPE tissues even exceeded that of Visium for FF samples. Increasing probe multiplicity from average one to three pairs per transcript enhanced lymph node detection from 30,140 UMIs/7770 genes per spot to 103,501 UMIs/12,971 genes (Fig. 3A and fig. S12B), demonstrating that sensitivity can be tuned via multiregion probe targeting. Notably, Decoder-FFPE-seq detected a median of 10,625 UMIs and 3951 genes per spot in a 6-year-old OSCC FFPE sample using a single probe-pair design (Fig. 3A and fig. S12C).

**Fig. 3.**
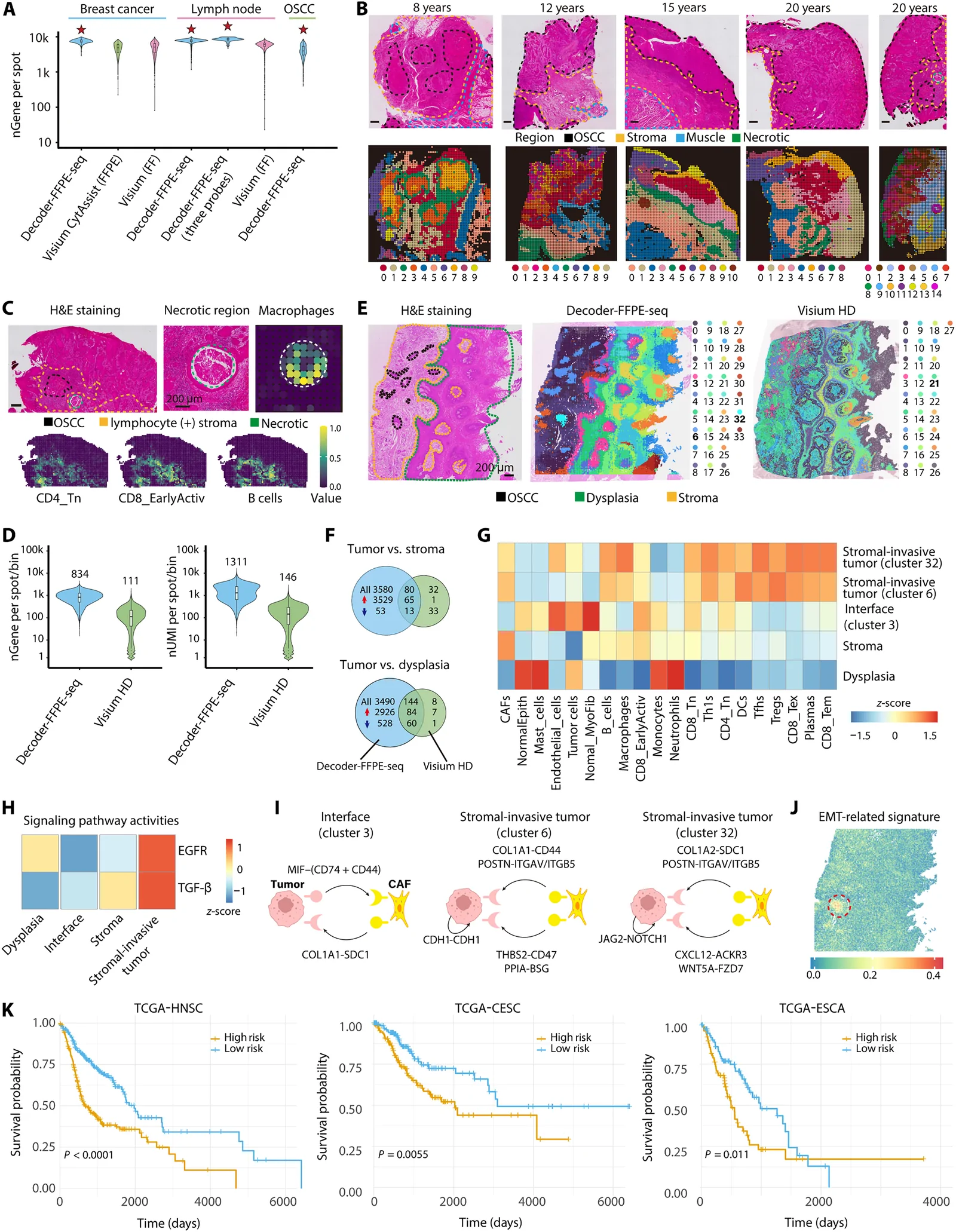
Decoder-FFPE-seq characterizes complex TME in long-term archived clinical tissues. (**A**) Distribution of nGene per spot in FFPE breast cancer, lymph node, and OSCC tissues profiled by Decoder-FFPE-seq, compared with FFPE and FF breast cancer samples profiled by Visium CytAssist and Visium. Red stars indicate Decoder-FFPE-seq performance. (**B**) H&E images with pathological annotations (top) and unsupervised clustering of Decoder-FFPE-seq spots from OSCC samples preserved for 8 to 20 years. Tumor (black), stroma (yellow) and muscle (blue), and necrotic (green). Scale bars, 200 μm. (**C**) Representative H&E image of 20-year-old OSCC from Fig. 3B, with annotated necrotic area and lymphocyte aggregates and their corresponding cell distribution. The H&E image was rotated to better display tissue structure. Scale bar, 500 μm. (**D**) Distribution of nUMI (right) and nGene (left) per spot/bin from 8-μm–spot Decoder-FFPE-seq and 8-μm–bin Visium HD. (**E**) H&E image with pathological annotations (left), unsupervised clustering of OSCC based on Decoder-FFPE-seq (middle) and Visium HD. Tumor (black), dysplasia (green), stroma (yellow), muscle (blue), and vessel (read). Scale bar, 200 μm. (**F**) Venn diagrams showing the overlap of differentially expressed genes (DEGs) between tumor versus stroma (top) and tumor versus dysplasia (bottom) identified by Decoder-FFPE-seq and Visium HD. Numbers indicate shared and unique genes. (**G**) Heatmap of cell types across dysplastic, the dysplasia-stroma interface (cluster 3), stroma, and stromal-invasive tumor (clusters 32 and 6). (**H**) Standardized mean PROGENy pathway activities from dysplastc to interface (cluster 3) to stroma and stromal-invasive tumor. (**I**) Cell-cell interaction networks between tumor cells and cancer-associated fibroblasts (CAFs) at the dysplasia-stroma interface (left), invasive tumor cluster 6 (middle), and invasive tumor cluster 32 (right). Representative ligand-receptor pairs illustrate region-specific signaling interactions. (**J**) Spatial projection of the EMT-related signature on the OSCC section. (**K**) Kaplan-Meier survival curves comparing overall survival between high-risk and low-risk groups in TCGA-HNSC (left), TCGA-CESC (middle), and TCGA-ESCA (right) cohorts. *P* values were calculated using the log-rank test.

Extending the analysis to 8- to 20-year archived OSCC specimens, our approach consistently detected a median of more than 4000 genes per spot, even in 20-year samples of severe RNA degradation (DV200 = 17%) (fig. S12, D and E). Spatial clusters of these samples closely matched their H&E-annotated pathological regions, and cell-type deconvolution confirmed cluster identities (Fig. 3B and fig. S12F). For example, in the 20-year OSCC sample (DV200 = 17%), cluster 14 was localized to a necrotic tumor area, showing macrophage distribution, consistent with studies that the tumor-associated macrophage is enriched in hypoxic and necrotic TME (Fig. 3, B and C) (*28*). Meanwhile, clusters 2, 5, 6, and 8 were confined to lymphocyte (+) stromal regions, where diverse immune populations, such as CD4^+^/ CD8^+^ T cells and B cells, were accurately resolved, corresponding to lymphocyte aggregates on the H&E-stained section (Fig. 3, B and C). Besides, we evaluated six housekeeping genes (*UBC*, *PPIB*, *HRPT1*, *TPB*, *GUSB*, and *ALAS1*) in this sample. Their expression level from pseudobulk transcript counts of Decoder-FFPE-seq data aligned with bulk RNA-seq from TCGA-HNSC cohort and exhibited expected spatial distribution (fig. S13, A to C, and Materials and Methods), indicating that RNA degradation during extremely long-term preservation did not distort spatial expression patterns of Decoder-FFPE-seq. Low-abundance immune checkpoint genes were also effectively detected, including classic receptors (*PDCD1* and *HAVCR2*) and ligands (*CD274* and *LGALS9*) (fig. S13D). When focused on the 6-year-old OSCC specimen with lymph node metastasis, Decoder-FFPE-seq illustrated the spatial colocalization of CD163^+^ macrophages and FAP^+^ cancer-associated fibroblasts (CAFs) at the tumor-stroma interface, which had been validated by multiplex immunofluorescence (IF; fig. S14, A to D). This spatial interaction, potentially orchestrated by the CXCL12-CXCR4 axis, promoted an immunosuppressive TME and facilitated tumor progression (fig. S14, E to J). These results demonstrate Decoder-FFPE-seq’s robustness in unlocking spatial transcriptomes of challenging archival tissues, enabling retrospective studies of longitudinal patient cohort.

Furthermore, we applied 8-μm–spot Decoder-FFPE-seq on an archived OSCC sample encompassing moderate dysplasia epithelium, stroma, and stromal-invasive tumor and performed a direct head-to-head comparison with Visium HD on the adjacent tissue section. The 8-μm–spot Decoder-FFPE-seq consistently outperformed Visium HD, yielding an 8.9-fold increase in UMI detection and a 7.5-fold increase in gene counts per spot (Fig. 3D). This superior sensitivity remained robust across normalized sequencing depths and was further corroborated by the enhanced detection of cell-type–specific marker genes (fig. S15, A and B). Moreover, 8-μm–spot Decoder-FFPE-seq enabled finer spatial stratification, identifying 34 distinct clusters compared to 28 by Visium HD under identical spatial clustering parameters (Fig. 3E). Notably, 8-μm–spot Decoder-FFPE-seq resolved the stromal-invasive tumor into two distinct clusters (clusters 32 and 6) and identified a continuous, layer-like cluster at the dysplasia-stroma interface (cluster 3). In contrast, Visium HD detected only a single tumor cluster (cluster 21) and a more diffuse cluster that extended into the stroma at the comparable interface region (cluster 10) (Fig. 3E). Differential expression and GO enrichment analyses consistently demonstrated that 8-μm–spot Decoder-FFPE-seq identified a greater number and proportion of differentially expressed genes (DEGs), along with more significantly enriched pathways, than Visium HD when comparing stromal-invasive tumor to both stroma and dysplasia (Fig. 3F and fig. S15, C to E). For example, the top 20 enriched pathways identified by Visium HD were primarily associated with cell-matrix interactions, mechanical responses, and tissue remodeling, all of which were also consistently enriched in the Decoder-FFPE-seq dataset. Beyond these, Decoder-FFPE-seq effectively resolved additional transcriptionally active programs within the stromal-invasive tumor regions, including RNA processing, nuclear transport, and transforming growth factor–β (TGF-β) signaling (fig. S15E). Together, these results underscore the superior sensitivity and broader gene-detection capacity of Decoder-FFPE-seq, enabling more refined delineation of tumor heterogeneity and boundaries and revealing richer biological signals.

Using cell-type deconvolution, we characterized the cellular composition across dysplastic epithelium, the dysplasia-stroma interface (cluster 3), stroma, and stromal-invasive tumor with Decoder-FFPE-seq (Fig. 3G). The dysplasia-stroma interface exhibited a high proportion of tumor cells, endothelial cells, myofibroblasts, CD4^+^/CD8^+^ T_n_ cells, and CD8^+^ EarlyActive cells. In contrast, stromal-invasive tumor regions showed markedly increased immune infiltration, comprising both immunosuppressive [e.g., regulatory T cells (T_reg_ cells) and exhausted CD8^+^ T cells (CD8^+^ T_ex_ cells)] and immune-activated populations (e.g., B cells and CD8^+^ T_em_ cells). PROGENy pathway analysis revealed a V-shaped activity pattern for the epithelial-associated epidermal growth factor receptor (EGFR) pathway along the spatial axis from dysplasia to the interface (nadir), through the stroma, to the stromal-invasive tumor. This was accompanied by a progressive increase in EMT-related TGF-β signaling (Fig. 3H), a gradient distribution further supported by consistent CytoTRACE and EMT scores (fig. S16, A and B). These results may suggest that the dysplasia-stroma interface represents a transitional state with partial epithelial features, whereas the stromal-invasive tumor reflects a more advanced EMT and immunologically TME. This gradient distribution was not observed in the data of Visium HD when we selected the cluster 10 as a dysplasia-stroma interface (fig. S16C). Cell-cell interaction revealed that, at the interface, tumor cells and CAFs primarily interact via MIF–(CD74 + CD44) and COL1A1-SDC1 axes, along with autocrine signaling, reflecting a proinflammatory and matrix-remodeling niche (Fig. 3I and table S9). While in the stromal-invasive tumor region, CAF-tumor interactions further diverged. Cluster 6 involved COL1A1-CD44, POSTN–(ITGAV + ITGB5), THBS1-CD47, and PPIA-BSG interactions and tumor cell self-interaction via CDH1-CDH1, centered on matrix adhesion and immune modulation. Cluster 32 was dominated by the CXCL12-ACKR3, POSTN-ITGAV/ITGB5, COL1A2-SDC1, and WNT5A-FZD7 interactions, promoting chemotaxis, matrix remodeling, and stemness, with tumor cells maintaining stemlike properties via JAG2-NOTCH1 (Fig. 3I and table S9). These results show that Decoder-FFPE-seq resolves fine tissue structure and reveals a potential spatial transition in this OSCC sample from a proinflammatory, partially epithelial interface to an EMT- and stemness-driven invasive TME.

Given the divergent features between the two tumor clusters, we compared them and found that cluster 32 exhibited up-regulation of genes associated with extracellular matrix remodeling, invasion, and chemotactic signaling (e.g., *MMPs*, *TNC*, and *CXCL13*), along with increased EMT, apical junction, and interleukin-2 (IL-2)–signal transducer and activator of transcription 5 (STAT5) signaling pathways (fig. S16, D and E). These results further indicate that cluster 32 displays enhanced cell polarity remodeling, immune regulation, and mesenchymal transition, consistent with an aggressive phenotype in such stromal-invasive tumor region. Using univariate Cox, LASSO, and multivariate Cox analyses (*29*, *30*), we identified 18 key genes (*AMIGO2*, *ANO1*, *COL8A2*, *COPA*, *CRIP1*, *CXCL13*, *CXCR4*, *GALNT2*, *GSTO1*, *IKZF2*, *LAMC2*, *PHLDA1*, *PPIL4*, *RNF217*, *RNPEP*, *TGFBI*, *VAV3*, and *WNT4*) from the up-regulated genes. Spatial projection confirmed the gene set enrichment in the stromal-invasive tumor region of cluster 32 (Fig. 3J), and functional enrichment analysis revealed that it was significantly associated with EMT (*P* = 0.02; fig. S16F), a key driver of tumor invasion and poor prognosis. Accordingly, when applied to TCGA-HNSC, CESC, and ESCA cohorts, this EMT-associated signature effectively stratified patients into high- and low-risk groups (Fig. 3K). In contrast, as Visium HD identified only a single tumor cluster, we attempted to derive a similar gene set by comparing stromal-invasive tumor and dysplasia regions but recovered only 2 of the 18 genes (fig. S16G). Collectively, Decoder-FFPE-seq effectively resolves a potential invasion-associated tumor cluster in OSCC and uncovers an EMT-related signature associated with clinical outcomes across multiple squamous cell carcinomas.

### Extending ST to non–poly(A)-tailed and nonhost RNA detection for spatial biology study in archival OSCC samples

Current understanding of transcriptional regulation remains biased toward protein-coding genes despite the critical contributions of noncoding RNAs (ncRNAs) and pathogenic RNAs to gene regulation and disease progression (*31*–*33*). Spatial profiling of these RNAs has been hindered by their low abundance, partial lack of polyadenylation, or, in the case of infectious agents, the frequent restriction of samples to FFPE-archived tissues. Leveraging the flexible probe design of Decoder-FFPE-seq, we extended its scope to capture both non–poly(A)-tailed and nonhost RNAs, enabling the joint profiling of mRNA-lncRNA and host mRNA-pathogenic RNAs in archival OSCC samples (Fig. 4A).

**Fig. 4.**
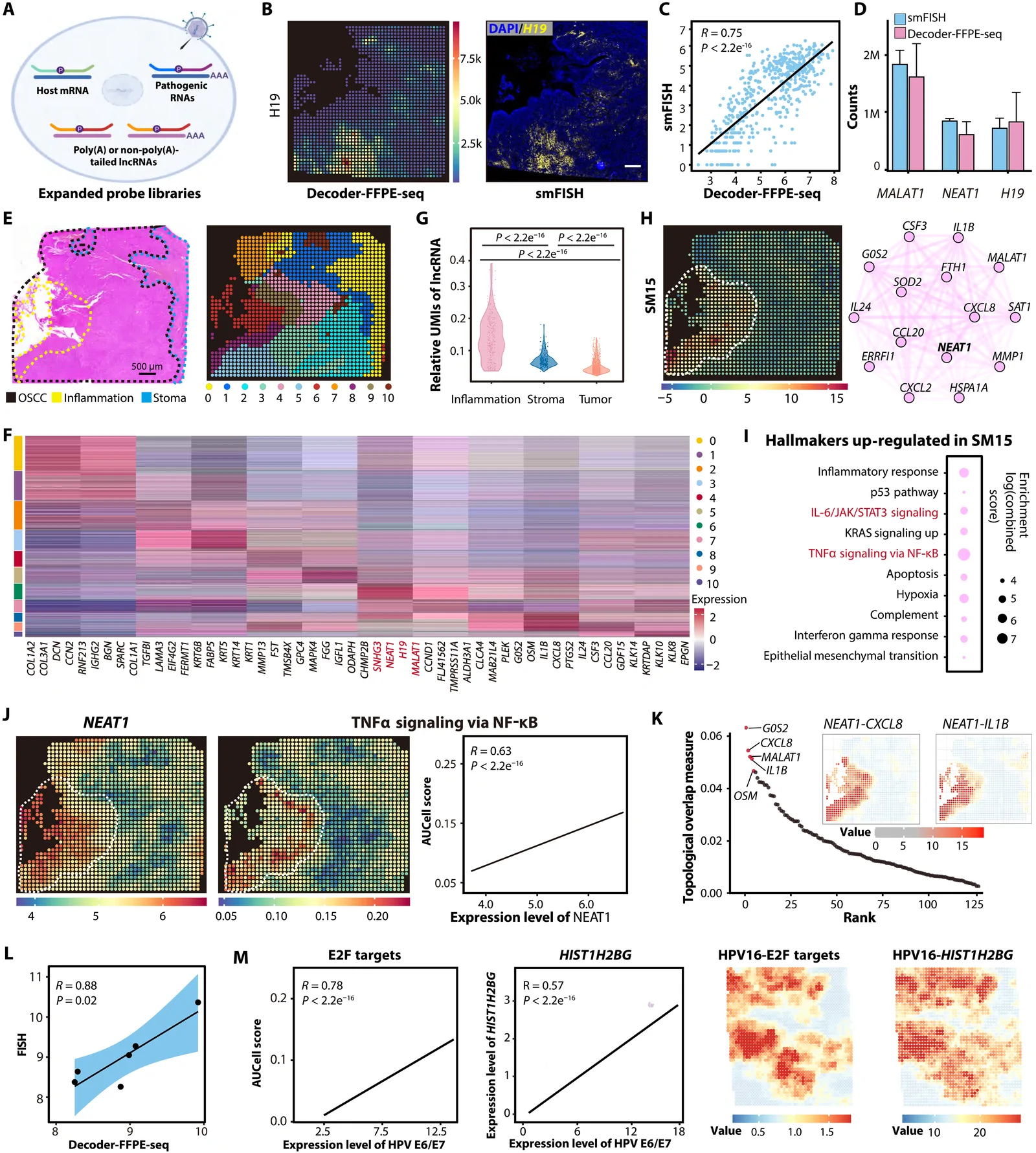
Spatial coprofiling of mRNA-lncRNA and host mRNA–human papillomavirus RNA in OSCC archives. (**A**) Schematic of expanded probe libraries for spatial coprofiling in Decoder-FFPE-seq. Created in BioRender. J.Ze. (2026); https://BioRender.com/tfm920x. (**B**) Spatial distribution of *H19* from Decoder-FFPE-seq and single-molecule FISH (smFISH) in adjacent sections. Scale bar, 500 μm. (**C**) Gene consistency between Decoder-FFPE-seq and smFISH (*R* = 0.75). (**D**) Comparison of representative lncRNA counts detected by Decoder-FFPE-seq and smFISH (*n* = 3). (**E**) H&E image (top) of OSCC section with pathological annotations and its unsupervised clustering (bottom). OSCC (black), stroma (blue), and tumor-associated inflammatory region (yellow). (**F**) Top-ranked DEGs defining each cluster. (**G**) Relative lncRNA expression across annotated regions. (**H**) Spatial distribution of spatial module 15 (SM15; right) and its coexpression network of top 15 hub genes (left). (**I**) Hallmark pathways enriched in SM15. (**J**) Spatial distributions of *NEAT1* (left), tumor necrosis factor–α (TNFα)–nuclear factor κB (NF-κB) signaling expression, and their interactions (right). (**K**) Topological similarity of *NEAT1* with other genes in SM15, and spatial mapping showed the spatial colocalization of *NEAT1* with inflammation-related genes. (**L**) Correlation of HPV transcript counts between Decoder-FFPE-seq and human papillomavirus (HPV)–FISH (*n* = 6 matched regions). (**M**) Spatial correlation of E2F pathway activity and downstream gene expression with HPV expression.

LncRNAs exhibit tissue- and cancer-specific expressions and regulate mRNA to promote oncogenesis through diverse mechanisms (*34*, *35*). We further designed a probe panel targeting 275 OSCC-associated lncRNAs (Materials and Methods and table S10). Using the lncRNA-only panel, Decoder-FFPE-seq detected all 275 targets, with a median of 3358 UMIs and 114 genes per 50-μm spot (fig. S17A). LncRNA expression counts reasonably correlated with bulk RNA-seq data from the TCGA-HNSC cohort (*R* = 0.55; fig. S17B). Representative lncRNAs (*H19*, *NEAT1*, and *MALAT1*) showed consistent spatial distribution with those measured by gold-standard single-molecule FISH (smFISH) in adjacent OSCC sections, exhibiting correlation coefficients exceeding 0.50 (Fig. 4, B and C, and fig. S17C). Quantitative comparisons of three gene counts showed that Decoder-FFPE-seq recovered 92.0 ± 17.1% of numbers detected by smFISH (Fig. 4D and table S11), confirming its high sensitivity.

We next explored the regulatory roles of mRNA-lncRNA interactions in OSCC using a combined mRNA-lncRNA panel (fig. S17D). Unsupervised clustering identified 11 spatially organized clusters with distinct marker gene expression, corresponding to pathologically annotated tumor, stroma, and tumor-associated inflammatory regions (Fig. 4, E and F). Notably, relative lncRNA expression was markedly enriched in the tumor-associated inflammatory region over stromal or other tumor compartments (Fig. 4G), potentially indicating regulatory roles of lncRNAs in such TME. For example, cluster 6 was localized to the inflammatory region and showed high expression of *SNHG3*, *NEAT1*, *H19*, and *MALAT1* (Fig. 4, E and F). Besides, differential expression analysis revealed up-regulated and down-regulated lncRNAs in tumor versus nontumor regions (fig. S17E). As expected, oncogenic lncRNA H19 and tumor suppressor MEG3 were up-regulated and down-regulated in tumors, respectively, consistent with their established role (fig. S17, E and F) (*36*, *37*). Coexpression network analysis with hdWGCNA identified 43 spatial modules (SMs), of which SM1 and SM15 contained multiple lncRNAs as hub genes with high connectivity with other transcripts (fig. S18, A and B). Specifically, SM15 was localized to the tumor-associated inflammatory region and contained hub genes such as the oncogenic lncRNA *NEAT1*, inflammatory mediators *CXCL8* and *CCL20*, and oxidative stress regulators *FTH1* and *SOD2* (Fig. 4H). Functional enrichment confirmed that SM15 was strongly associated with inflammatory pathways, including tumor necrosis factor–α (TNFα)–nuclear factor κB (NF-κB), inflammatory response, and IL-6/Janus kinase/STAT3 (Fig. 4I). Spatial projection of the TNFα–NF-κB pathway overlapped with *NEAT1* expression, yielding correlation coefficients of 0.63 (Fig. 4J), suggesting that *NEAT1* may be involved in this signaling axis. Besides, spatial coexpression network analysis revealed the highest topological similarity of *NEAT1* with inflammation-related genes (*IL1B* and *CXCL8*) and the cell cycle regulator *G0S2*, showing spatial colocalization (Fig. 4K). These findings suggest that *NEAT1* may serve as a hub gene in the coexpression network of mRNA-lncRNA, amplify TNFα signaling to drive inflammatory gene expression (e.g., *IL1B* and *CXCL8*), and coordinate inflammatory responses and tumor progression (*38*, *39*).

High-risk human papillomavirus (HPV)16/18 are well-established drivers of OSCC through expression of E6/E7 oncoproteins (*40*). We designed HPV16/18-specific E6/E7 probes for coprofiling host transcriptome and viral E6/E7 transcripts in an HPV-positive OSCC sample (Materials and Methods and table S12). Spatial expression of E6/E7 transcripts was confined to tumor regions, and their UMI counts strongly correlated with FISH measurements (*R* = 0.88; Fig. 4L and fig. S18C), validating the accuracy of Decoder-FFPE-seq in profiling pathogen RNAs. Unsupervised clustering identified distinct clusters (fig. S18D), and functional enrichment revealed that tumor clusters exhibited pronounced up-regulation of HALLMARK_E2F_TARGETS and MYC_TARGETS_V1 compared to the stroma (fig. S18E). Of note, E2F target and its downstream histone- and cell cycle–related genes (*HIST1H3G*, *HIST1H2BG*, and *MKI67*) positively correlated with E6/E7 transcript abundance (Fig. 4M and fig. S18F). These results are consistent with the known roles of E6/E7 proteins in p53 and pRb inactivation, aberrant E2F activation, and uncontrolled cell cycle progression (*41*). Together, these findings demonstrate the capacity of Decoder-FFPE-seq in the dissection of multilayer transcriptional interactions and host-pathogen regulatory networks in clinical archived samples, advancing spatial RNA biology into previously challenging domains.

### Decoder-FFPE-seq identifies pathological response–related spatial niches after immunotherapy in OSCC

ICB has achieved major clinical success in oncology, yet therapeutic responses vary widely, and the determinants of these divergent outcomes remain incompletely understood (*42*–*44*). To explore this, we applied 50-μm–spot Decoder-FFPE-seq to seven posttreatment FFPE specimens from patients who were diagnosed with locally advanced OSCC and enrolled in neoadjuvant anti–PD-1 clinical trials (Fig. 5A, fig. S19, and table S13). On the basis of residual tumor regression, samples were classified as major pathological response (MPR; *n* = 3), pathological partial response (pPR; *n* = 2), and pathological nonresponse (pNR; *n* = 2) (fig. S20A).

**Fig. 5.**
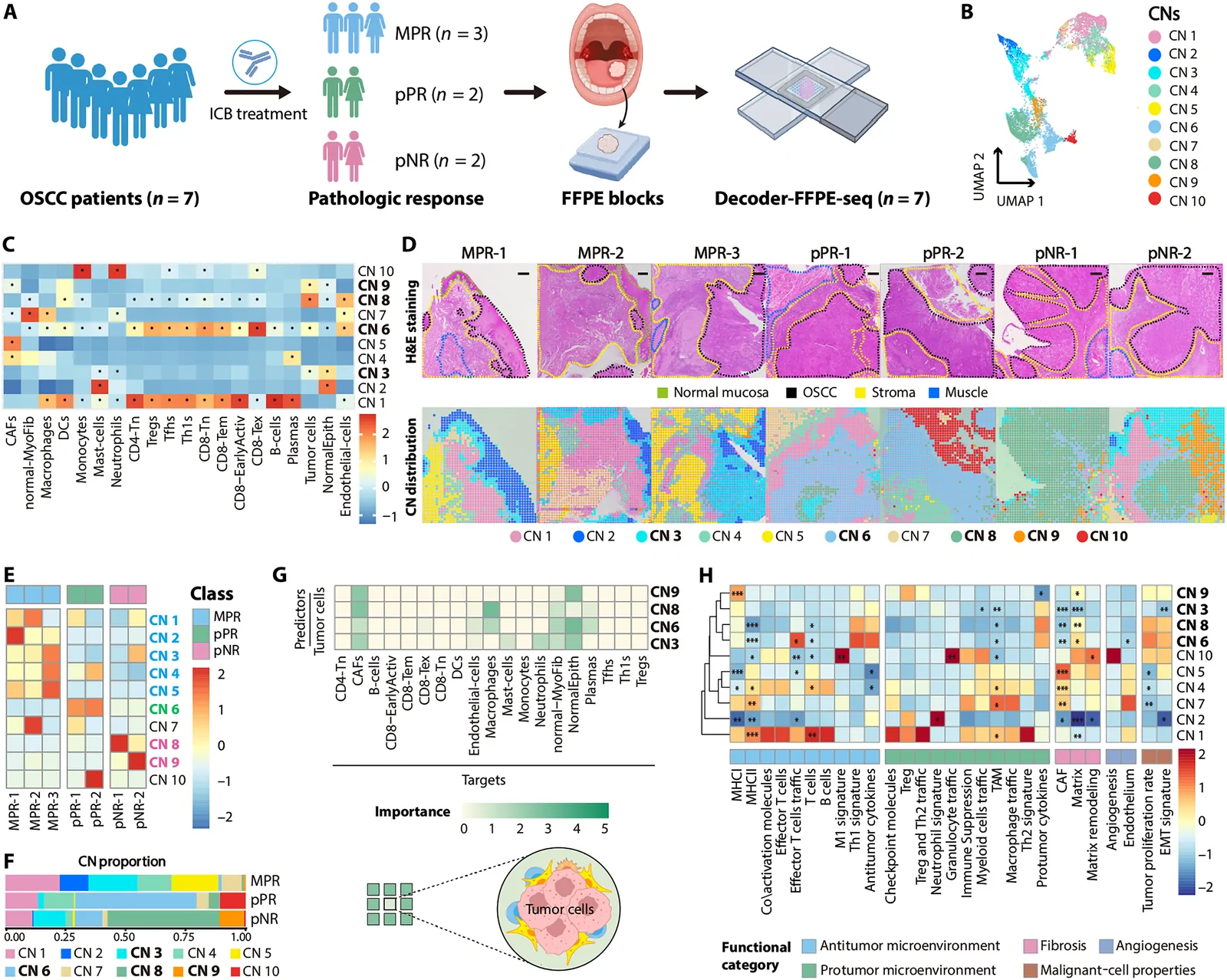
Decoder-FFPE-seq identifies response-related spatial niches after immunotherapy in OSCC. (**A**) Workflow of OSCC sample collection and processing for Decoder-FFPE-seq. Created in BioRender. jun, Z. (2026) https://BioRender.com/x1opsp8. (**B**) UMAP of cell-type niches (CNs) based on deconvolved cell-type compositions. (**C**) Scaled median cell-type composition for each CN. Asterisks indicate significantly increased abundance of a specific cell type compared with other CNs (two-sided Wilcoxon rank sum test; adjusted *P* < 0.05). (**D**) H&E images of seven tissue sections (top) with spatial projections of CNs (bottom). Pathologically annotated regions included OSCC (black), stroma (yellow), muscle (light blue), and normal mucosa (green). Scale bar, 500 μm. (**E** and **F**) Relative CN composition across each patient (E) and response groups (F). (**G**) Median importance of tumor cell abundance for predicting other cell types within ∼50-μm spots. (**H**) AUCell-based enrichment of tumor-associated pathways. Asterisks indicate pathways significantly up-regulated in a given CN compared with others (two-sided Wilcoxon rank sum test; adjusted *P* < 0.05).

To dissect spatial heterogeneity of the TME across pathology response groups, we applied cell-type deconvolution and unsupervised clustering to Decoder-FFPE-seq data from seven samples, resolving 10 major cell-type niches (CNs) (Fig. 5B and fig. 20B). Each CN displayed distinct cell-type compositions and spatial distributions that broadly aligned with pathology-defined regions, suggesting that they may represent functional building blocks of the TME (Fig. 5, C and D). CNs 3, 6, and 8 localized primarily to tumor regions, with CNs 6 and 8 representing immune-infiltrated tumor niches and CN 3 a mixed tumor-epithelium niche enriched for mast cells and neutrophils. Stromal regions contained CNs 1, 4, 5, 7, and 10. CN 1 was an immune-infiltrated stroma, CNs 4, 5, and 7 were CAF-rich, and CN 10 was an inflammation-associated stroma with abundant monocytes and neutrophils. CN 9 represented a CAF-rich tumor-stroma interface niche, while CN 2 corresponded to epithelial regions in normal mucosa. Notably, niche composition varied across response groups, particularly with tumor cell–enriched CNs (3, 6, 8, and 9) displaying distinct distribution patterns (Fig. 5, E and F). MPR tumors were enriched in the mixed tumor-epithelium CN 3, pPR tumors were dominated by CN 6, while pNR tumors contained predominantly tumor-enriched CNs 8 and 9 (Fig. 5F).

We next examined functional features of these niches and their association with treatment efficacy. We focused on tumor cell–enriched niches to dissect their heterogeneous spatial interaction patterns between malignant cells and nonneoplastic populations. Using relative tumor cell proportions, the abundances of other cell types were predicted across intraspot (∼50 μm) (Fig. 5G). In MPR groups, the dominant mixed tumor-epithelium CN 3 neighbored epithelial CN 2 and showed strong spatial co-occurrence of tumor cells with CAFs, endothelial cells, mast cells, neutrophils, epithelial cells, and myofibroblasts, suggesting a regression zone with localized inflammation responsive to ICB. In pPR tumors, the immune-infiltrated CN 6 predominated, where tumor cells most strongly predicted the presence of CAFs, endothelial cells, macrophages, myofibroblasts, epithelial cells, and plasma cells. Active interactions between tumor cells and immune-stromal components of the TME likely underlie the partial responsiveness to ICB. In contrast, pNR tumors were dominated by the following: (i) CN 8, where tumor cells predicted CAFs, macrophages, myofibroblasts, and plasma cells, reflecting an immunosuppressive TME associated with resistance; and (ii) CN 9, where tumor cells co-occurred with CAFs and epithelial cells at the tumor-stroma interface, suggesting roles in extracellular matrix (ECM) remodeling and immune evasion.

Gene set enrichment confirmed niche-specific functional programs (Fig. 5H). In MPR tumors, CN 3 showed reduced malignant potential, with low proliferative activity and significantly diminished EMT signatures. In pPR tumors, CN 6 exhibited elevated effector T cell trafficking, T helper 1 cell (T_H_1 cell) signaling, and antitumor cytokine expression, consistent with immune engagement. Meanwhile, CN 6 up-regulated proliferative pathways (E2F, MYC, and G_2_-M cell-cycle progression), TGF-β signaling, and inflammatory programs (fig. S20C), reflecting complex tumor-immune cross-talk that may constrain aggressive growth. By contrast, pNR-dominant CN 8 showed elevated protumor cytokines and reduced antitumor cytokines, consistent with ineffective immune activity (Fig. 5H). GO enrichment of DEGs between CN 6 and CN 8 further supported this divergence (fig. S20D). CN 9 was marked by a strong T_reg_ cell signature, supporting its role in immunosuppression (Fig. 5H). Stromal niches also exhibited distinct behaviors. CN 1, an immune-infiltrated stroma, up-regulated interferon-α/γ responses and displayed partial antitumor activity (fig. S20E). While both CNs 4 and 5 were CAF rich, CN 4 facilitated immune infiltration, whereas CN 5 was ECM enriched and promoted matrix remodeling (fig. S20F). The predominance of CN 5 in MPR tumors suggests a “tissue reconstruction” zone undergoing regression, whereas CNs 1 and 4, observed in both MPR and pPR, likely represent immune-activated stromal regions. Collectively, Decoder-FFPE-seq resolves spatial organization of heterogeneous TME with distinct functional states across pathology response groups.

### Decoder-FFPE-seq decodes spatial cellular dynamics in OSCC before and after chemoimmunotherapy

Cellular interactions within the TME before treatment may dictate immune responses to ICB (*44*, *45*). To elucidate how the TME evolves under therapy and the molecular mechanisms underlying these changes, we sequenced pretreatment biopsies from patients with OSCC and integrated them with matched posttreatment data (fig. S21). Because different ICB regimens may differentially modulate immune responses, paired spatial analyses for examining TME dynamics were restricted to patients receiving the same treatment (chemoimmunotherapy; MPR and pNR-1 groups; Fig. 6A).

**Fig. 6.**
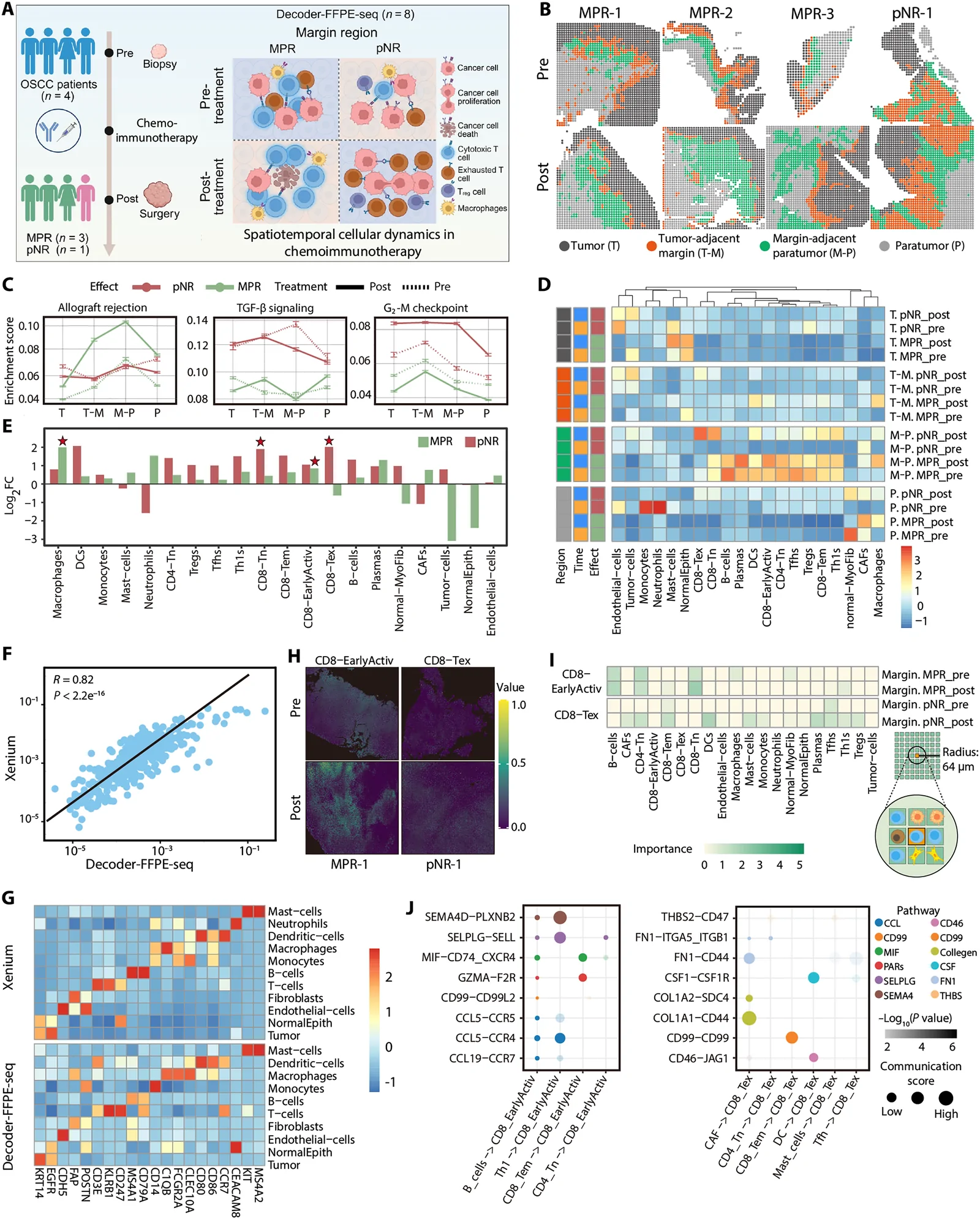
Decoder-FFPE-seq decodes spatial cellular dynamics in OSCC samples before and after chemoimmunotherapy. (**A**) Schematic diagram of sample collection, processing, and spatial profiling. Created in BioRender. J.Ze. (2026); https://BioRender.com/hcxz74n. (**B**) Schematic diagram of the regional division (left) and spatial distribution of distinct regions in OSCC samples before and after chemoimmunotherapy (right). (**C**) Hallmark pathway dynamic in different spatial regions before and after treatment among the two groups. (**D**) Heatmap of baseline and posttreatment cellular abundance across tissue regions. (**E**) Treatment-induced cellular abundance changes in the M region. (**F**) Comparison of gene expression levels between Decoder-FFPE-seq (UMIs per spot) and Xenium (transcripts per cell). (**G**) Marker gene expression across cell types between Xenium and Decoder-FFPE-seq. (**H**) Spatial distribution of CD8^+^ EarlyActive in MPR-1 and CD8^+^ T_ex_ cells in pNR-1 samples before and after treatment from 8-μm–spot Decoder-FFPE-seq. (**I**) Median importance of CD8^+^ EarlyActive in MPR-1 and CD8^+^ T_ex_ cells in pNR-1 abundance for predicting other cell types within a 64-μm radius. (**J**) Cell-cell communication analysis of CD8^+^ EarlyActive (MPR-1) and CD8^+^ T_ex_ (pNR-1) cells with their neighboring cells within the M region.

To systematically characterize the TME dynamics in OSCC before and after chemoimmunotherapy, we compared these groups at both molecular and compositional levels across distinct spatial zones: tumor (T), tumor-adjacent margin (T-M), margin-adjacent paratumor (M-P), and paratumor (P). These spatial zones were independently defined in each group via unsupervised clustering and tumor border annotation by H&E staining (Fig. 6B and fig. S22). Pathway analysis revealed marked spatial transcriptional heterogeneity across groups (Fig. 6C and fig. S23A). Following chemoimmunotherapy, pathways linked to allograft rejection and IL-2/STAT5 signaling were strongly up-regulated in MPR patients, particularly within T-M and M-P regions. In contrast, these pathways were down-regulated in pNR-1, which instead showed consistently enrichment of TGF-β signaling and proliferative programs (E2F targets, G_2_-M checkpoint, MYC targets, and DNA repair). Thus, treatment induced immune activation in the M region of MPR patients, whereas pNR-1 retained immunosuppressive and proliferative features.

We then calculated the baseline (pretreatment) cellular abundance and cellular dynamics (defined as the difference between the posttreatment and baseline abundance) based on cell-type deconvolution (fig. S23B). At baseline, MPR patients displayed strong immune cell infiltration in the M-P region, whereas the pNR-1 sample showed a myeloid-dominated microenvironment enriched with monocytes and neutrophils in the P region (Fig. 6D). The baseline cellular difference may underlie their divergent therapeutic outcomes, with responders displaying a stronger T cell–mediated immune response that established a favorable antitumor front adjacent to the tumor, consistent with observations in corresponding colorectal cancer responders (fig. S23C) (*46*). Posttreatment, MPR patients showed further immune cell enrichment in this region, with macrophages, DCs, and CD8^+^ EarlyActive cells elevated in T-M. In contrast, pNR-1 displayed increased CD8^+^ T_ex_ cells, CD8^+^/CD4^+^ T_n_ cells, DCs, CD8^+^ T_em_ cells, T_H_1 cells, T_reg_ cells, and macrophages in the M-P region (Fig. 6D and fig. S23D). Notably, macrophages increased most in post-MPR M regions, with CD8^+^ EarlyActive as the most elevated T cell subset, whereas CD8^+^ T_ex_ cells, DCs, and CD8^+^ T_n_ cells exhibited the largest increase in the M region of the pNR-1 patient (Fig. 6E). These findings indicate that chemoimmunotherapy promoted a CD8^+^ T cell–driven antitumor TME in the key M region of MPR groups, whereas the pNR-1 patient maintained an immunosuppressive TME, which resulted in tumor cell expansion and T cell exhaustion.

As the multicellular resolution of 50-μm–spot Decoder-FFPE-seq limits its ability to precisely separate closely coexisting cell types. We further applied 8-μm–spot Decoder-FFPE-seq to explore intercellular communications in TMEs at high resolution. To ensure methodological robustness, we first benchmarked it against Xenium, confirming its sensitivity and accuracy in cellular-resolution profiling of clinical tissues. We subsetted the post MPR-1 8-μm–spot Decoder-FFPE-seq data from >18,000 genes to the 417 genes shared with the Xenium panel (Materials and Methods and table S14) and compare their gene detection performance in the corresponding common capture region (fig. S24A). UMIs per spot from Decoder-FFPE-seq were comparable to Xenium’s transcripts per cell (Fig. 6F). Decoder-FFPE-seq and Xenium exhibited concordant spatial gene expression and cell-type distribution (Fig. 6G), exemplified by the B cells and tumor cells, as well as their corresponding markers (fig. S24, B and C). These results indicate that Decoder-FFPE-seq yields spatial results that comparable to an imaging-based ST.

Using 8-μm–spot Decoder-FFPE-seq, we characterized the key M regions of four representative samples (pre–MPR-1, post–MPR-1, pre–pNR-1, and post–pNR-1; fig. S25). Similarly, we identified distinct spatial regions in four samples through unsupervised clustering combined with tumor border annotations from H&E staining (fig. S26), followed by cell-type deconvolution to characterize treatment-induced cellular dynamics. Cell distributions were largely concordant with those derived from 50-μm–spot FFPE-seq (Fig. 6H and fig. S27 and S28). As with 50-μm data, CD8^+^ EarlyActive expanded in post–MPR-1, whereas CD8^+^ T_ex_ cells increased in post–pNR-1 (Fig. 6H). These populations displayed notable shifts in spatial interaction patterns within M regions before and after treatment (Fig. 6I). In MPR-1, CD8^+^ EarlyActive cells were initially predictive of B cells, CD4^+^/CD8^+^ T_n_ cells, and macrophages, but posttreatment, they additionally colocalized with B, CD8^+^ T_em_, and T_H_1 cells. In pNR-1, CD8^+^ T_ex_ cells transitioned from colocalizing with T follicular helper cell (T_FH_ cells) and CD8^+^ T_em_ cells to a broad network including CAFs, CD4^+^ T_n_ cells, CD8^+^ T_em_ cells, DCs, mast cells, monocytes, plasma cells, T_FH_ cells, T_H_1 cells, and T_reg_ cells. These changes may suggest responders progressed from immune priming to effector maturation underwent treatment, while nonresponders developed an immunosuppressive TME driving T cell exhaustion. Cell-cell communication analysis confirmed their spatial interactions within the M region (Fig. 6J). In MPR-1, CD8^+^ EarlyActive robustly interacted with both B cells and T_H_1 cells via ligand-receptor pairs such as SEMA4D-PLXNB2, SELPLG-SELL, and CCL-related pairs including CCL5-CCR5, CCL19-CCR7 and CCL5-CCR4, suggesting enhanced CD8^+^T cell recruitment and activation. Xenium data validated these enriched ligand-receptor interactions (fig. S29). In contrast, pNR-1 was characterized by predominant interactions of CD8^+^ exhausted T cells with CAFs (via FN1-CD44, COL1A1-CD44, and COL1A2-SDC4), reflecting a CAF-dominated immunosuppressive microenvironment. Overall, these findings may account for the divergent CD8^+^ T cell dynamics in different response groups and highlight their role in shaping distinct therapeutic outcomes.

## DISCUSSION

ST has transformed our ability to map molecular and cellular organization within their original tissue contexts, offering powerful avenues for both basic biology and clinical pathology. However, most ST methods depend on high RNA integrity (RIN > 7) to obtain high-quality data and are therefore largely restricted to FF material, which requires stringent cold-chain storage and often lacks long-term clinical follow-up. By contrast, FFPE repositories are stable at room temperature for decades and linked to rich clinical metadata, but their heavily cross-linked and fragmented RNAs have limited ST adoption. Existing FFPE-compatible ST approaches, therefore, force trade-offs among throughput, sensitivity, spatial resolution, and accessibility. Decoder-FFPE-seq addresses these constraints by integrating specific RNA-DNA probe conversion, high-efficient blotting-assisted spatial barcoding, and high-throughput sequencing into a standardized, open workflow. This combination enables sensitive, genome-wide spatial profiling of archival tissues at single-cell resolution while remaining implementable with common laboratory equipment and reagents.

Decoder-FFPE-seq retrieves spatial RNA data with sensitivity and genome-wide coverage. The split-probe design targets one or multiple non-poly(A) regions per transcript, converting short RNA fragments into amplifiable DNA probes with high specificity and efficiency (table S15) while minimizing capture of DNAs and abundant, low-information rRNAs (an important limitation of random-priming or in situ polyadenylation strategies). Paired with blotting onto our nanostructured Decoder slides, which provide ∼10-fold higher barcode density than comparable arrays, this approach prevents barcode loss during harsh FFPE pretreatment and maximizes productive encounters between probes and spatial barcodes. Decoder-FFPE-seq detects ∼722 UMIs per 8-μm spot in mouse brain (approximately 2.63 to 4.63 times higher than existing platforms). This high sensitivity allows for the precise mapping of low-abundance nonsensory GPCRs and the discovery of *Gpr83*-specific colocalization with GABAergic interneurons, which is not reliably captured by Stereo-seq V2 and Visium HD.

Beyond polyadenylated mRNAs, Decoder-FFPE-seq extends ST to non–poly(A)-tailed regulatory RNAs and nonhost transcripts. Using tailored probe panels, we jointly profiled mRNAs, OSCC-associated lncRNAs, and HPV E6/E7 transcripts in archival tumors. This revealed spatial coexpression modules, such as SM15, in which *NEAT1* acts as a network hub linked to inflammatory mediators and oxidative stress genes. Our approach also showed that local viral RNA abundance correlates with the activation of host proliferation programs. These results demonstrate how RNA profiling in FFPE tissue can reveal multilayered regulatory landscapes that were previously challenging in mRNA-restricted spatial assays.

Decoder-FFPE-seq provides an accessible platform for single-cell–resolved ST studies, lowering technical barriers for a wider community of researchers. Array-based sequencing methods are particularly attractive because they use standard laboratory reagents and instruments while offering streamlined and time-efficient workflows. However, affordable barcode slide fabrication and open-source protocols remain scarce, limiting widespread adoption of FFPE-compatible, single-cell resolution ST. To bridge this gap, Decoder-FFPE-seq integrated high resolution Decoder slides with an accessible, open-source ST workflow and protocol tailored for FFPE samples. Using a microfluidics-assisted combinatorial barcoding strategy, Decoder slides can be fabricated cost effectively in standard laboratory environments following our established protocol (*21*). This approach effectively circumvents the substantial technical hurdles inherent in sequencing-based decoding in Stereo-seq V2 and the light-directed synthesis required for 10x Genomics’ Visium HD platform. Furthermore, we provide a comprehensive, step-by-step ST protocol in the Supplementary Materials, detailing tissue pretreatment, probe hybridization and ligation, and spatial barcoding using both manual and instrument-based probe blotting. Key procedural insights and critical optimization steps are explicitly highlighted to ensure experimental robustness and reproducibility. Furthermore, the design specifications and core modules of the probe-blotting instrument are documented in Materials and Methods. By ensuring the open availability of both slide fabrication and experimental protocols, Decoder-FFPE-seq ensures broad accessibility and adoptability for widespread application across research communities.

Decoder-FFPE-seq unlocks the vast potential of archived FFPE collections for clinical and translational research. FFPE repositories, preserved for decades, hold unparalleled value: They are linked to detailed clinical records, encompass long-term outcomes, and include rare diseases and diverse pathological subtypes. However, RNAs in these samples further deteriorate over time, complicating spatial transcriptome recovery and reducing data fidelity. Decoder-FFPE-seq overcomes these barriers. We achieved spatial profiling in 20-year-old clinical samples, successfully recovering highly fragmented RNAs (DV200 = 17%) to identify tissue structure and immune populations. Through head-by-head comparison with Visium HD, 8-μm–spot Decoder-FFPE-seq consistently outperformed Visium HD in sensitivity (8.9-fold increase in UMI detection), identifying more DEGs and enriched pathways for gaining biologic insights. Decoder-FFPE-seq refined characterization of the dysplasia-stroma interface and stromal-invasive tumor heterogeneity, yielding an EMT-associated gene signature with prognostic value across HNSC, CESC, and ESCA that were not captured by Visium HD. Furthermore, in an ICB-treated trial cohort, Decoder-FFPE-seq resolved response-dependent TME heterogeneity and tracked spatial cellular dynamics underlying differential responses to chemoimmunotherapy. These findings demonstrate the power of Decoder-FFPE-seq to extract meaningful biological and clinical insights from deeply archived materials.

In summary, Decoder-FFPE-seq establishes a sensitive, genome-wide, single-cell–resolved, and broadly accessible ST framework for spatial biology studies. Looking forward, several refinements could further enhance its capabilities. Sensitivity may be increased through improved probe release and interfacial barcoding efficiency, such as electrophoretic probe release or DNA nanoengineering approaches to optimize barcode density and orientation (*47*, *48*). Subcellular resolution may be attainable by integrating high-resolution barcode slides (e.g., nanopatterned arrays) (*49*–*51*) or expansion microscopy into the blotting-assisted workflow (*52*, *53*). Expanding probe design to include additional targets, such as microRNAs, other ncRNAs [e.g., circular RNAs (*54*)], and nonhost transcripts, or applying total-transcriptome strategies (e.g., in situ polyadenylation and random primer capture), will broaden molecular coverage. Beyond transcriptomics, Decoder-FFPE-seq holds potential for spatial multiomics by incorporating proteomic or epigenomic tagging methods, enabling comapping of transcriptome, proteome, and epigenome within the same section (*55*–*57*). Clinically, scaling the approach across large patient cohorts and coupling results with artificial intelligence frameworks will facilitate the discovery of biomarkers, therapeutic targets, and predictive models (*58*). Together, these advancements position Decoder-FFPE-seq as a versatile foundation for next-generation spatial omics in biological research and precision medicine.

## MATERIALS AND METHODS

### Fabrication of Decoder slides

The comprehensive fabrication process, which uses microfluidic-assisted spatial barcoding on three-dimensional (3D) dendrimeric slides, has been detailed in our previous publication (*21*). Two types of microfluidic chips with different widths and channel numbers were designed: 50 channels × 50 μm and 192 channels × 8 μm. Microfluidic chips were fabricated using standard soft lithography. Chrome photomasks (CChip Scientific Instrument Co. Ltd., Suzhou, China) were used to pattern SU-8 photoresist on a silicon wafer, creating the mold. Polydimethylsiloxane prepolymer (curing agent A: base = 1:10) was poured onto the mold, degassed, cured (120°C for 20 min), peeled, and cut. A 1-mm metal punch pen was used to create inlet/outlet ports. 3D dendrimeric slides were prepared through sequential surface modifications: (i) piranha treatment (2 hours) for hydroxyl activation, (ii) glycidyloxypropyltrimethoxysilane silanization (5 hours), (iii) blocking with succinic anhydride/triethylamine (1 hour), and (iv) overnight PAMAM modification. For spatial barcoding, two microchannel chips were sequentially aligned over 3D dendrimeric slides perpendicular with each other. DNA barcode *X* (5-aminated) was covalently immobilized via disuccinimidyl suberate cross-linking, while DNA barcode *Y* was enzymatically ligated to *X* using T4 DNA ligase. This generated spatially defined *X_i_Y_j_* sequences at channel intersections.

### Design of probe libraries

Probe libraries targeting protein-coding genes of mouse and human transcriptomes were designed with reference to the 10x Genomics (www.10xgenomics.com/support/cn/cytassist-spatial-gene-expression/documentation/steps/probe-sets/visium-ffpe-probe-sets-overview) with certain modification. The mouse probe library has 19,763 probe pairs for 19,465 protein-coding genes (table S1). The human probe libraries contain 18,630 probe pairs for 17,943 protein-coding genes and 53,504 probe pairs for 18,085 genes (table S7).

We downloaded 333 OSCC-associated lncRNAs from the LncRNA and Disease Database (LncRNADisease3). Using the open-source software Oligo Designer Toolsuite (https://github.com/HelmholtzAI-Consultants-Munich/oligo-designer-toolsuite), corresponding lncRNA probes were designed in three steps: generation, filtering, and probe set selection for each gene. High-quality probes were successfully generated for 275 lncRNA genes (table S10). Detailed steps and parameters are provided in the Supplementary Materials.

For probes targeting E6 and E7 mRNAs of high-risk HPV types 16 and 18, we designed them by extending the original 16-nt probes to 25-nt ones based on reported HPV mRNA probes (table S12) (*59*). Probe filtering was conducted as abovementioned procedure. All probes targeting mRNAs, lncRNAs, and HPV 16/18 were synthesized by Beijing Tsingke Biotech Co. Ltd. (Shanghai, China).

Most samples were profiled using whole-transcriptome probe panels, unless otherwise specified (e.g., lncRNA, mRNA + lncRNA, or mRNA + HPV RNA). For clinical samples, one lymph node section, long-term archived samples (8 to 20 years), and all 8-μm–spot datasets were generated using the three–paired-probe panel, whereas the remaining 50-μm–spot datasets used the one–paired-probe panel. Probe panel information for representative samples was provided in table S15.

### Collection and preparation of FFPE mouse tissues

The whole brain was harvested from euthanizing male C57BL/6J mice (6 to 8 weeks old; Shanghai JieSiJie Laboratory Animal Co. Ltd.), rinsed with phosphate-buffered saline (PBS), and dissected into two regions: the MOB and the main brain. All tissues were fixed in 4% paraformaldehyde for 24 hours and embedded in low temperature melting paraffin. Animal procedures compiled with ethical regulations approved by the Laboratory Animal Welfare Ethics Committee (ACU-0501).

### Collection of FFPE patient specimens

Clinically FFPE tissue blocks, originally collected by physicians for diagnostic purposes, were sourced from the Department of Pathology, Renji Hospital Affiliated to Shanghai Jiao Tong University School of Medicine, with the approval of its Ethics Committee (KY2023-013-B), and School and Hospital of Stomatology, Wuhan University, with the approval of the Ethics Committee (WDKQ2025.B42, [2020] Ethics No. 2). Each donor provided written informed consent before participation. Except the breast cancer block, all other FFPE blocks were obtained from the School and Hospital of Stomatology, Wuhan University.

OSCC samples encompassed two distinct cohorts: (i) 10 clinically archival OSCC samples stored for different years, including one HPV-positive case, which were sourced from the Department of Oral Histopathology; and (ii) 12 FFPE specimen blocks derived from seven ICB-treated patients as a subgroup of 68 enrollees in the two-arm, randomized phase 2 trial (NCT04649476). These samples were sourced from the Department of Oral and Maxillofacial Surgery. All the patients in this cohort received neoadjuvant therapy with 3 cycles of camrelizumab with or without 2 cycles of TPF chemotherapy (docetaxel, cisplatin, and 5-fluorouracil), followed by curative surgery and adjuvant therapy. From these patients, one nonmetastatic lymph node from neck dissection, seven pretreatment biopsies, and four paired posttreatment samples from surgically resected tissues were obtained.

### Decoder-FFPE-seq protocol

A detailed protocol is provided in the Supplementary Materials, and all the study regents are listed in table S2, S16, and S17.

### Probe-blotting workflow and instrument development

Probe blotting can be performed either manually (fig. S2, A to E) or using an instrument (fig. S2, B to D and F). Briefly, a 10-μm-thick film spacer was affixed around the barcoded array on the Decoder slide (fig. S2A, a), and the mixed probe-blotting reagents were added into the spacer. The tissue slide with H&E staining and Decoder slide were then aligned either manually (fig. S2A, b and c) or instrumentally, with the tissue slide placed on top and the Decoder slide below, ensuring that the region of interest was aligned with the barcoded array (fig. S2, A to D). After incubating at 37°C for 30 min, the slides were separated, and the Decoder slide was washed to remove excess reagents before library preparation. Detailed probe-blotting protocol is provided in the Supplementary Materials.

To avoid technical variability caused by manual operation and to accurately record the probe-blotted tissue region, we designed and custom built a probe-blotting instrument (Dynamic Biosystems, China). This instrument consists of six main modules: tissue slide module, barcoded array slide module, temperature control module, imaging module, lifting module, and position alignment module (fig. S2, B to D). Specifically, the tissue slide and barcoded array module are designed to fix tissue slides and Decoder slides with clamps, respectively. The temperature control module with a temperature sensor is designed to control reaction for efficient probe releasing. The imaging module is camera equipped to take images of probe-blotted tissue regions for aligning spatial gene expression maps with corresponding tissue regions. The lifting and position alignment module are designed to align interested tissue regions with barcoded arrays on the Decoder slides and then adhere the two slides to trigger probe release reaction. The overall appearance of the instrument and an exploded view illustrating its individual modules are shown in fig. S2 (C and D). On the basis of the above design, the probe-blotting instrument can be fabricated through a professional instrument manufacturing company.

### Evaluation of the effect of tissue pretreatment and probe labeling on Decoder slides

To study the impact of key tissue pretreatment and probe labeling on spatial barcodes, Decoder slides were treated as follows: (i) baked at 42°C for 3 hours and then 60°C for 2 hours; (ii) de–cross-linked at 95°C for 1 hour; (iii) MOB-mounted Decoder slides had methanol fixation, Tween 20 permeabilization, overnight probe hybridization, and tissue removal. After treatment, Cy3-labeled poly(A) DNA hybridized with treated (*n* = 3) and untreated slides (*n* = 3) at 37°C for 30 min and then washed thoroughly. Last, the slides were imaged, and their fluorescence intensity was compared between the treated and untreated control groups (fig. S3).

### IF staining and smFISH

Multiplex IF staining of the FFPE section from a 6-year-old OSCC sample was performed by Shanghai Ruiyu Biotechnology Co. Ltd., and the slide was imaged and processed using the 3DHISTECH fluorescence imaging scanner. smFISH was performed with the SEERNA RNA FISH Detection Kit (Dynamic Biosystems, China) according to the manufacturer protocol, and the data were processed according to a previously reported study (*60*).

ISH assay for HPV E6/E7 mRNA was conducted on HPV-positive adjacent tissue sections using the HPV detection kit (Dynamic Biosystems, China) according to the manufacturer protocol. For localization analysis, the DAB-stained channels were isolated through RGB-to-HED color space conversion. Gaussian-like signals in the DAB channel images were detected using the Laplacian of Gaussian operator, and the 2D coordinates of all signal points were output. For quantitative analysis, six matched tissue regions were selected, and signal counts within each region were computed individually.

### Visium HD

The OSCC sample was sectioned, with one section used for Decoder-FFPE-seq and an adjacent section sent to the Shanghai Biotechnology Corporation for Visium HD assay based on manufacturer’s instructions (10x Genomics, CG000685). Sequenced reads were aligned to the Visium Human Transcriptome Probe Set v.2.0 and spatially mapped to the paired H&E images using Space Ranger v.3.0.0 (10x Genomics). The capture area of the Decoder-FFPE-seq sample was manually aligned with the Visium HD data using Loupe Browser v.9.0.0 (10x Genomics). All downstream analyses were performed in R using Seurat (v.5.4.0) and restricted to tissue regions shared between the two platforms to minimize potential bias.

### Xenium in situ high-plex assay

We designed a custom 50-gene panel targeting key markers of CAFs (e.g., collagen genes, FAP, and POSTN), tumor cells (e.g., keratins, cadherins, and EMT markers), and macrophages (e.g., SPP1 and immune-related human lymphocyte antigen genes), among others. This panel was integrated into the Xenium Human Immuno-Oncology Profiling Panel, resulting in a final 430-gene panel for the Xenium in situ high-plex assay (table S14). FFPE tissue sections (5 μm thickness) were mounted onto Xenium slides and processed according to the manufacturer’s protocol (Xenium In Situ for FFPE-Tissue Preparation Handbook. 10x Genomics). Cell segmentation was performed based on 4′,6-diamidino-2-phenylindole (DAPI)–stained nuclear morphology visualized in whole-slide scans acquired using the Xenium analyzer. Analyzer-generated output files were subsequently used for downstream data analyses.

### Data preprocessing of Decoder-FFPE-seq

DynamicST (v1.0.7) was developed to process Decoder-FFPE-seq sequencing data. For read 1, only barcodes *x* and *y* with a Levenshtein distance ≤ 1 from the whitelist were retained. UMIs sharing the same (*x*, *y*) barcode were clustered using Starcode-umi, and the relationships among read IDs, barcodes, and UMI clusters were recorded. On the basis of this mapping, a gene expression matrix of UMI counts per gene for each spatial barcode (*x*, *y*) was generated for downstream analyses. Transcriptome references included *Mus musculus* GRCm38 (mm10) and *Homo sapiens* GRCh38 (hg38), with probe set references “mouse” and “human.”

DynamicST-Assist (v1.2.1) was used for alignment with H&E-stained histological sections to precisely delineate tissue regions from background areas. Spots located outside the defined tissue boundaries were excluded, along with those exhibiting fewer than 2 UMI counts and 50 detected genes (note: the gene detection threshold was relaxed from 50 to 25 genes in 8-um spots). In addition, spots where the percentage of mitochondrial genes exceeded four SDs above the mean were removed. All quality control filtering and subsequent SCTransform normalization were performed using Seurat (v5.1.0) (*61*).

### Comparison of Decoder-FFPE-seq with similar ST platforms

MOBs, mouse brains, human breast cancer tissue, and lymph nodes were sequenced with Decoder-FFPE-seq, and detected gene counts and UMIs per spot per square micrometer were compared to those obtained from similar ST platforms, including Decoder-seq, 10x Visium Cytassist_FFPE, Patho-DBiT, Visium, Visium HD (8-μm bin), and Stereo-seq V2 (bin20; 10 μm). Spearman correlation analyses were conducted to assess the degree of similarity between the aggregate expression of each protein-coding gene obtained from Decoder-FFPE-seq and Decoder-seq. The function “cor.test” was used to assess the statistical significance of the correlation coefficient. Besides, expression abundance and percentage of marker genes for three subtypes were compared among 8-μm–spot Decoder-FFPE-seq, Visium HD, Stereo-seq V2, and a snRNA-seq reference dataset (*26*), within similar spatial ranges (cortex, hippocampus, and thalamus). Additional methods for benchmark were deposited in the Supplementary Materials.

### Molecule lateral diffusion analysis

We selected three regional-enriched genes (*Slc17a7*, *Hpca*, and *Ncdn*) to assess RNA lateral diffusion in Decoder-FFPE-seq (8 μm), Slide-seq V2 (10 μm), Visium HD (8-μm bin), and Stereo-seq V2 (bin20) by reported method (*22*, *62*). We identified consistent regions (500 μm by 300 μm) exhibiting specific expression of the marker genes across these four platforms. Each region was subdivided into six segments (500 μm by 50 μm) for repetition. We summed the UMI counts of each gene at 10-μm intervals (spanning a 50-μm width) along the 500-μm spatial axis. To characterize the distribution of expression, the aggregated expressions were aligned to their spatial coordinates, enabling profiling of peak expression locus and spatial spread. Subsequently, these summed values were normalized and depicted as density curves along the 500-μm axis, with the total area under each curve scaled to unity. Last, we computed the FWHM of the expression distribution as a metric for transcript diffusion and compared it to the actual wide of the corresponding region.

### Spatial profiling of nonsensory GPCRs in the mouse brain

The mouse nonsensory GPCR genes were obtained from the “Mouse GPCRs + Human and Rat Orthologs’” database (*63*) (https://esbl.nhlbi.nih.gov/Databases/GPCRs/), which comprises 790 nonolfactory GPCRs. Genes encoding opsin receptors, taste receptors (TAS1R/TAS2R), vomeronasal receptors, trace amine–associated receptor, and Mas-related GPCRs were excluded from subsequent analyses. GO biological process enrichment analysis conducted by R package clusterProfiler (*64*) (v4.10.1) was performed to functionally annotate the remaining GPCR genes. The significantly enriched GO terms (adjusted *P* < 0.001) were simplified by function “clusterProfiler:simplify” based on the semantic similarity and were subsequently clustered using function “enrichplot:pairwise_termsim” (v1.24.4). Last, R package ComplexHeatmap (v2.15.4) was applied for visualization.

To evaluate the performance of Decoder-FFPE-seq in detecting nonsensory GPCR genes, we compared its aggregated expression profile that normalized to average expression per micrometer with those obtained from Visium HD and Stereo-seq V2. Expression profile consistency between Decoder-FFPE-seq and MERFISH was confirmed by Spearman correlation, with parallel comparisons performed for Visium HD and Stereo-seq V2. Spatial expression patterns of representative GPCR genes were visualized across all four platforms.

Spatial expression patterns of representative GPCR genes were visualized across all four platforms. *Gpr83*^+^ and *Gpr83*^−^ spots were compared by DEGs and GO enrichment analysis. Colocalization of *Gpr83* with other inhibitory marker genes were also predicted by SpaGene (*65*).

### Spatial clustering and visualization

To detect spatial domains of sequencing sections, we first constructed a shared nearest neighbor graph based on the top 50 principal components derived from 2000 highly variable genes using the “FindNeighbors” function. Spatial clustering was then achieved by applying the Louvain algorithm through the “FindClusters” function. For visualization, we used the UMAP algorithm for dimensionality reduction. To identify domain-specific molecular signatures, we conducted Wilcoxon rank sum tests using the “FindAllMarkers” function to detect DEGs for each spatial domain. Last, domain annotation was accomplished according to their specific expression patterns of the canonical marker and the identified signatures, as well as the pathological annotation on the corresponding H&E sections. Distributions of spatial clusters and gene expression patterns were visualized using Seurat functions, “SpatialDimPlot” and “SpatialFeaturePlot,” respectively.

To enable a fair comparison, we analyzed the expression matrices of Decoder-FFPE-seq and Visium HD (both with 8-μm bin size) using identical parameters. We used the sketch algorithm implemented in Seurat, randomly sampling 30,000 cells to construct a representative subset. Clustering and dimensionality reduction were performed on this subset, and the resulting cluster labels and low-dimensional embeddings were projected to the full dataset using the ProjectData function. All comparative analyses were performed with identical parameter settings.

For the analysis of multiple ST datasets, particularly pre- and posttreatment OSCC samples, we applied canonical correlation analysis, a built-in Seurat function, to mitigate batch effects and implement data integration within each treatment group (pre- or posttreatment) before abovementioned clustering analysis.

### Spatial deconvolution of high-resolution mouse brain

We performed cell-type deconvolution on the mouse brain data of 8-μm–spot Decoder-FFPE-seq using Cell2location (*26*) (v0.1.4). The snRNA-seq reference encompassed the mouse whole cortex and hippocampus. The expected average number of cells per spot was set as 2, while other parameters followed the default settings. Following deconvolution, we quantified the cellular composition of each spot, retaining only cell types with predicted abundances exceeding 0.1 for downstream computations.

### PROGENy and CytoTRACE analysis

We applied PROGENy (*66*, *67*) (version 1.28.0) to spatially evaluate the activity of key signaling pathways using the top 100 typical target genes for each pathway. CytoTRACE2 (*67*) (version 1.1.0) was used to predict stemness potential across regions.

### Spatial deconvolution and cell-cell colocalization of OSCC

For OSCC datasets, we performed cell-type deconvolution using R package CARD (*68*) (v1.1). To construct an adequate scRNA-seq reference dataset, we specifically selected primary tumor specimens from dataset GSE234933 (*69*). In the dataset, low-quality cells and doublets were filtered out, T cells were annotated by the label “projecTIL_subtype” according to the original study, and subtypes of macrophages were classified into macrophages. In each cell population, cells ranking in the top 50% by UMI counts were selected and subsequently downsampled or upsampled to 1000 cells per state, yielding the single-cell expression matrices for 20 cell subtypes.

### Detection of housekeeping genes in data from archival samples

We quantified the expression of six housekeeping genes (*UBC*, *PPIB*, *HPRT1*, *TBP*, *GUSB*, and *ALAS1*) in a 20-year-old OSCC section at pseudobulk resolution. Pseudobulk samples were generated by applying *K*-nearest neighbors clustering based on spatial coordinates to group the spots into clusters of 50 spatially adjacent spots each, followed by summation of normalized expression values within each cluster. The pseudobulk expression levels of these genes were visualized using box plots and compared against bulk RNA-seq data of primary tumor samples in the TCGA-HNSC cohort, where the expression was normalized as transcripts per million.

### Weighted gene coexpression network analysis

We used hdWGCNA (*70*) (v0.4.0), the weighted gene coexpression network analysis tool for sparse data with high dimensions, to investigate the spatial interaction networks between lncRNA and mRNA in OSCC. All expressed features were used to infer the signed networks based on their spatial similarity patterns. Genes related to *NEAT1* were ranked by their connectivity with *NEAT1*, quantified by the topological overlap measure in the common gene module.

### Gene colocalization analysis

SpaGene (*65*) (v0.1.0) was used to evaluate the colocalization of lncRNA and mRNA of interests. Although originally developed to identify spatially variable genes and cell-cell communications, its basic principle is to detect genes colocalized in neighbors. This is achieved by quantifying the spatial connectivity of gene pairs in a subnetwork comprising only spots highly expressing the target genes. Thus, theoretically, SpaGene can estimate the colocalization of any gene pair. Specifically, “SpaGene_LR” function was used to identified if the pair of genes of interest were significantly colocalized, and “Plot_LR” function was used for visualization of the significant colocalization.

### Cell niche identification of OSCC

Spots exhibiting consistent cellular composition patterns across multiple samples were operationally defined as CNs, indicative of conserved structural organization (*71*). To systematically identify the CNs representing distinct pathological response groups, we implemented an analytical framework adapted from the literature (*71*). First, we calculated the isometric log-ratio–transformed cellular proportions for each spot. Next, we constructed a SNN graph using “scran::buildSNNGraph” function (*72*) (v1.30.2), followed by Leiden clustering to delineate distinct CNs.

### Analysis on tumor margin

To analyze the impact of immunochemotherapy on OSCC samples, spatial domains identified through unsupervised clustering (as described in the “Spatial clustering and visualization” section in Materials and Methods) were pathologically annotated on the basis of corresponding H&E-stained sections. This annotation process categorized tissue regions into four distinct compartments: T, T-M, M-P, and P. We then systematically evaluated (i) therapy-induced changes within the same compartments and (ii) differences between responsive and nonresponsive patients across these four regions, especially the margin sites.

### Test of differential cellular composition

Following the cellular deconvolution, the proportion or abundance of each cell type can be quantified within individual tissue spots. This enabled comparative assessment of cellular composition differences between distinct conditions or regions. Specifically, we identified variations in cell type fraction and determined the overrepresented cell populations within specific tissue niches or regions of interest. This differential analysis was conducted using the Wilcoxon rank sum test, with statistical significance defined as an adjusted *P* < 0.05 after multiple testing correction.

### Analysis of spatial dependency

CAF-macrophage colocalization of an archival OSCC specimen was quantified using Python package DeepCOLOR (https://github.com/kojikoji/deepcolor), which can calculate the colocalization strength of two given cell types in each spot from a single sample. To analyze spatial interactions across multiple samples, R package mistyR (*73*) (v1.10.0) was used to evaluate the spatial dependencies between tumor cells and other cellular components within tumor niches. This approach enabled quantitative characterization of complex spatial dependency among various cell types in TME. Using cell proportion estimates derived from CARD deconvolution across all samples, we constructed multiview analytical models to quantify interaction patterns. The analysis specifically assessed the significance of spatial interactions between various cell types that located (i) within an individual spot (intra view), (ii) between adjacent spots (juxta view), and (iii) in a 15-spot radius neighborhood (para view). We used 4-spot radius and 30-spot radius neighborhoods to respectively construct juxta and para views in analysis of high-resolution data. The median importance scores aggregated across all samples were interpreted as indicators of spatial dependency patterns, revealing either colocalization (positive association) or mutual exclusion (negative association) between cell types under these defined spatial contexts.

### Differential expression analysis

DEGs were identified using Seurat’s “FindMarkers” function with the Wilcoxon rank sum test. Genes meeting the following criteria were considered statistically significant: (i) adjusted *P* < 0.05 and (ii) absolute average log_2_ fold change ≥ 0.5. GOBP enrichment analysis was then performed using R package “clusterProfiler” to functionally characterize these DEGs.

### Gene set enrichment analysis

Single-spot gene set enrichment analysis was perform by R package irGSEA (*74*) (v3.3.2) using Hallmark gene sets, GOBP terms, and Biocarta pathways from the Molecular Signatures Database (*75*, *76*) (www.gsea-msigdb.org/gsea/msigdb/), tumor-associated gene sets defined by literature (*76*). AUCell score was used to quantify the enrichment level. To identify significant differences in gene signature expression between specific niches or regions of interest, FindMarkers function was used, following the same process described in the “Differential expression analysis” section in Materials and Methods. To ensure robust and reproducible identification of significantly enriched pathways, “irGSEA.integrate” function was applied to the enrichment scores calculated by AUCell, ssGSEA, UCell, and singscores.

### Analysis on colorectal cancer datasets

We analyzed a publicly available ST dataset (HRA006753) derived from patients with microsatellite instability colorectal cancer who subsequently responded to immunotherapy (*46*). Regions of each section were annotated using aforesaid method (see the “Analysis on tumor margin” section in Materials and Methods). Abundances of immunocytes in margin region were estimated by the average AUCell scores of corresponding cell type marker gene sets.

### Comparison to Xenium

We leveraged SpatialData (*77*) (v0.2.3) to aggregate Xenium transcripts into expression matrices at a single-cell level and to subset the data to align the same regions of 8-μm Decoder-FFPE-seq data. The data from both platforms were preprocessed by retaining only spots or cells that expressed more than 25 genes and genes detected in at least two spots or cells, resulting in a final expression profile set of 417 overlapped genes for downstream analysis. We calculated the Spearman correlation coefficient between numbers of UMIs per in square micrometer in Decoder-FFPE-seq and per cell in Xenium within shared genes. Moreover, cell type prediction was performed separately for spots from Decoder-FFPE-seq and cells from Xenium data using the “FindTransferAnchors” and “TransferData” functions in Seurat. The single-cell expression profile with the common genes from GSE234933, along with its original major cell state annotations, served as the reference. Last, we visualized the average expression levels of cell type-–specific markers across predicted cell types of Decoder-FFPE-seq and Xenium to evaluate concordance.

### Prediction of ligand-receptor interaction

COMMOT (*78*) (v0.0.3) and CellChat (*79*) (v2.1.2) were used to infer ligand-receptor interactions for regions of interests, within a 250-μm distance. Ligand-receptor pairs of cell-cell contact signals, secreted signals, and ECM receptors included in human CellChatDB (*79*) were used to predict the ligand-receptor pairs. In a*n* analysis on hi-res OSCC sections, each spot was annotated by the top two cell types inferred on the basis of deconvolution to predict the potential senders and receivers of significant communication signals. We further investigated whether a positive signal was also enriched in a group of interest (e.g., pNR after treatment). For each significant ligand-receptor pair *i* in the group *k*, we defined

$${\text{fold enrichment}}_{\mathrm{ik}}=\frac{c_{\mathrm{ik}}/\sum_{j=1}^{K}c_{\mathrm{ij}}}{C_{k}/\sum_{j=1}^{K}C_{j}}$$

Where *c*_*ik*_ and *C*_*k*_ respectively represent the sum of communication frequency/strength of pair *i* and all the significant pairs predicted in group *k*; *K* is the number of groups. We assumed that a ligand-receptor pair is enriched in a specific group if fold enrichment > 1. Significant cell-cell communication pairs in post–pNR-1 were validated by COMMOT on the corresponding Xenium data, in which the cells were annotated by mapping the same single-cell inference dataset (described in Spatial deconvolution and cell–cell colocalization of OSCC) using FindTransferAnchors and TransferData functions in Seurat.

### Construction and validation of the prognostic model

The up-regulated candidate genes were first subjected to univariate Cox regression analysis to assess their association with overall survival in TCGA-HNSC patients, and genes with *P* < 0.05 were selected. To reduce multicollinearity and prevent overfitting, LASSO Cox regression with 10-fold cross-validation was then applied, and genes with nonzero coefficients were retained as core prognostic genes. These genes were subsequently incorporated into a multivariate Cox regression model to derive regression coefficients and construct a risk score. Patients were stratified into high- and low-risk groups based on the median risk score, and survival differences were evaluated using Kaplan-Meier analysis and the log-rank test, with model performance assessed by the concordance index (C-index). To validate the clinical significance of EMT-associated 18 genes, we used the TCGA-HNSC, ESCA, and CESC dataset (*n* = 502) for prognostic analysis (*29*, *30*).

## Supplementary Materials

### The PDF file includes:

Figs. S1 to S29 Legends for tables S1, S4, S5, S7, S9, S10, S12 and S14 Tables S2, S3, S6, S8, S11, S13, S15 to S17 Supplementary methods and Decoder-FFPE-seq protocol Legend for supplementary excel spreadsheet S1 Legend for source data S1

### Other Supplementary Material for this manuscript includes the following:

Tables S1, S4, S5, S7, S9, S10, S12 and S14

Supplementary Excel Spreadsheet S1
