# Supplementary material for: Decoder-FFPE-seq enables sensitive, genome-wide spatial transcriptomics of archival tissues at single-cell resolution
Source: Sci Adv. 2026 Oct 30;12(44):eady5085. doi: 10.1126/sciadv.ady5085 (PMC13721974; doi:10.1126/sciadv.ady5085)
Supplement: Supplementary file 1 [file sciadv.ady5085_sm.pdf]

Science Advances
AAAS

Supplementary Materials for

Decoder-FFPE-seq enables sensitive, genome-wide spatial transcriptomics of archival tissues at single-cell resolution

Jiao Cao *et al.*

Corresponding author: Gang Chen, geraldchan@whu.edu.cn; Lingling Wu, llwu@shsmu.edu.cn; Chaoyong Yang, cyyang@xmu.edu.cn

Sci. Adv. **12**, eady5085 (2026)
DOI: 10.1126/sciadv.ady5085

The PDF file includes:

Figs. S1 to S29
Legends for tables S1, S4, S5, S7, S9, S10, S12 and S14
Tables S2, S3, S6, S8, S11, S13, S15 to S17
Supplementary methods and Decoder-FFPE-seq protocol
Legend for supplementary excel spreadsheet S1
Legend for source data S1

Other Supplementary Material for this manuscript includes the following:

Tables S1, S4, S5, S7, S9, S10, S12 and S14
Supplementary excel spreadsheet S1
Source Data S1

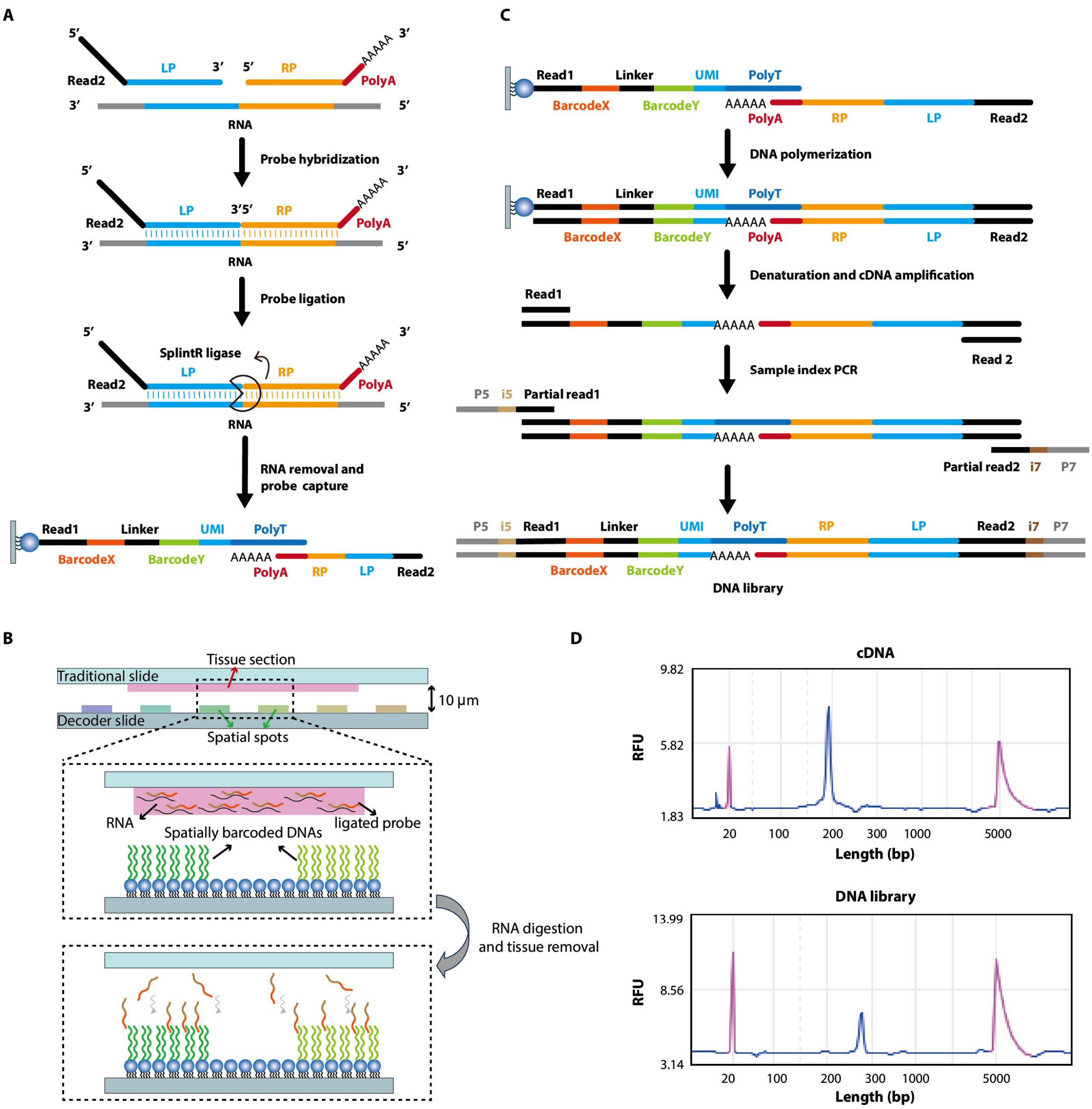

Fig. S1. Detailed workflow of Decoder-FFPE-seq. (A) Probe hybridization, ligation, and release for blotting-assisted spatial barcoding. **(B)** Schematic diagram of blotting-assisted spatial barcoding. **(C)** DNA polymerization, cDNA amplification, and sample index PCR for DNA sequencing library preparation. **(D)** Distribution of cDNA library sizes (top) and DNA sequencing library sizes (bottom).

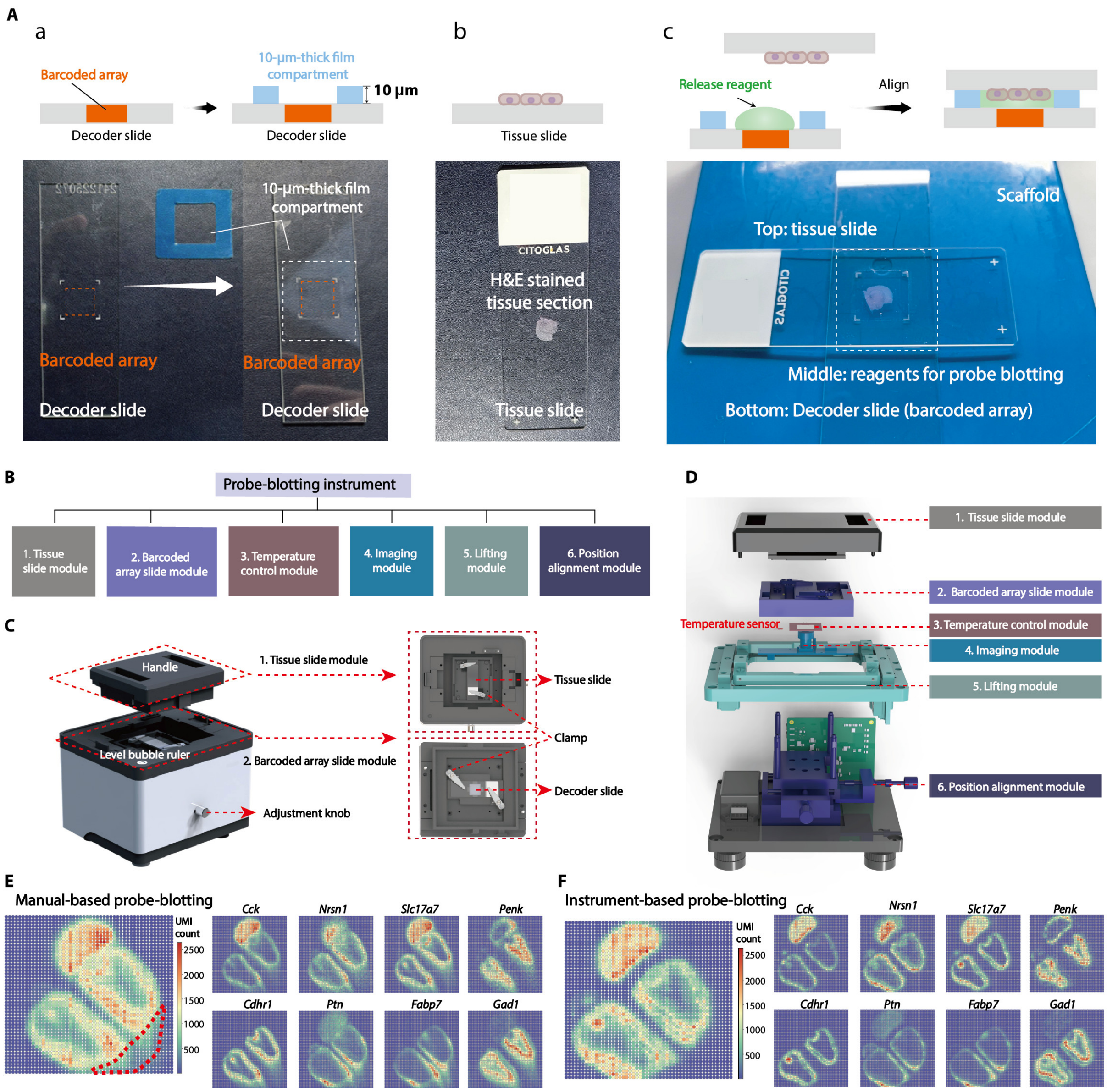

Fig. S2. Manual and instrument-based probe-blotting. (A) Schematic diagram (top) and representative image (bottom) of 10-μm-film compartment and Decoder slides (a), tissue slide (b), and manual probe-blotting c). (B) Design concept of the probe-blotting instrument. (C) Overall appearance of the probe-blotting instrument (left) and its tissue slide / barcode slide module (right). (D) Exploded view of the probe-blotting instrument. (**E-F**) UMI map (left) and marker gene distribution (right) of mouse olfactory bulb (MOB) from manual-based probe-blotting (**E**) and instrument-based probe-blotting (**F**).

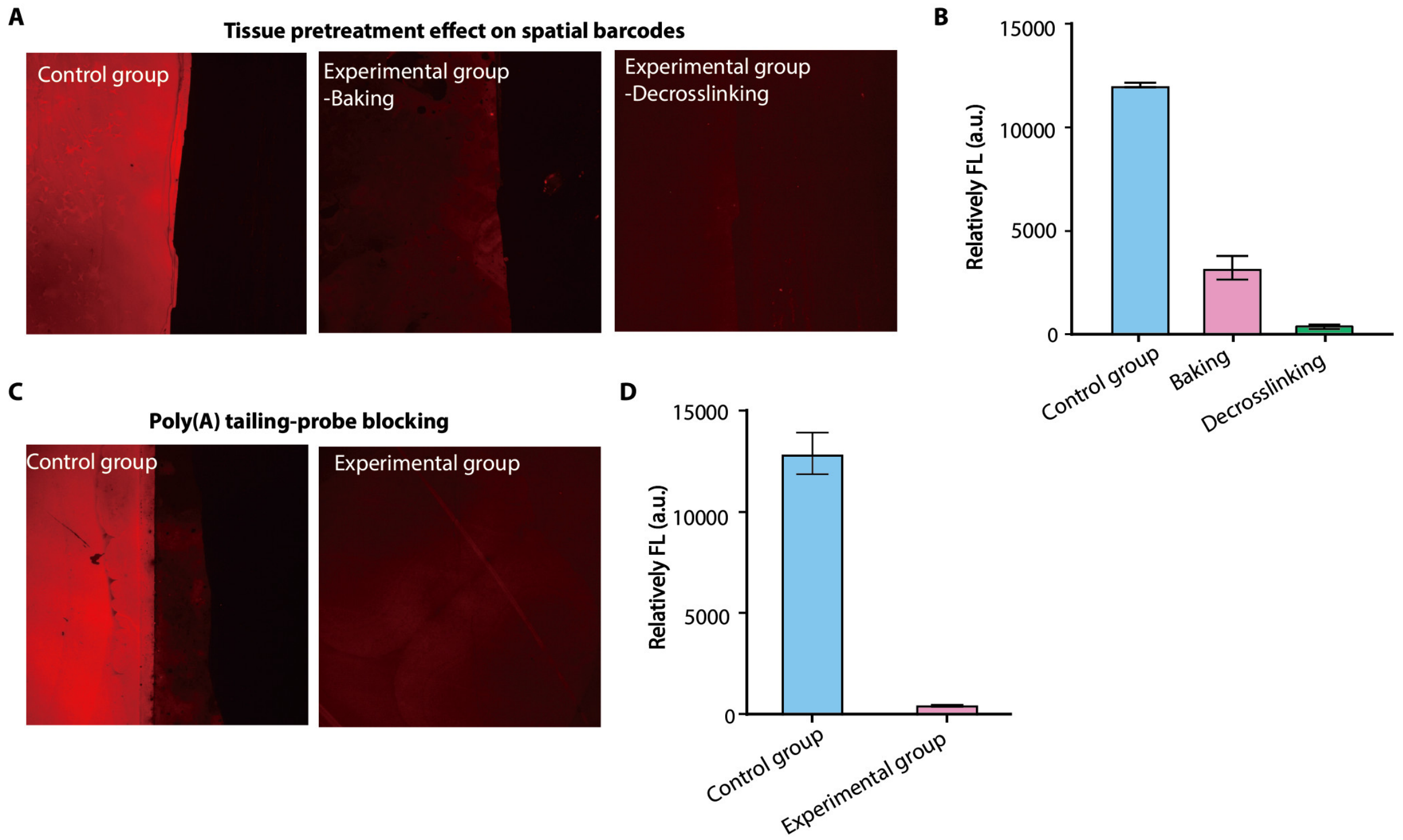

Fig. S3. Fluorescence characterization of tissue pretreatment effect and poly(A) tailing-probe blocking on barcoded DNA. (A) Left: fluorescence image of a Decoder slide hybridized with the Cy3-labeled poly(A) DNA without any treatment. Middle: fluorescence image of a Decoder slide hybridized with the Cy3-labeled poly(A) DNA after being baked at 42°C for 3 h and then 60°C for 2 h. Right: fluorescence image of a Decoder slide hybridized with the Cy3-labeled poly(A) DNA after being treated at 95°C for 1h in the de-crosslinking buffer. **(B)** Relative fluorescence intensity (FL) as quantified from the corresponding images (n =3). **(C)** Left: fluorescence image of a Decoder slide hybridized with the Cy3-labeled polyA DNA without any treatment. Right: fluorescence image of Decoder slides hybridized with the Cy3-labeled polyA DNA after probe labeling on the mouse olfactory bulb (MOB). **(D)** Relative fluorescence intensity (FL) as quantified from the corresponding images (*n* =3). A detailed experimental procedure is described in the Methods.

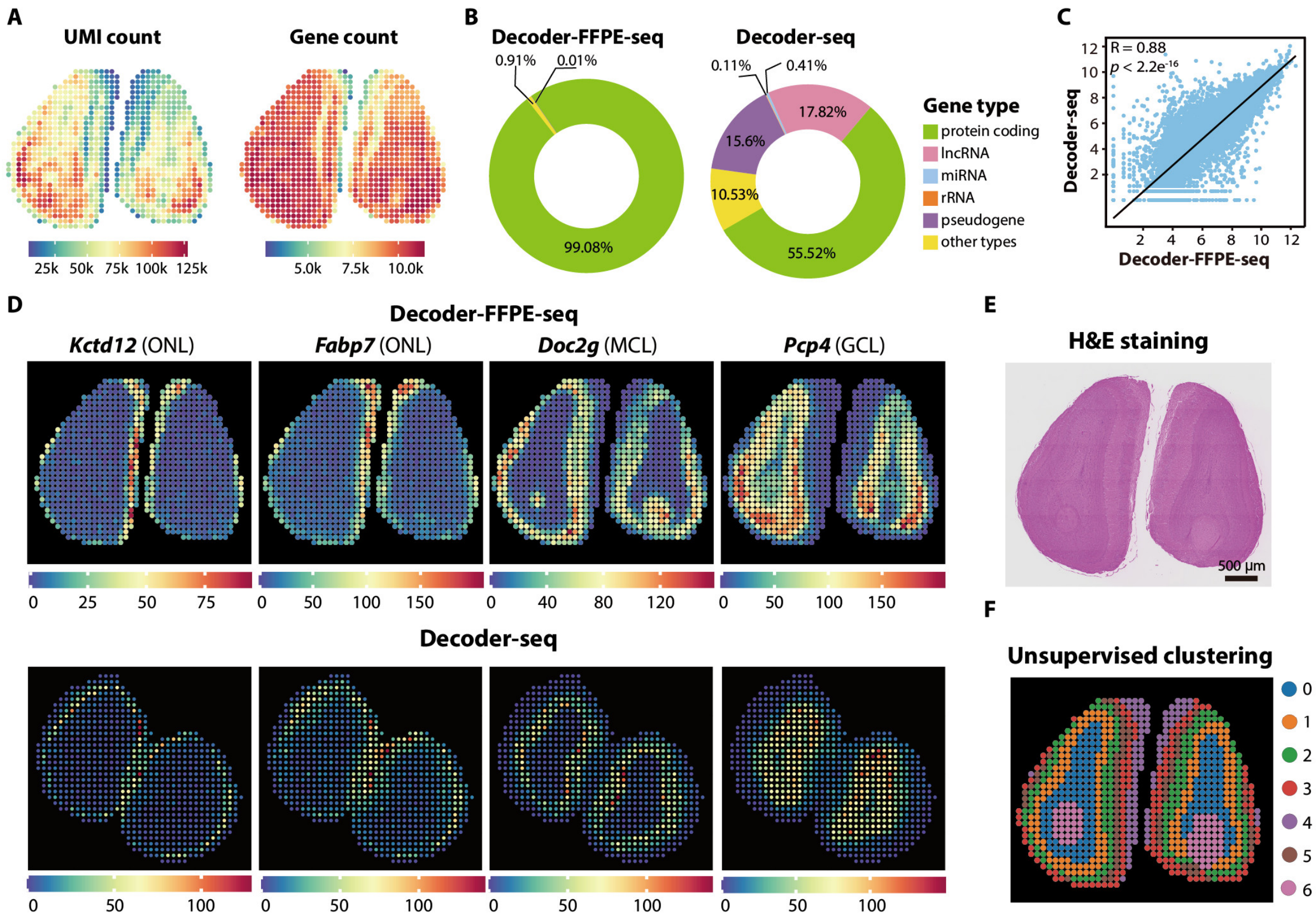

Fig. S4. Quality evaluation of MOB data from Decoder-FFPE-seq and its comparison with Decoder-seq (*21*). (A) Spatial UMI (left) and gene (right) count maps of the MOB from 50 µm-spot Decoder-FFPE-seq. **(B)** Comparison of RNA biotypes captured by Decoder-FFPE-seq and Decoder-seq from MOB samples. **(C)** Gene-gene scatter plots between Decoder-FFPE-seq data (x-axis) and Decoder-seq data (y-axis) of log1p-transformed UMI counts. **(D)** Spatial visualization of layer marker genes of MOB in Decoder-FFPE-seq and Decoder-seq. **(E)** Hematoxylin & Eosin (H&E) staining (top) and unsupervised clustering of the MOB section (bottom) as determined with 50-µm-spot Decoder-FFPE-seq.

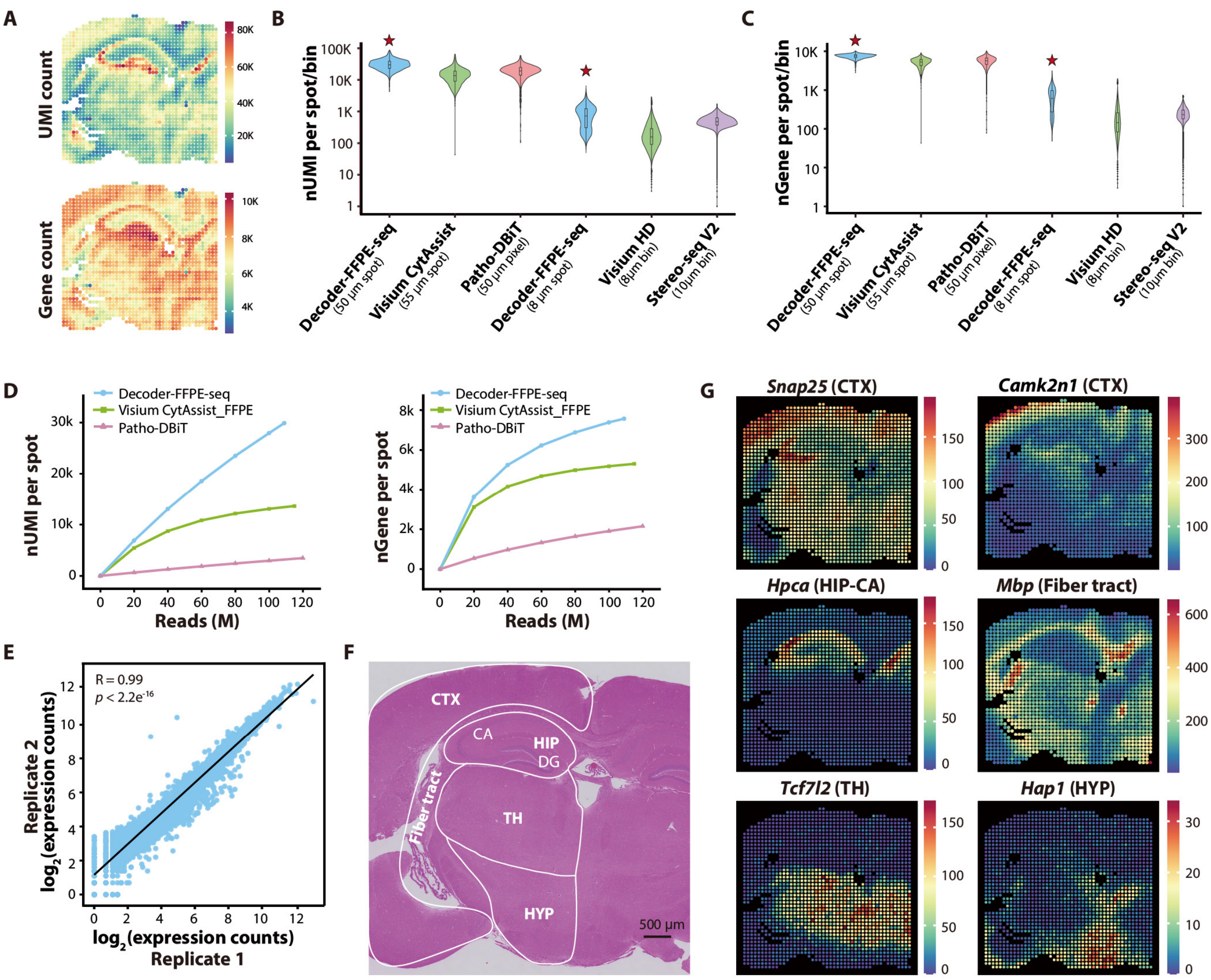

Fig. S5. Mouse brain data from Decoder-FFPE-seq. (A) Spatial UMI (top) and mRNA (bottom) count maps of the mouse brain from 50 µm-spot Decoder-FFPE-seq. **(B-C)** Distribution of UMI counts (**B**) and gene counts (**C**) per spot of FFPE mouse brains from Decoder-FFPE-seq vs other FFPE-compatible spatial transcriptomics (ST) platforms (Visium CytAssist_FFPE, Patho-DBiT, Stereo-seq V2, Visium HD). In Patho-DBiT, only mRNA counts were used. Red stars highlight the performance of Decoder-FFPE-seq. nUMI: number of UMI; nGene: number of gene. **(D)** Saturation curves of median nUMI (left) and nGene (right) per spot from 50-µm spot Decoder-FFPE-seq, 55-µm-spot Visium CytAssist_FFPE, and 50-µm-spot Patho-DBiT. **(E)** Gene consistency of results generated from two replicate brain sections (R = 0.99). **(F)** Hematoxylin & Eosin (H&E) staining of the brain section. CTX: cortex**;** HIP: hippocampus**;** TH: thalamus; HYP: hypothalamus. **(G)** Spatial visualization of regional marker genes of mouse brain in Decoder-FFPE-seq.

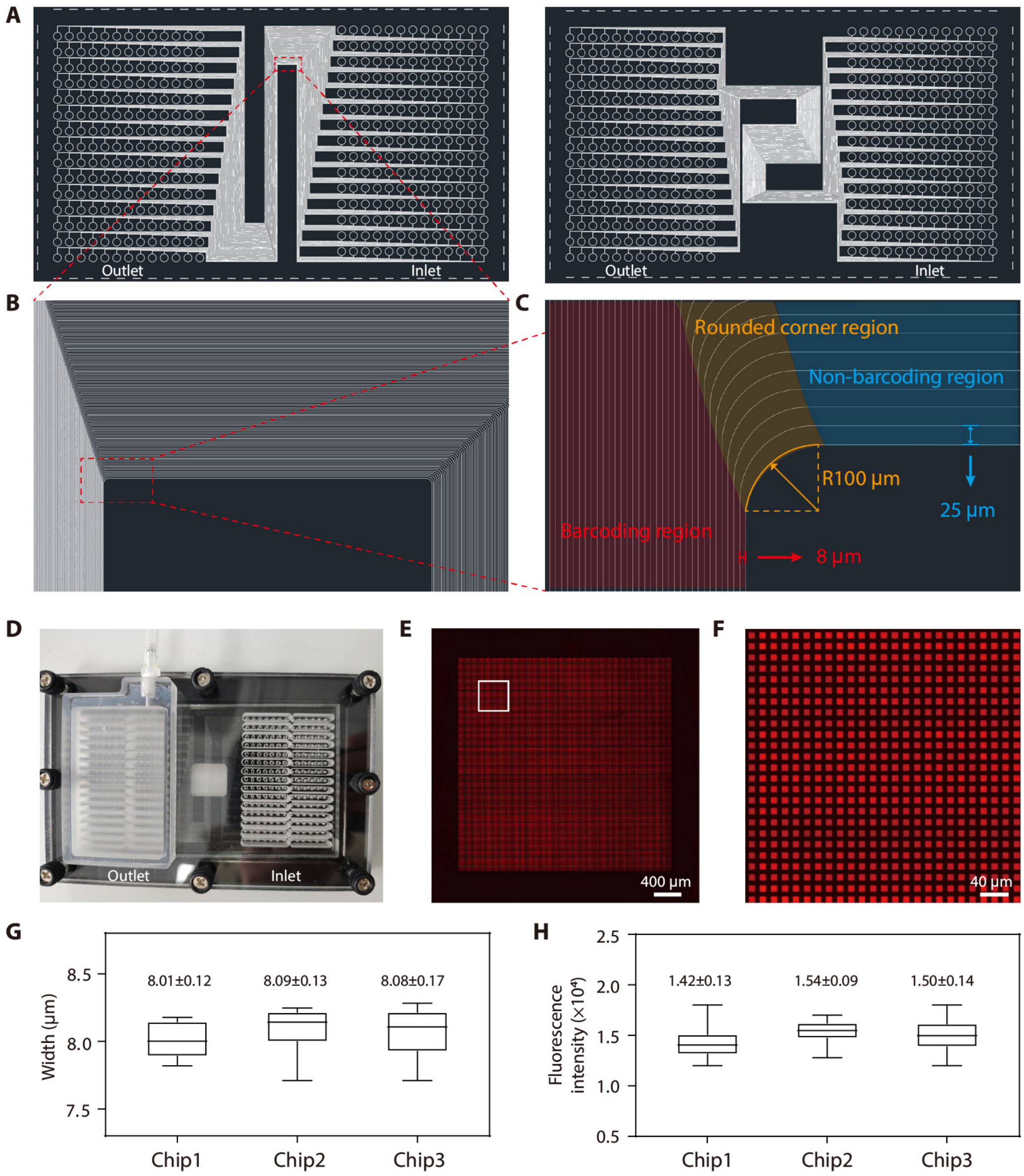

Fig. S6. Microfluidic device design and 8 μm-spot Decoder slides characterization. (A) AutoCAD representations of microchannel chips with barcoding channel width from 8 μm and channel count of 192. **(B)** Detailed AutoCAD view of the region outlined by the red dashed line in **(A)**. **(C)** Detailed AutoCAD view of the region outlined by the red dashed line in (B), showing rounded corners (radius=100 μm) and variable channel widths, transitioning from 25 μm in non-barcoding regions to 8 μm at the barcoding regions. **(D)** Microfluidic device for 8 μm-spot Decoder-FFPE-seq. A PDMS chip mounted on a slide and clamped with acrylic plates, where barcode reagents were loaded into inlets and drawn through microchannels by negative pressure applied at the outlets. **(E)** Global fluorescence image showing polyA-Cy3 hybridized to the terminal polyT sequences of spatial barcodes. **(F)** Magnified view of the boxed region in **(E)**. **(G-H)** Spot size **(G)** and fluorescence intensity **(H)** quantification of 8 μm spots. Based on fluorescence-based quality

assessment, the fabrication success rate of 8 μm-spot Decoder slides is approximately 60%. The fabrication throughput was estimated to be 2-3 slides per person per day.

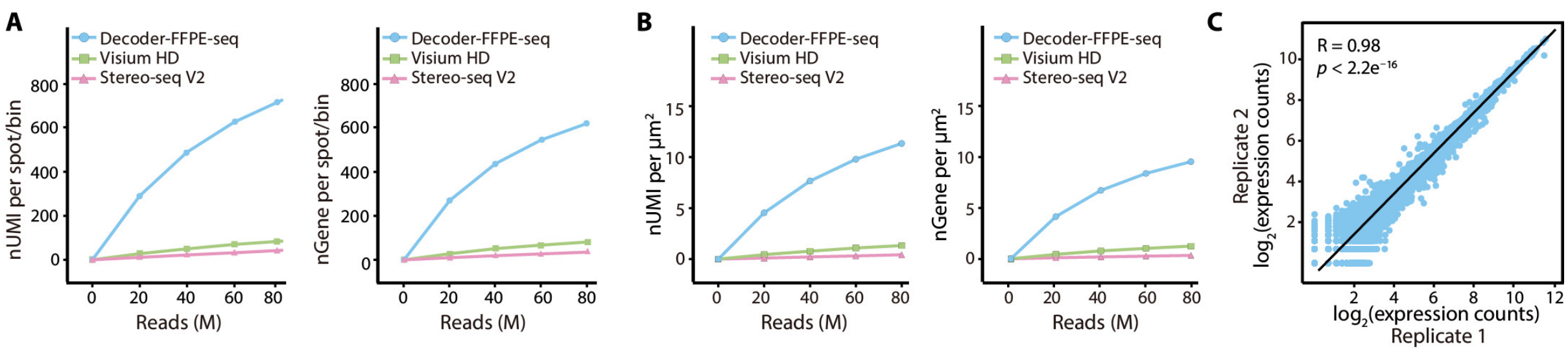

Fig. S7. Quality evaluation of mouse brain data 8 µm-spot Decoder-FFPE-seq. (A-B) Saturation curves of median nUMI (left) and nGene (right) per spot/bin (**A**) and per um^2 (**B**) from 8 µm-spot Decoder-FFPE-seq, 10 µm-bin Stereo-seq V2 (bin20), and 8 µm-bin Visium HD. **(C)** Gene consistency of 8 µm-spot Decoder-FFPE-seq results generated from two replicate brain sections (R = 0.98).

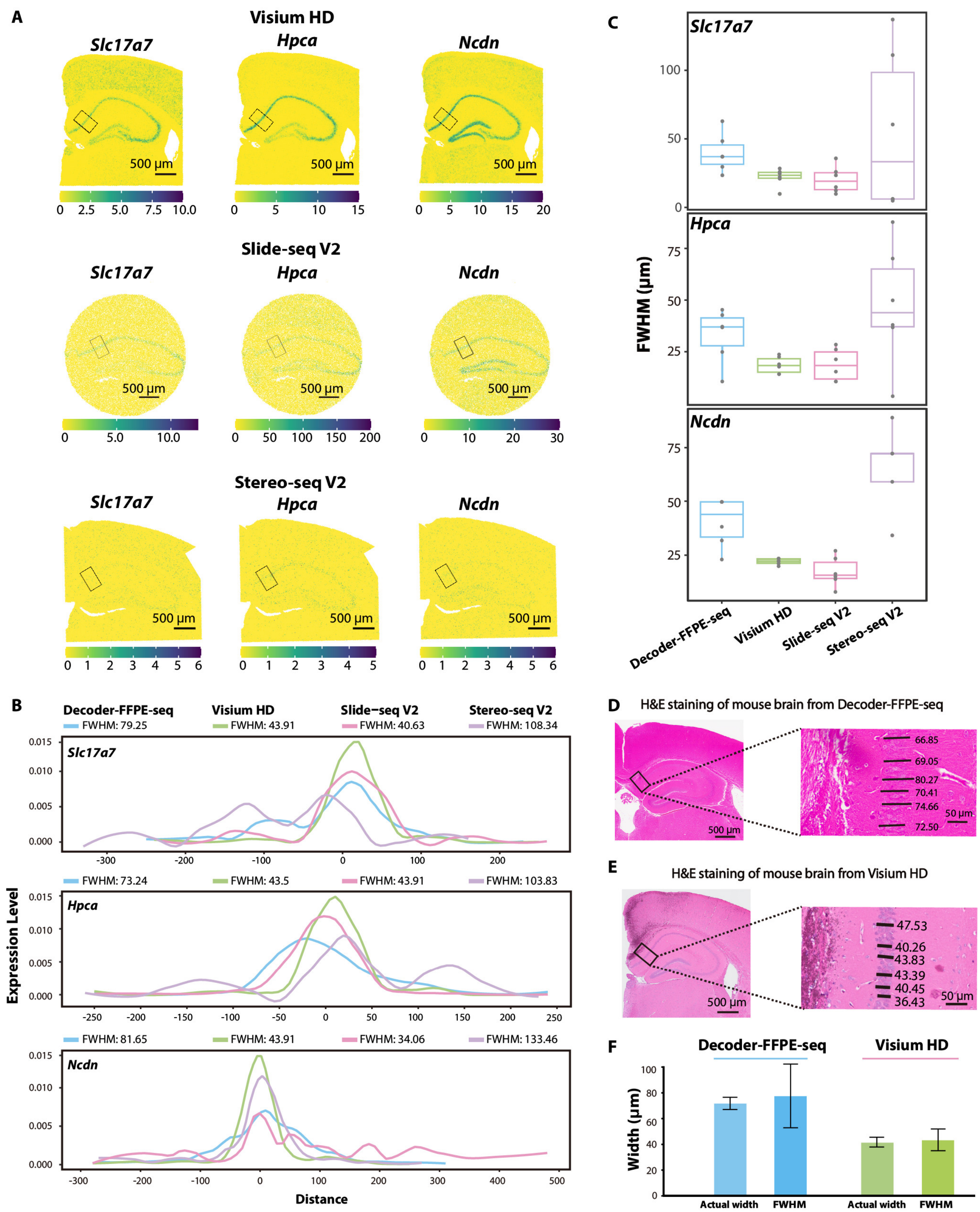

Fig. S8. Comparison of RNA lateral diffusion across ST platforms. (A) Spatial expression of markers in the mouse hippocampus from Visium HD, Slide-seq V2, and Stereo-seq V2. The plots were based on raw counts and black boxes indicated the selected regions used for full-width at full-maximum (FWHM) calculation. (**B**) Expression levels of markers (from **Fig. 1F** and **A**) in mouse brain. UMI counts are averaged across modalities, normalized for each platform and presented in a density plot with the area under the curve set to 1 (details in Methods). (**C**) The FWHM of the profile was then calculated for each gene (from **c**) in

each modality and displayed in boxplots. Each dot represents the FWHM for a given modality (*n* = 6). **(D)** H&E staining of mouse brain from Decoder-FFPE-seq (left) and the actual width of the selected region. The H&E image is the same as that shown in **Fig. 1E**. **(E)** H&E staining of mouse brain from Visium HD (left) and the actual width of the selected region. **(F)** Comparison of actual CA1 width *vs* FWHM of marker gene expression from Decoder-FFPE-seq and Visium HD. FWHM was the average of 18 regions across 3 genes. We observed that tissue section discrepancies may lead to slight variations in FWHM. The CA1 width in Visium HD was narrower than that in Decoder-FFPE-seq, resulting in smaller values.

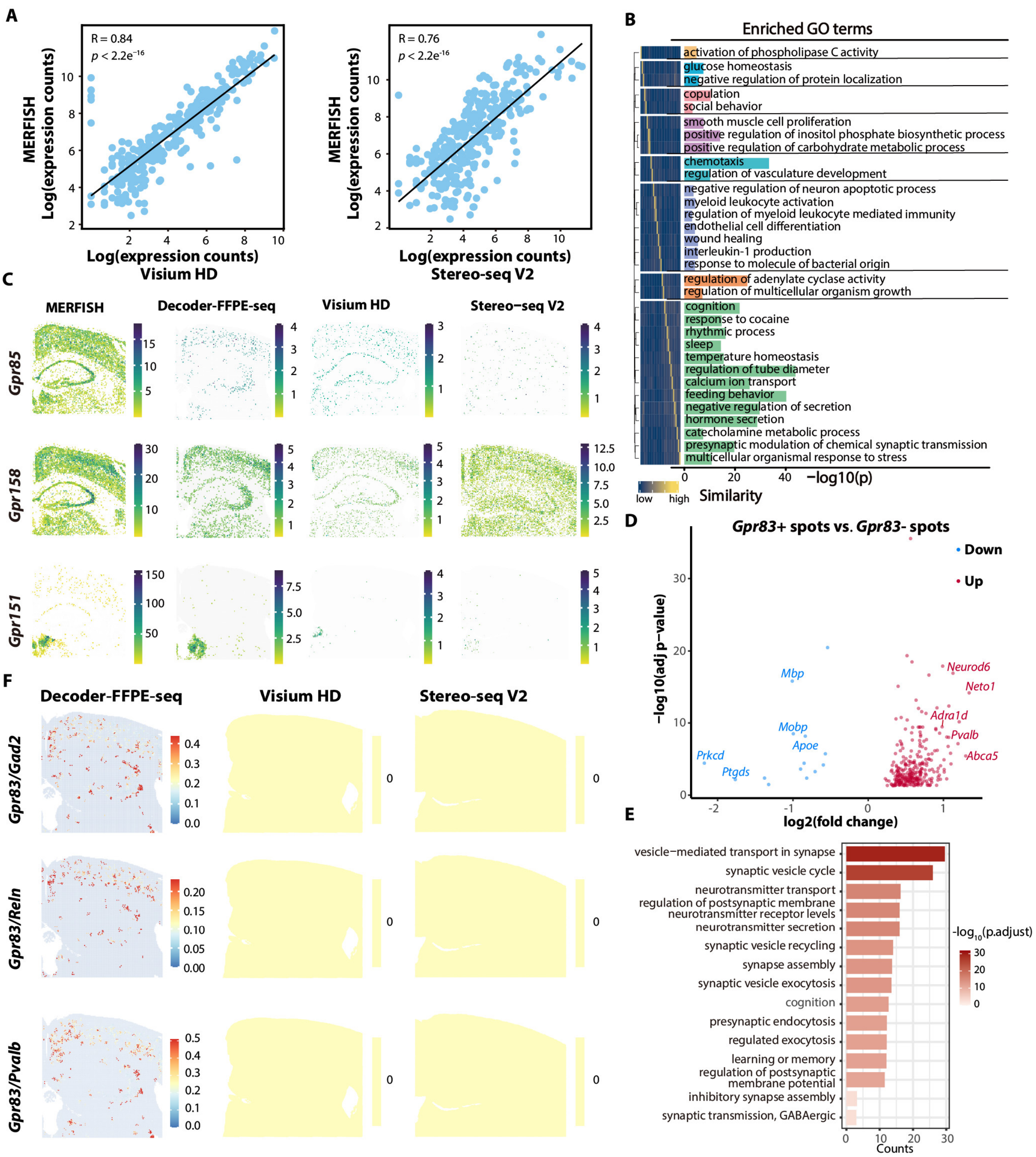

Fig. S9. Spatial profiling of non-sensory G protein-coupled receptors (GPCRs) in the mouse brain. (A) Correlation of GPCR expression between Visium HD (left, R = 0.84) / Stereo-seq V2 (right, R = 0.76) and MERFISH data. **(B)** Function of non-sensory GPCRs annotated by Gene Ontology (GO) terms (right), simplified and clustered by their semantic similarities (left). **(C)** Spatial distribution of representative GPCRs (*Gpr151*, *Gpr85*, *Gpr158*) across ST platforms. **(D)** Volcano plot showing differentially expressed genes (DEGs) between *Gpr83*-positive and *Gpr83*-negative spots. Upregulated genes (red); downregulated genes (blue). Selected representative genes are labeled. **(E)** GO enrichment analysis of upregulated genes in Gpr83-positive spots. **(F)** Spatial co-localization of Gpr83 with *Gad1*, *Reln,* and *Pvalb* across ST platforms.

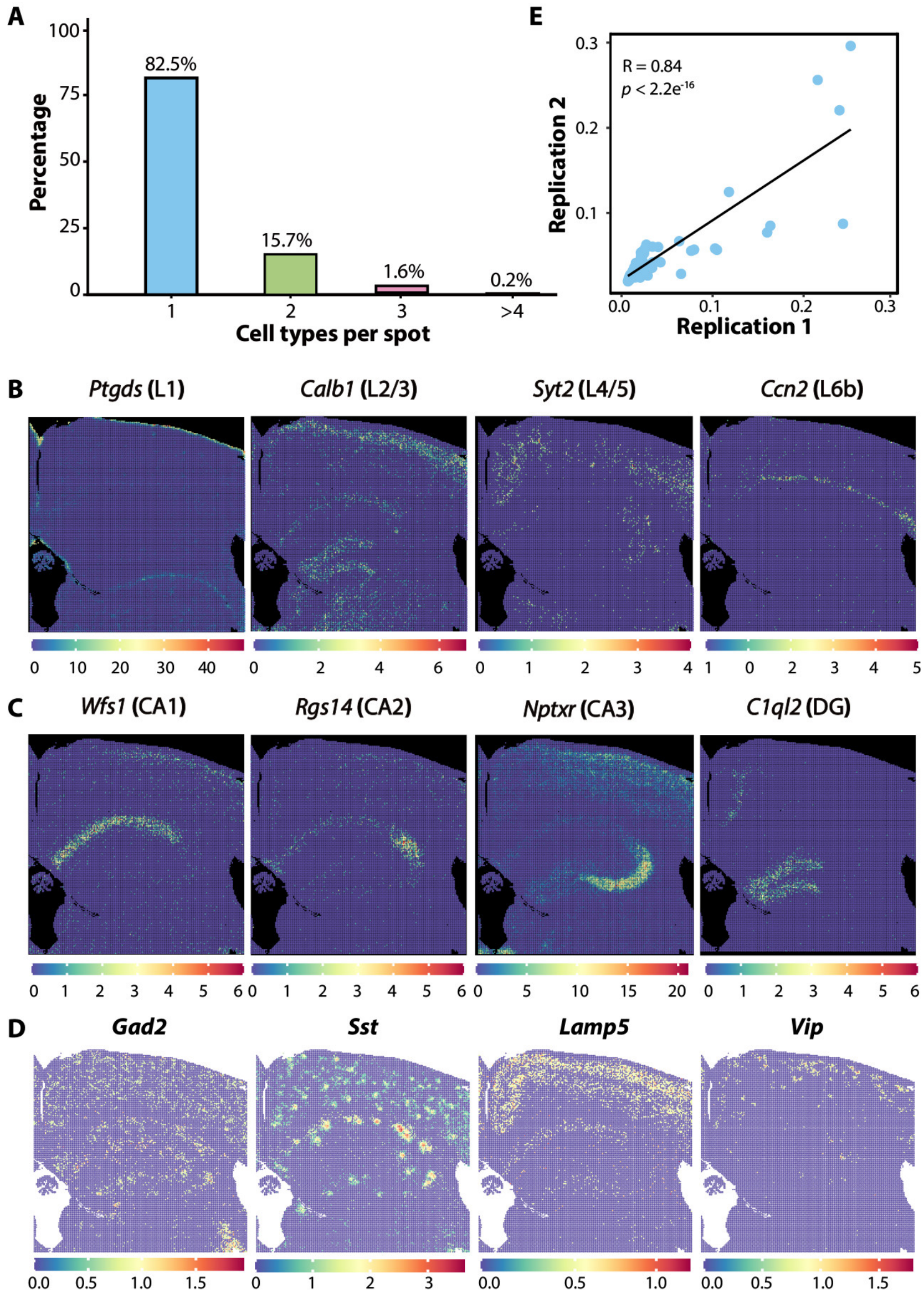

Fig. S10. Spatial gene expression patterns of region-specific marker genes in the mouse brain. (A) The proportion of the numbers of cell types assigned per 8 µm spot. **(B)** Spatial expression of region-specific marker genes of cortex layer as determined with 8 µm-spot Decoder-FFPE-seq. The marker genes and corresponding regions were *Ptgds* (L1), *Calb1* (L2/3), *Syt2* (L4/5), and *Ccn2* (L6b). **(C)** Spatial expression of region-specific marker genes of hippocampal division as determined with 8 µm-spot Decoder-FFPE-seq. The marker genes and corresponding regions were *Wfs1* (CA1), *Rgs14* (CA2), *Nptxr* (CA3), and *Clql2* (DG). CA1/2/3: cornu ammonis 1/2/3; DG: dentate gyrus. **(D)** Spatial expression of marker genes for rare inhibitory neurons (*Gad2*) and their subtypes (*Sst*, *Lamp5*, *Vip*). **(E)** Consistency of cell-type abundance between replicate brain sections profiled by 8 µm-spot Decoder-FFPE-seq ($R = 0.84$).

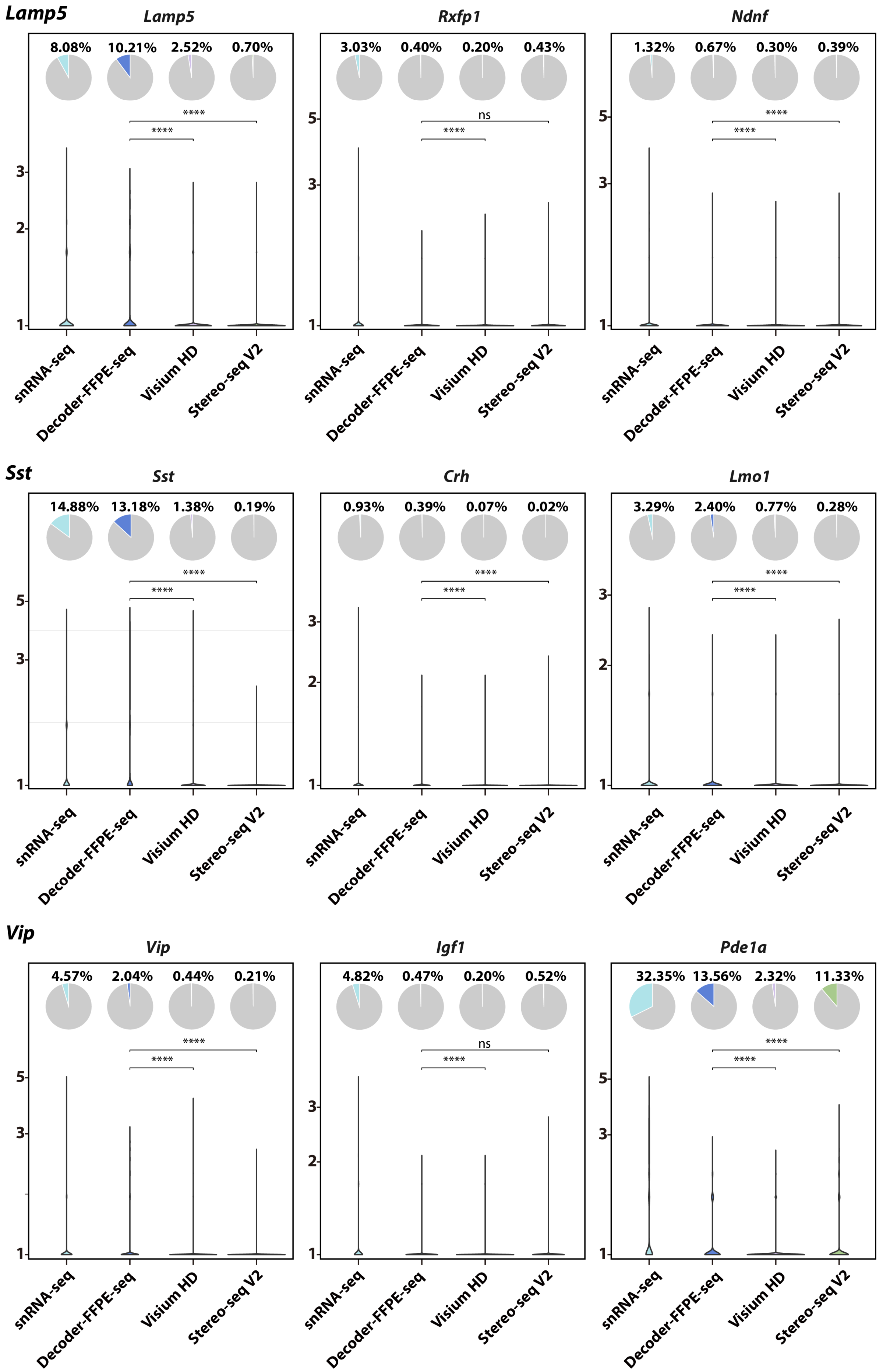

Lamp5
Lamp5
8.08% 10.21% 2.52% 0.70%
Rxfp1
3.03% 0.40% 0.20% 0.43%
ns
Ndnf
1.32% 0.67% 0.30% 0.39%
Sst
Sst
14.88% 13.18% 1.38% 0.19%
Crh
0.93% 0.39% 0.07% 0.02%
Lmo1
3.29% 2.40% 0.77% 0.28%
Vip
Vip
4.57% 2.04% 0.44% 0.21%
Igf1
4.82% 0.47% 0.20% 0.52%
ns
Pde1a
32.35% 13.56% 2.32% 11.33%
****
snRNA-seq
Decoder-FFPE-seq
Visium HD
Stereo-seq V2

Fig. S11. Comparison of mouse brain data between snRNA-seq, 8 μm-spot Decoder-FFPE-seq, Visium HD, and Stereo-seq V2. Expression (UMI counts) and percentage of cell type-specific markers. Top panel: percentage of cell type-specific markers; Bottom panel: violin plots of gene expression levels. *, $p < 0.05$; **, $p < 0.01$; ***, $p < 0.001$; ****, $p<0.0001$.

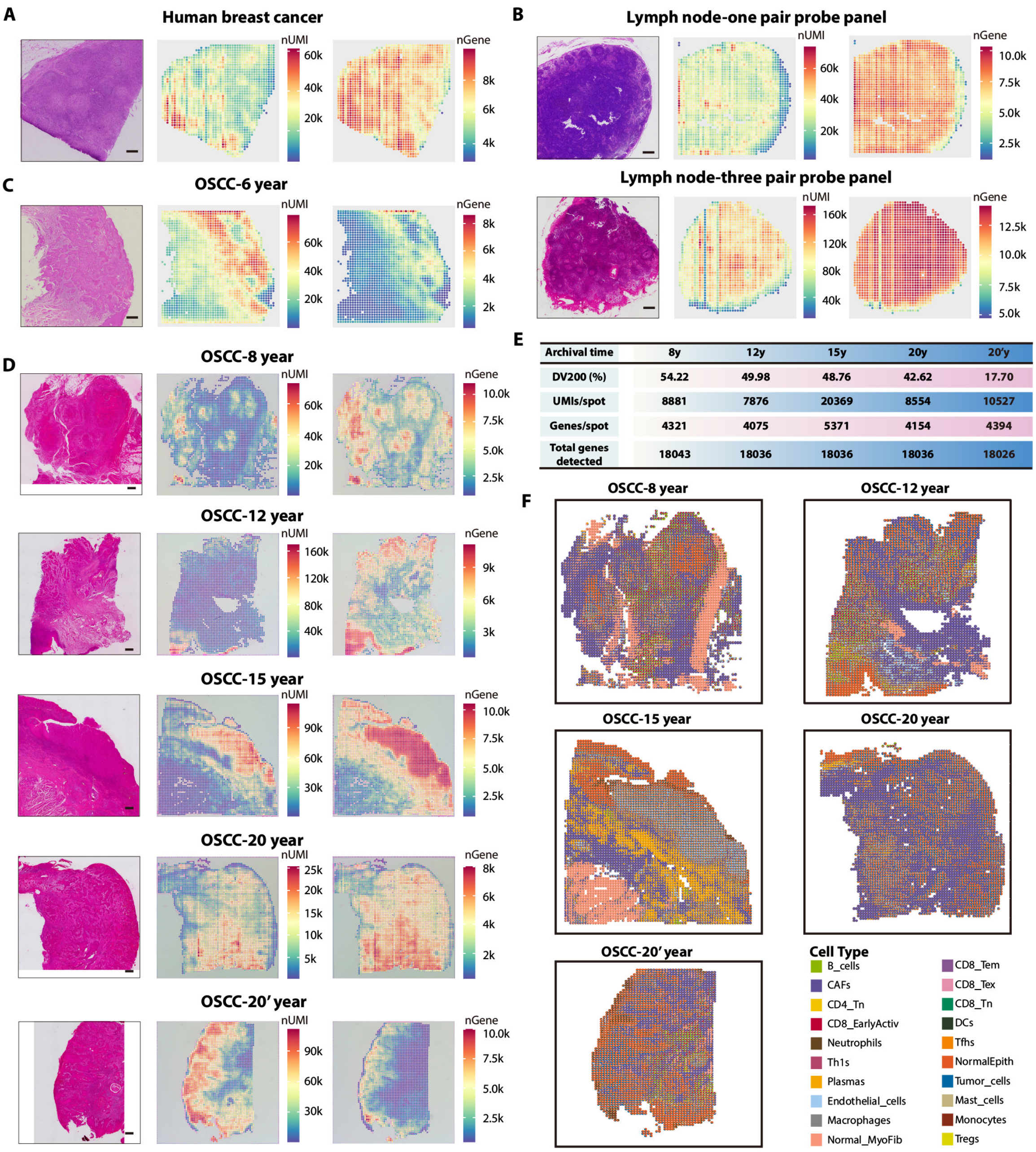

Archival time	8y	12y	15y	20y	20'y
DV200 (%)	54.22	49.98	48.76	42.62	17.70
UMIs/spot	8881	7876	20369	8554	10527
Genes/spot	4321	4075	5371	4154	4394
Total genes detected	18043	18036	18036	18036	18026

Fig. S12. Quality evaluation of archived sample data, related to Fig. 3. (A-D) Left: H&E images of tissue sections; Middle: spatial feature plots showing the nUMI; Right: spatial feature plots showing the nGene. Breast cancer (**A**) and 6-year-old oral squamous cell carcinoma (OSCC) sample (**C**) were profiled using the human one-pair probe panel. Lymph node sections (**B**) were profiled with both the human one-pair and three-pair probe panel. The H&E images of the 8-12-year OSCC samples in (**D**) are the same as that shown in Fig. 3B, and these samples were profiled using the human three-pair probe panel. Scale bar: 500 μm. **(E)** Summary table of archival time, DV200, median gene/UMI counts per spot, and total detected genes for long-term OSCC archives. **(F)** The spatial pie plot of CARD-inferred cell type composition for various

durations OSCC FFPE specimens. CAFs: Cancer-associated fibroblasts; DCs: Dendritic cells; CD4-Tn: Naive CD4 T cells; Tfhs: Follicular helper T cells; Th1s: T helper 1 cells; Tregs: Regulatory T cells; CD8-Tn: Naive CD8 T cells; CD8-EarlyActiv: Early active CD8 T cells; CD8-Tem: Effector memory CD8 T cells: CD8-Tex: Exhaustion CD8 T cells; Normal_MyoFib: Myofibroblasts; NormalEpith: Normal epitheliums.

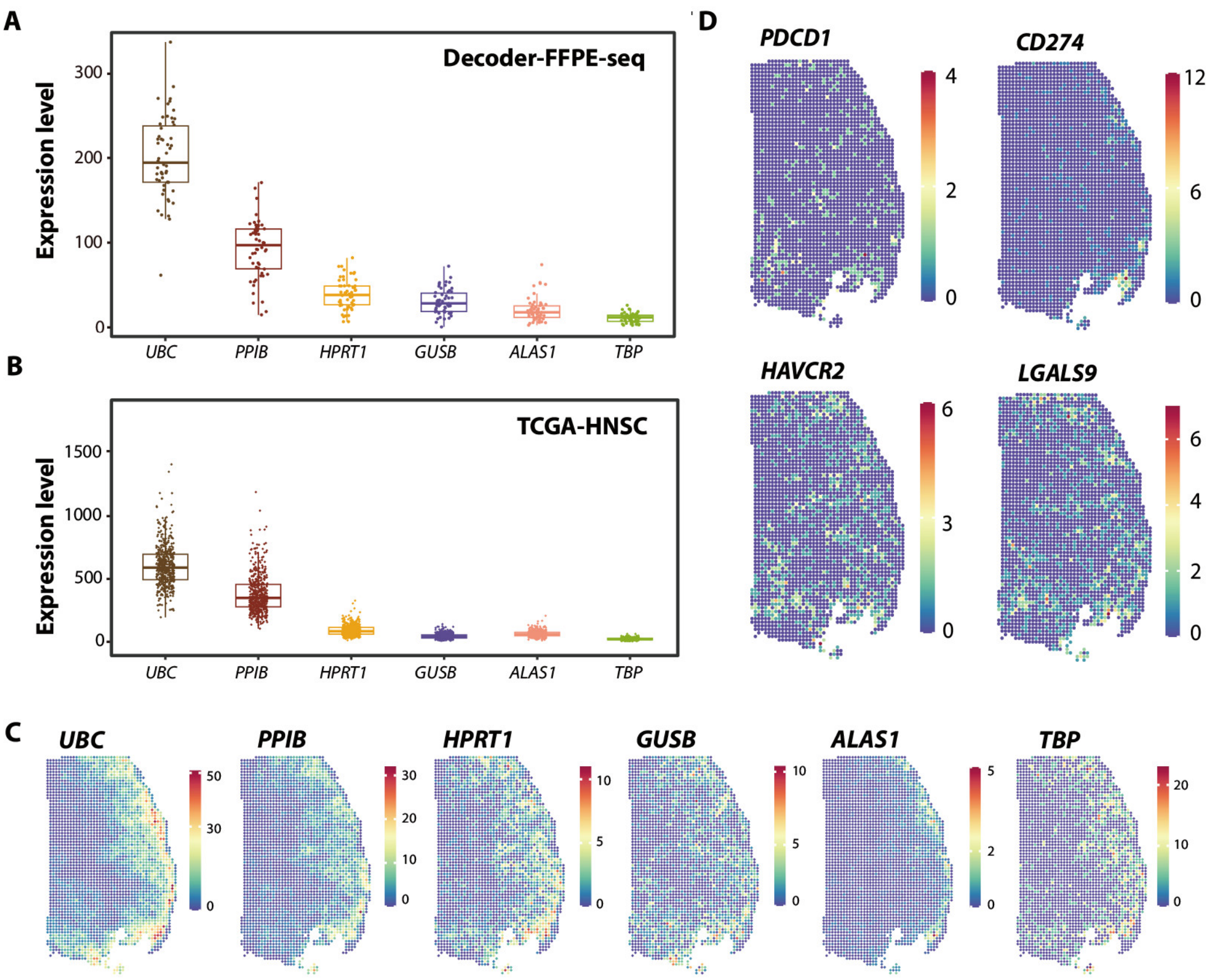

Fig. S13. Performance of Decoder-FFPE-seq in the 20-year-old OSCC sample, related to Fig. 3. (A-B) Expression levels of six housekeeping genes were determined by pseudo-bulk transcript counts of Decoder-FFPE-seq data (**A**) and those derived from bulk RNA-seq data in the TCGA-Head and Neck Squamous Cell Carcinoma (HNSC) cohort (**B**). **(C)** Spatial distribution of housekeeping genes. (**D**) Spatial distributions of receptors and ligands in 20-year-old OSCC section.

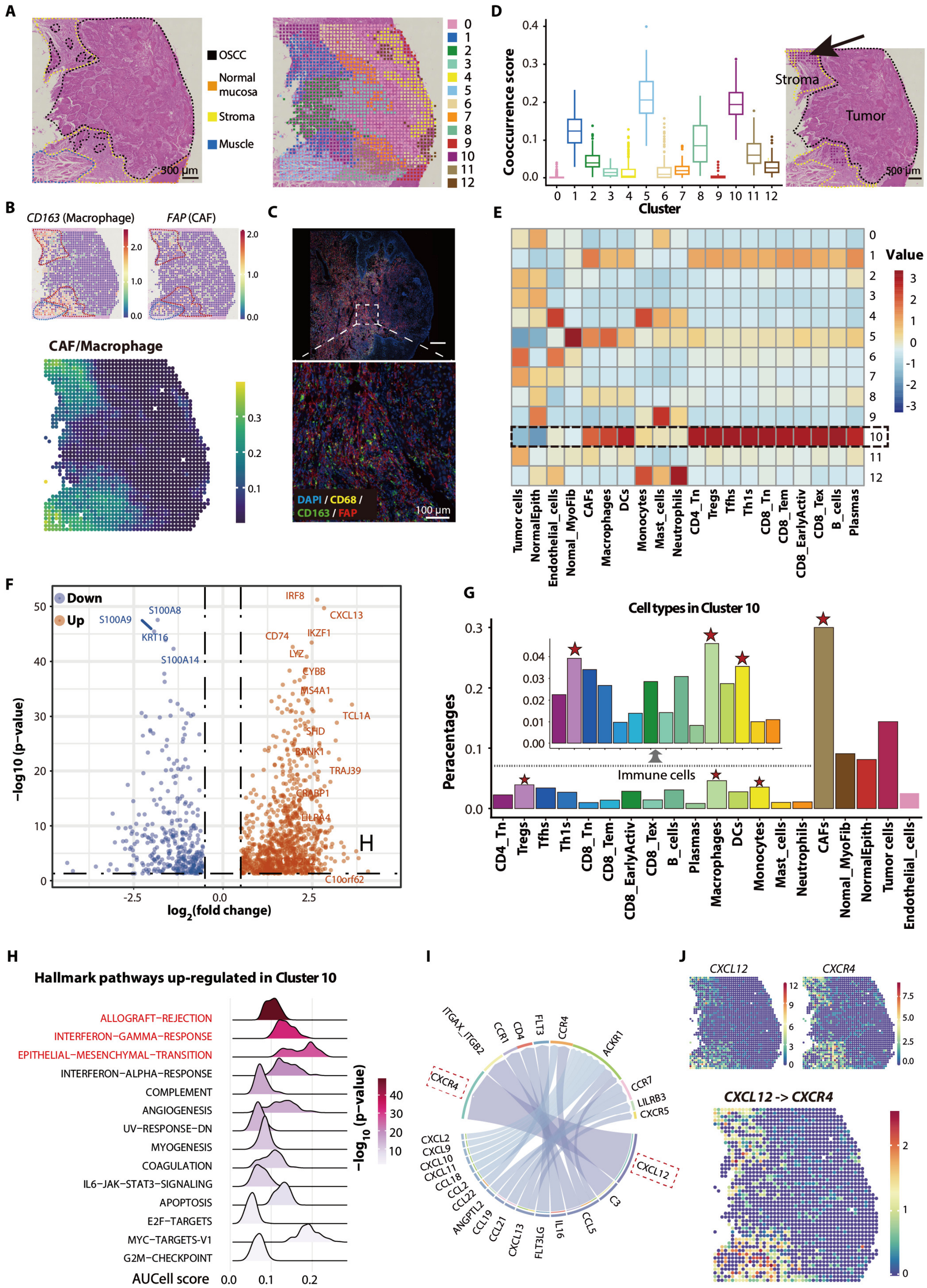

Fig. S14. Decoder-FFPE-seq resolves the tumor microenvironment of the 6-year-old OSCC sample.

(A) H&E image (left) with pathologically annotated regions and unsupervised clustering (right) of the OSCC

section. OSCC (black), stroma (yellow), muscle (light blue), and normal mucosa (orange). **(B)** Co-localization of CAFs and macrophages with marker genes in 6-year-old OSCC section. **(C)** Multiplex immunofluorescence of macrophages (CD68/CD163) and CAFs (FAP) from the same OSCC sample. DAPI (blue), CD68 (yellow), CD163 (green), FAP (red). Bottom: magnified view. **(D)** Cooccurrence score of CAF and macrophage in different clusters. **(E)** Heatmap of cell types among Clusters. **(F)** DEGs in cluster 10 corresponding to the indicated spatial region. **(G-H)** Percentage of cell types **(G)** and upregulated hallmark pathways **(H)** in cluster 10. (I) Spatial distribution and ligand-receptor interactions of cluster 10 in 6-year-old OSCC. (J) Spatial expression of *CXCL12* and *CXCR4* with their interaction intensity in 6-year-old OSCC section.

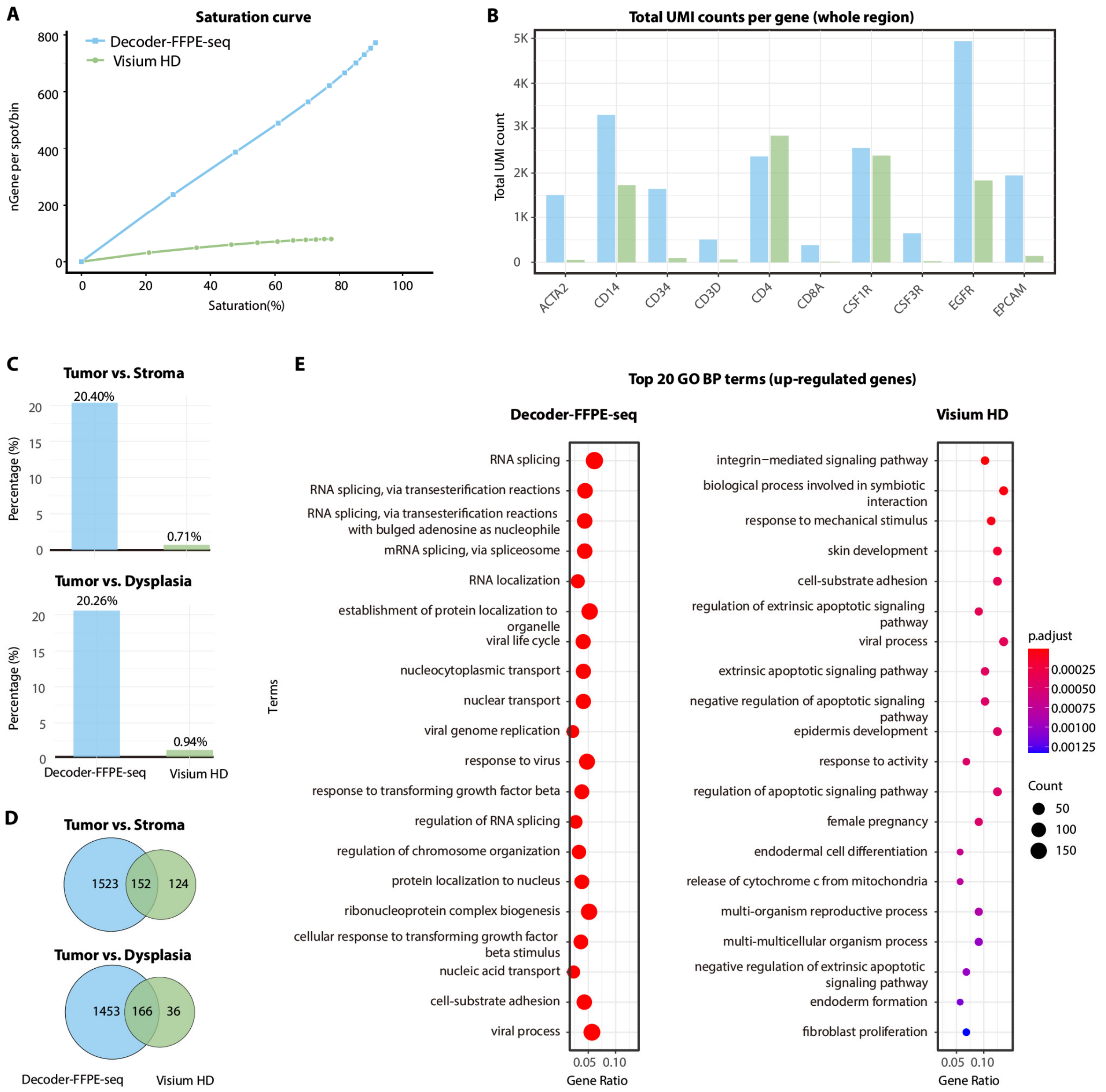

Fig. S15. Comparison of gene detection sensitivity between 8 μm-spot Decoder-FFPE-seq and Visium HD. (A) Comparison of gene counts per spot/bin at matched sequencing saturation. **(B)** Comparison of total UMI counts of marker genes across multiple cell types in the whole tissue region. **(C)** Bar plots representing the proportion of DEGs identified by each platform relative to the total number of detected genes. Comparisons were performed between stromal-invasive tumor and stroma (top) or dysplasia (bottom). **(D)** Venn diagrams showing the overlap of significantly enriched GO biological process (BP) terms between the two platforms for the same two comparisons (top: tumor *vs.* stroma; bottom: tumor *vs.* dysplasia). **(E)** Top 20 enriched GO BP terms from the comparison of stromal invasive tumor *vs.* dysplastic epithelium identified by Decoder FFPE seq and Visium HD.

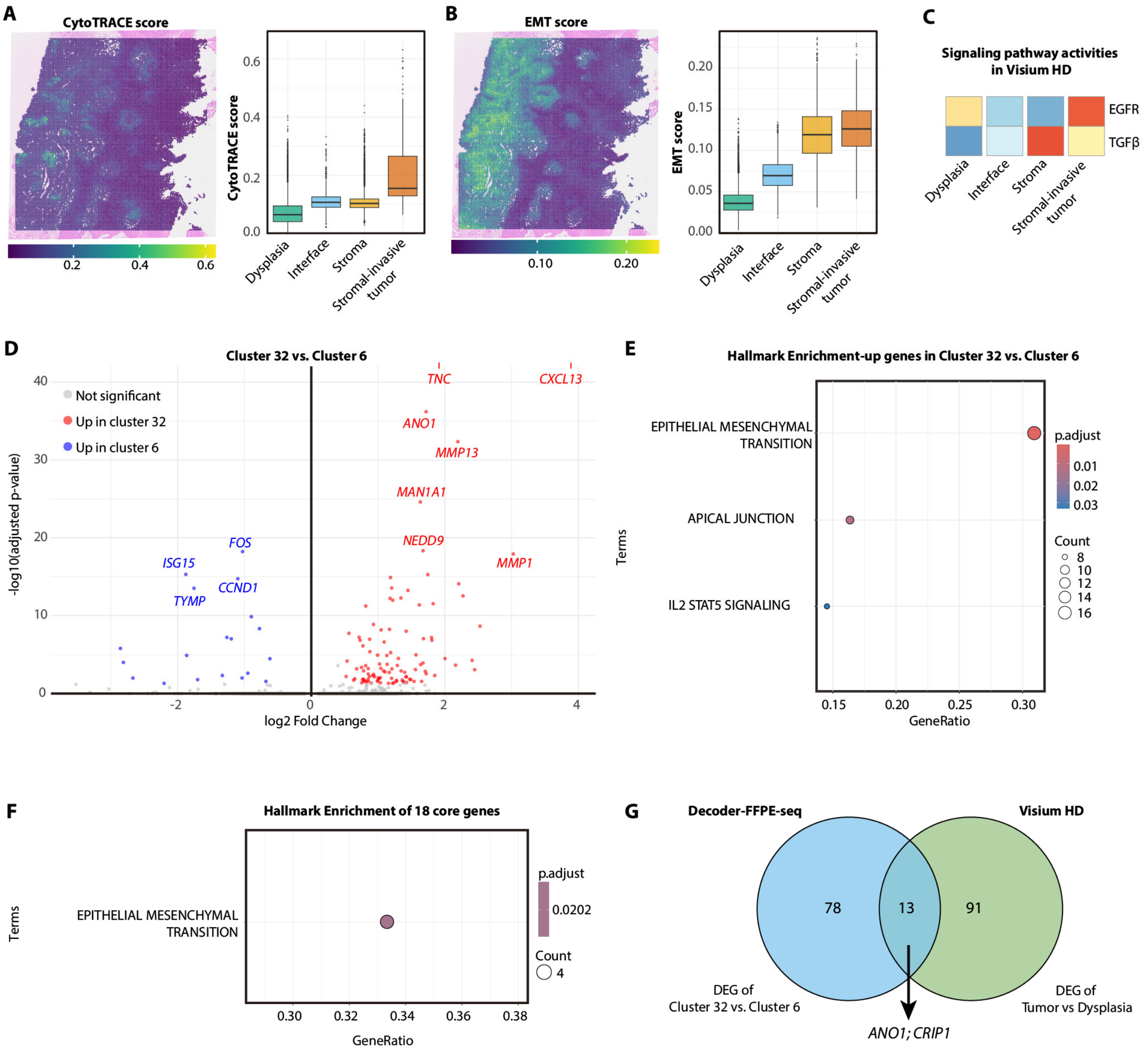

Fig. S16. Decoder-FFPE-seq resolves the boundary region and tumor heterogeneity associated with clinical outcomes. (A) Spatial distribution of CytoTRACE stemness scores (left) and boxplots showing CytoTRACE scores across the four defined regions (dysplastic epithelium, interface (cluster 3), stroma, and stromal-invasive tumor) (right). **(B)** Spatial distribution of EMT pathway scores (AUCell) (left) and boxplots of EMT scores across the four regions (right). **(C)** Standardized mean PROGENy pathway activities along the spatial axis from dysplastic epithelium, through the interface (cluster 10), to stroma, and stromal-invasive tumor, as identified by Visium HD. **(D)** Volcano plot of DEGs between cluster 32 and cluster 6 in Decoder-FFPE-seq. **(E)** Hallmark enrichment analysis of genes upregulated in cluster 32 compared with cluster 6. **(F)** Hallmark enrichment analysis of the 18-gene signature derived from cluster 32. **(G)** Venn diagram showing the overlap of upregulated genes from cluster 32 *vs.* cluster 6 (identified by

Decoder-FFPE-seq) and from stromal-invasive tumor *vs.* dysplastic epithelium (identified by Visium HD). Only two of the 18 genes were shared between the two comparisons.

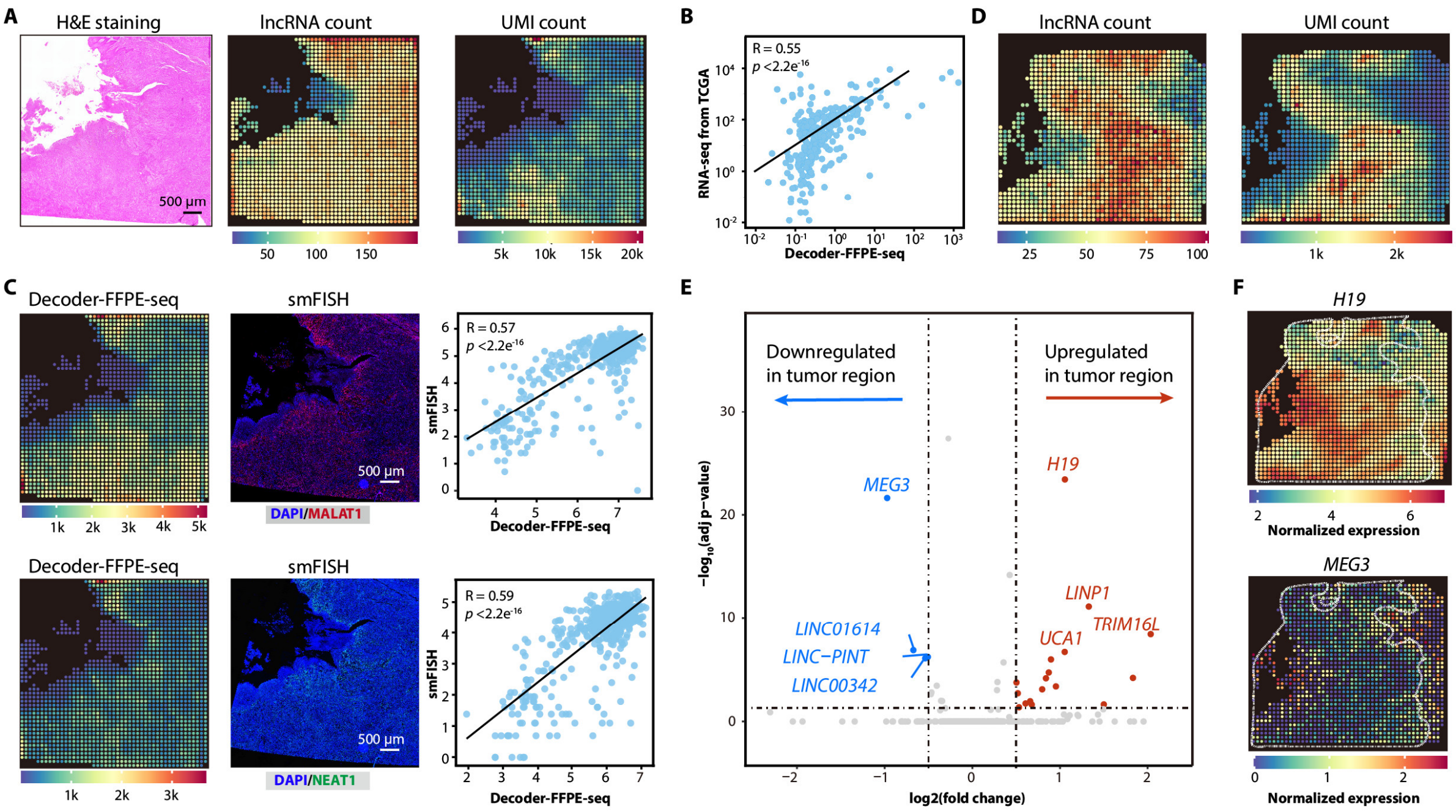

Fig. S17. Spatial co-profiling of mRNA-lncRNA in OSCC archives, related to Fig. 4. (A) H&E image (left), its long non-coding (lncRNA) count (middle), and UMI count (right) maps of the OSCC sample. This sample was profiled with a lncRNA-only probe panel. **(B)** Gene consistency of lncRNA between Decoder-FFPE-seq data and bulk RNA-seq data from the TCGA-HNSC cohort. **(C)** Spatial distribution of *MALAT1* and *NEAT1* in adjacent sections profiled by Decoder-FFPE-seq (left) and smFISH (middle), and their gene expression consistency between the two methods (right). **(D)** lncRNA count (left) and UMI count (right) maps of the OSCC sample profiled with a mRNA-lncRNA probe panel. **(E)** DEGs in tumor region. **(F)** Spatial expression of *H19* and *MEG3*.

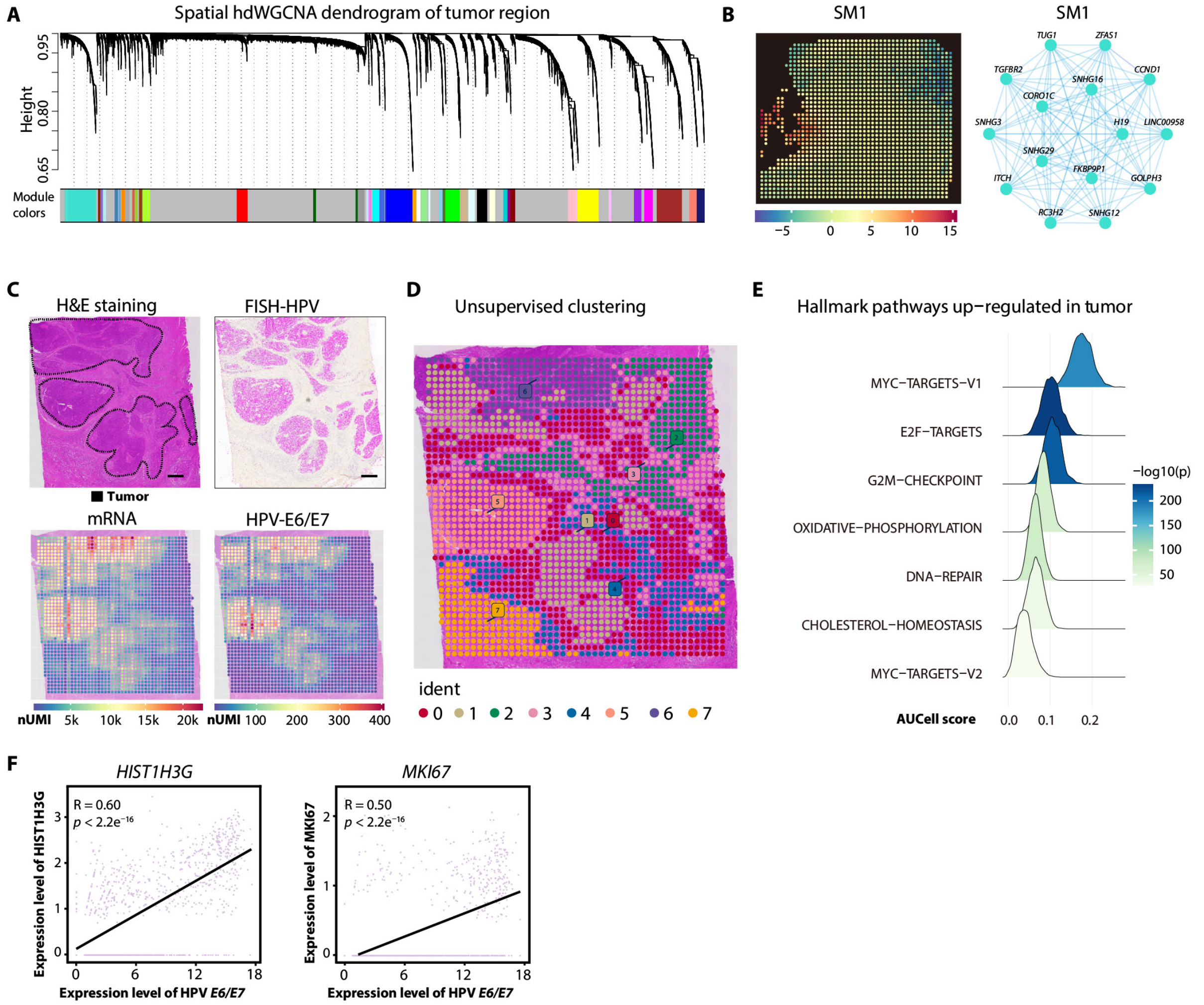

Fig. S18. Spatial co-profiling of mRNA-lncRNA and host mRNA-HPV RNA in OSCC archives, related to Fig. 4. (A) Spatial hdWGCNA modules of tumor region. **(B)** Spatial distributions of SM1 (left) and its co-expression network of the top 15 genes (right). **(C)** Top-left: H&E image of the HPV-infected OSCC sample. Inside the black circle is the tumor tissue, and the rest is the stromal tissue. Top-right: spatial distributions of HPV from FISH. Bottom-left: UMI count map of mRNA in the HPV-positive OSCC section. Bottom-right: UMI count map of HPV16-E6/E7 in the HPV-positive OSCC section. Scale bar: 500 µm. **(D)** Unsupervised clustering of the HPV-positive OSCC section. **(E)** Upregulated hallmark pathways in tumor region of the HPV-positive OSCC section. **(F)** Spatial correlation between E2F downstream genes and HPV expression.

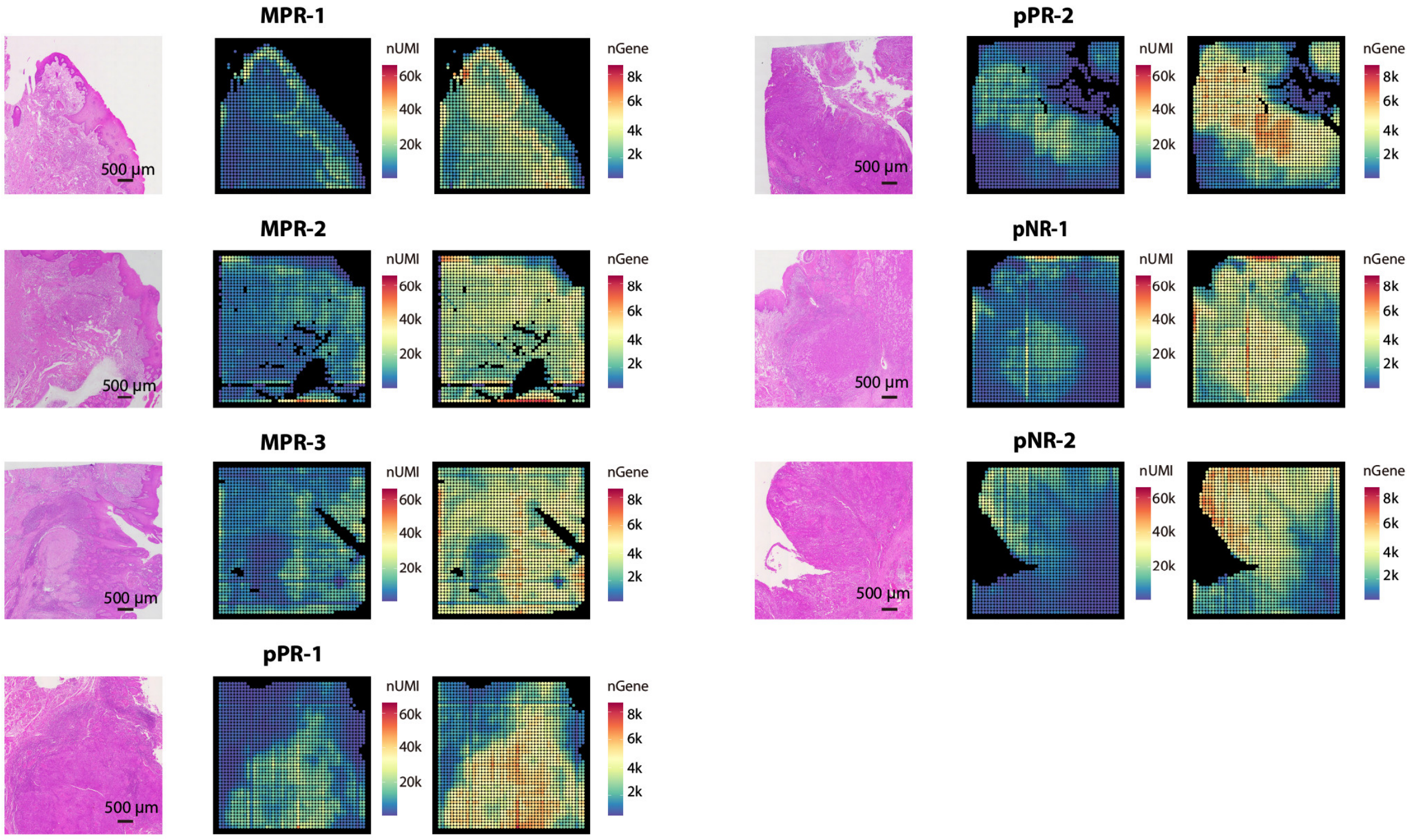

Fig. S19. Assessment of immune checkpoint blockade (ICB) treated OSCC data quality, related to Fig. 5. Columns 1 and 4, H&E-stained tissue sections, identical to those shown in **Fig. 5D**; Columns 2 and 5, spatial feature plots showing the nUMI; Columns 3 and 6, spatial feature plots showing the nGene.

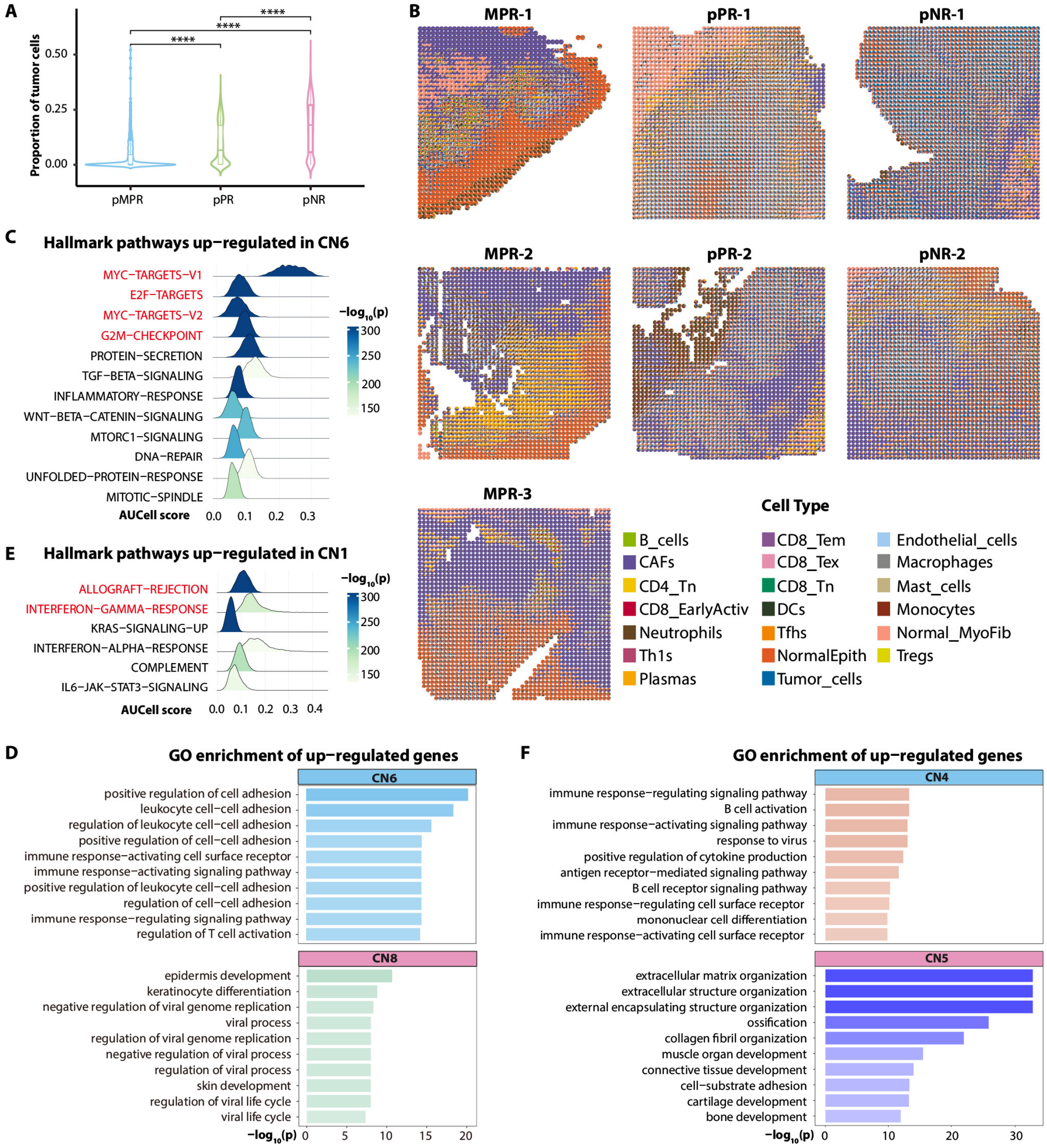

Fig. S20. Decoder-FFPE-seq identifies pathological response-related spatial niches after immunotherapy in OSCC, related to Fig. 5. (A) The proportion of tumor cells among different pathological response groups from neoadjuvant anti-PD-1 Immunotherapy clinical trials. **(B)** The spatial pie plot of CARD-inferred cell type composition for seven post-treatment OSCC FFPE specimens. **(C)** Upregulated hallmark pathways in cell-type niche (CN) 6. **(D)** Gene Ontology (GO) enrichment of DEGs between CN 6 and CN 8. **(E)** Upregulated hallmark pathways in CN 1. **(F)** GO enrichment of DEGs between CN 4 and CN 5.

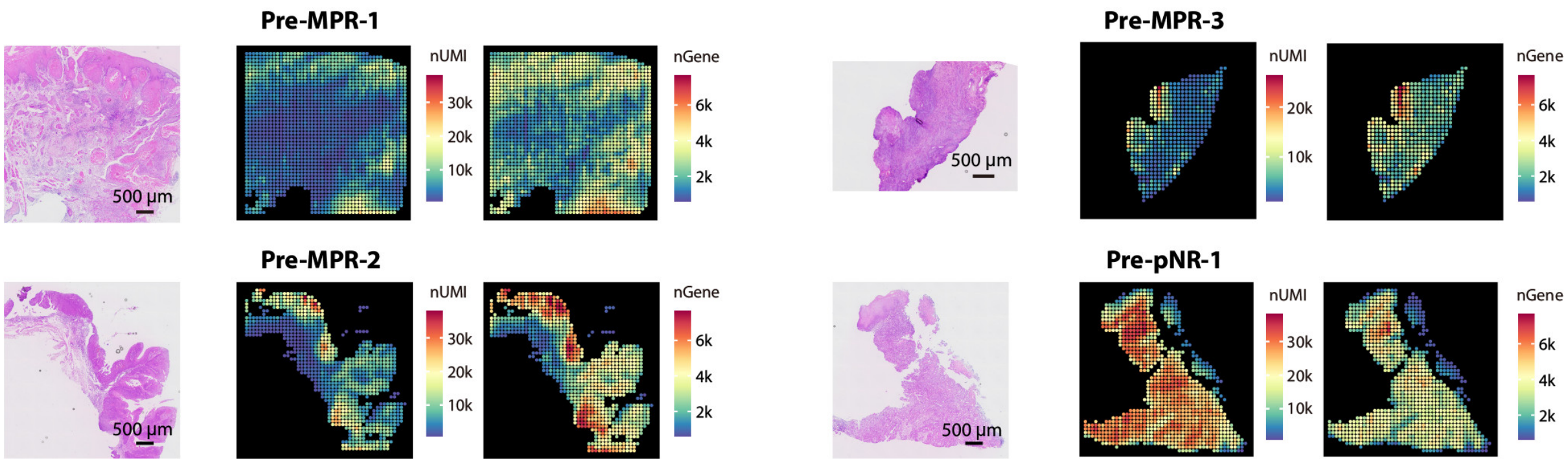

Fig. S21. Assessment of pre-treatment OSCC data quality, related to Fig. 6. Columns 1 and 4, H&E-stained tissue sections; Columns 2 and 5, spatial feature plots showing the nUMI; Columns 3 and 6, spatial feature plots showing the nGene.

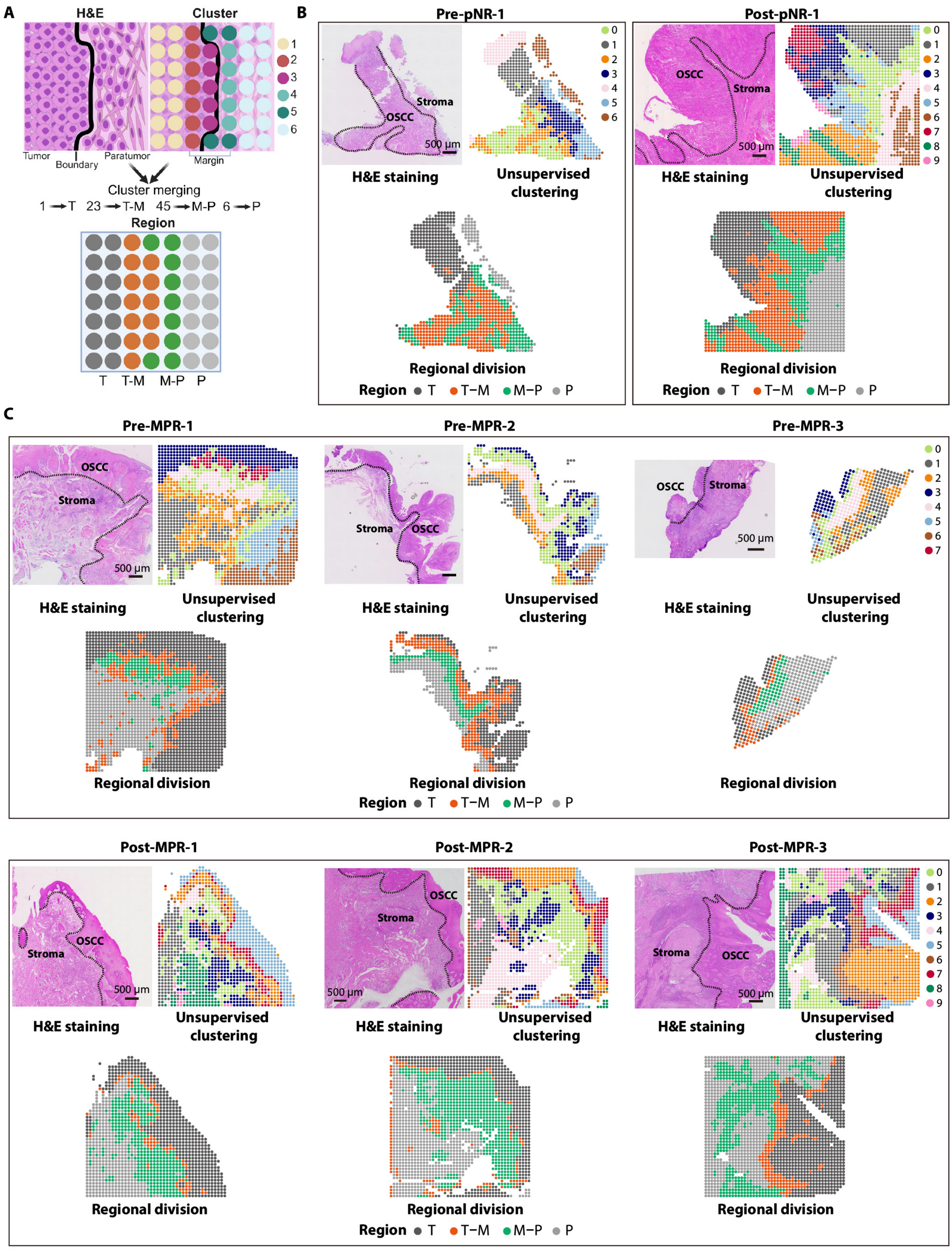

Fig. S22. Identifying different spatial regions in OSCCs before and after chemo-immunotherapy of 50 μm-spot Decoder-FFPE-seq. (A) Schematic diagram of the regional division. Distinct spatial regions independently delineated in each group via unsupervised clustering and tumor border identified H&E annotation. Created in BioRender. jun, Z. (2026) https://BioRender.com/7pai1zj. **(B-C)** Regional division of the pNR-1 sample (**B**) and the MPR group (**C**) before and after chemo-immunotherapy. The H&E

images of the post-treatment pNR-1 sample and MPR group were identical to those in **Fig. 5D**, while the pre-treatment pNR-1 and MPR samples correspond to those in **Fig. S21.**

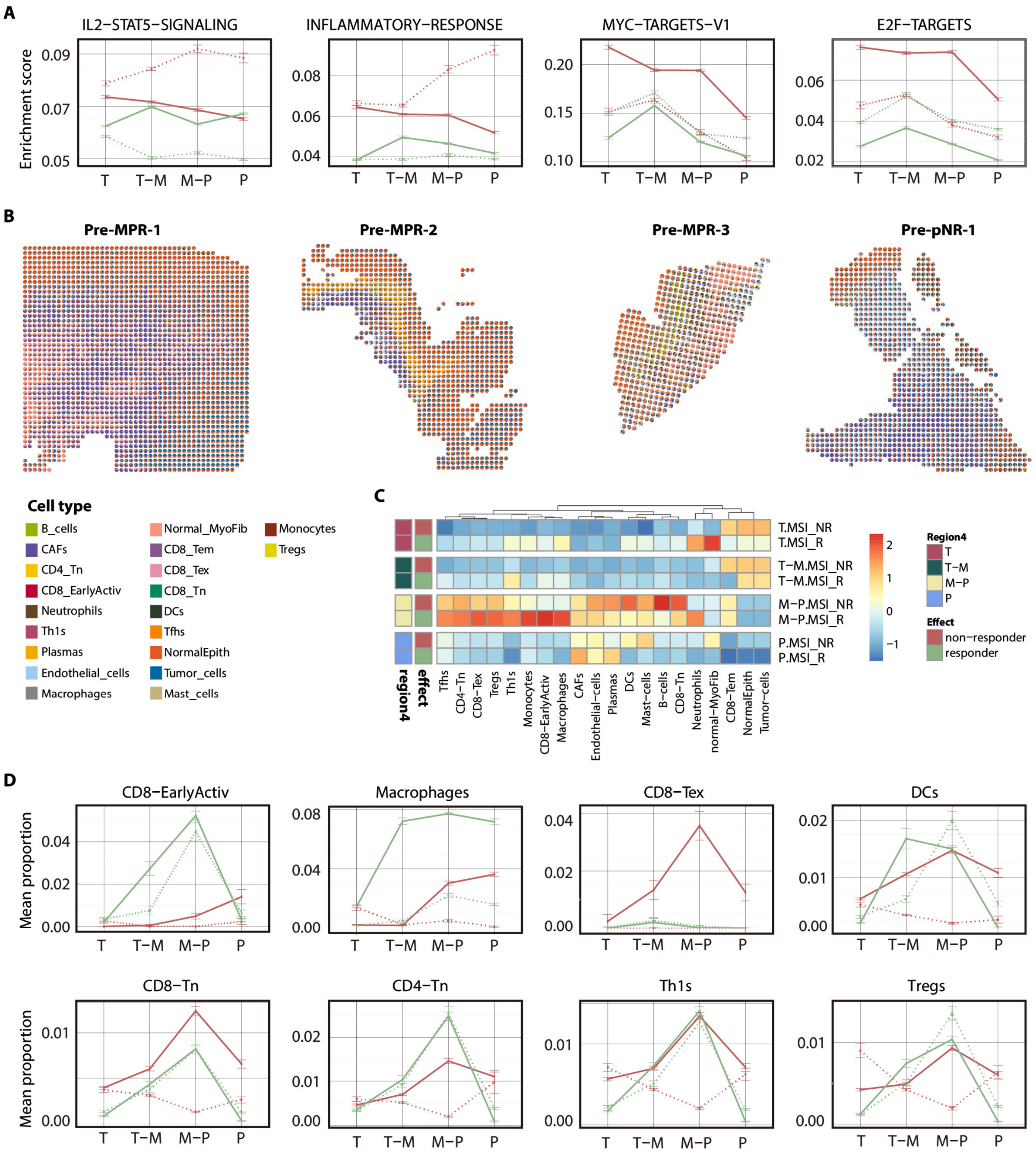

Fig. S23. Decoder-FFPE-seq decodes spatial cellular dynamics in OSCCs before and after chemo-immunotherapy. (A) Hallmark pathway dynamic in different spatial regions before and after treatment among the two groups. **(B)** The spatial pie plot of CARD-inferred cell type composition for four pre-treatment OSCC FFPE specimens. **(C)** Heatmap of cellular abundance across four regions of microsatellite instability (MSI) colorectal cancer before chemo-immunotherapy, based on a previously reported study (*43*). **(D)** Cellular dynamic in different spatial regions before and after treatment among the two groups.

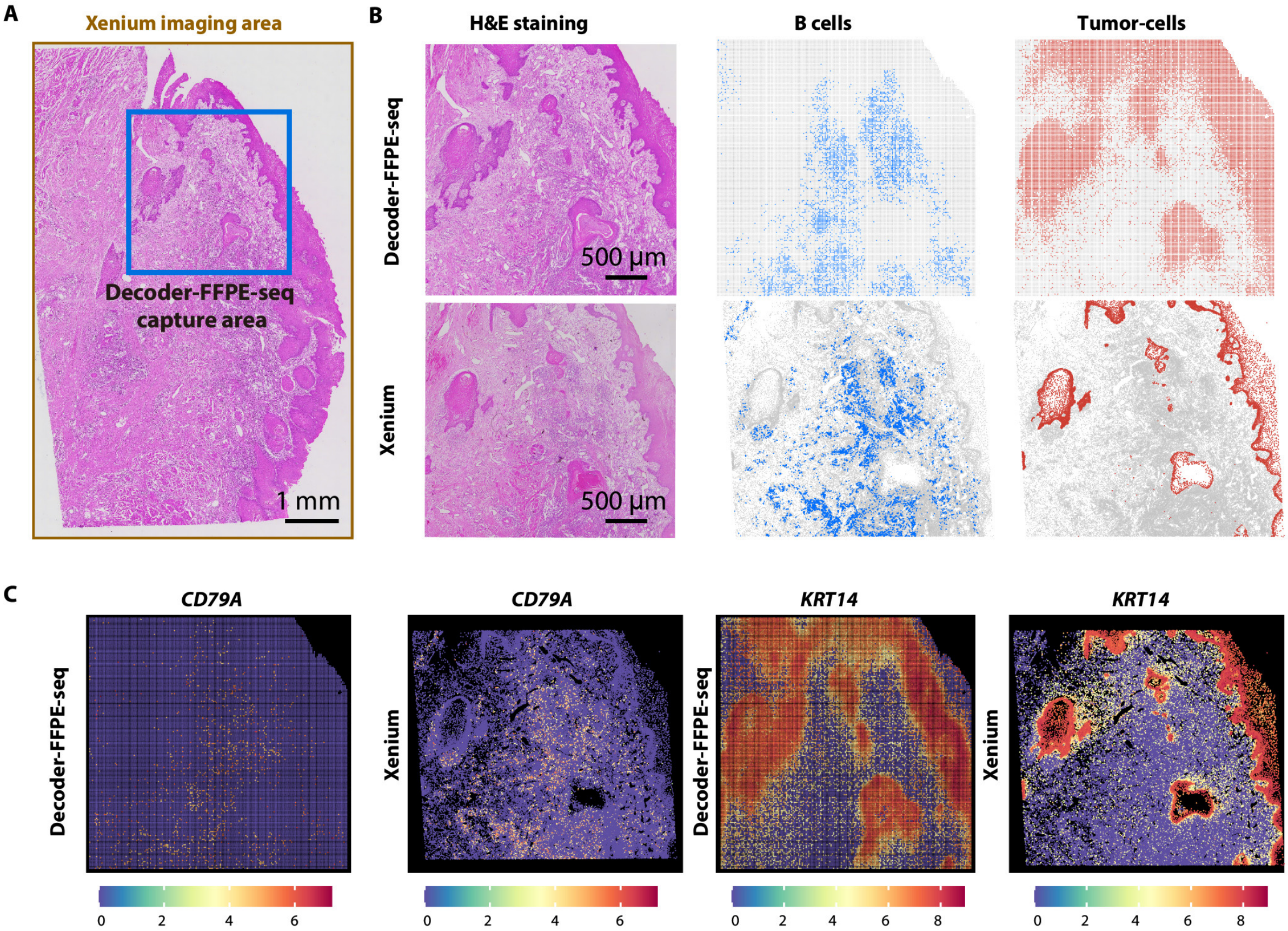

Fig. S24. Comparison of 8 µm-spot Decoder-FFPE-seq and Xenium. (A) Capture regions of Xenium (Brown box) and Decoder-FFPE-seq (blue box). **(B)** Comparison of cell-type distribution between Decoder-FFPE-seq and Xenium. left: H&E images of the corresponding common capture region in Decoder-FFPE-seq and Xenium. Middle: spatial distribution of B cells in Decoder-FFPE-seq and Xenium. Right: spatial distribution of tumor cells in Decoder-FFPE-seq and Xenium. **(C)** Spatial distribution of *CD79A* and *KRT14* in Decoder-FFPE-seq and Xenium.

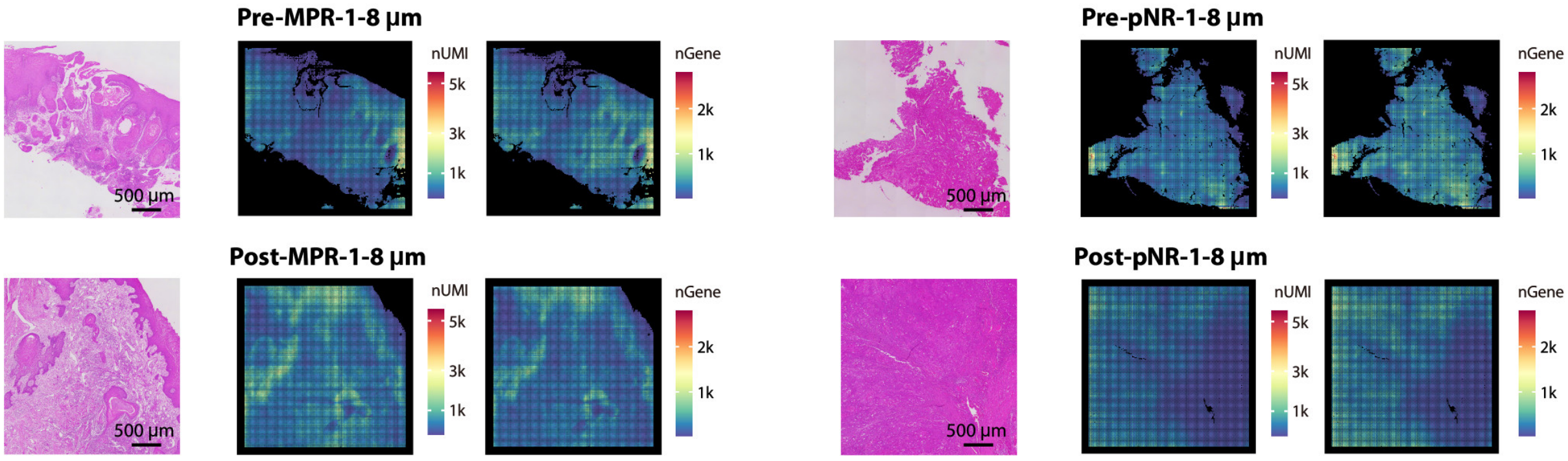

Fig. S25. Assessment of data quality in MPR-1 and pNR-1 samples from 8μm-spot Decoder-FFPE-seq. Columns 1 and 4, H&E-stained tissue sections; Columns 2 and 5, spatial feature plots showing the nUMI; Columns 3 and 6, spatial feature plots showing the nGene. The H&E image of post-MPR-1-8 μm was the same as shown in **fig. S24B.**

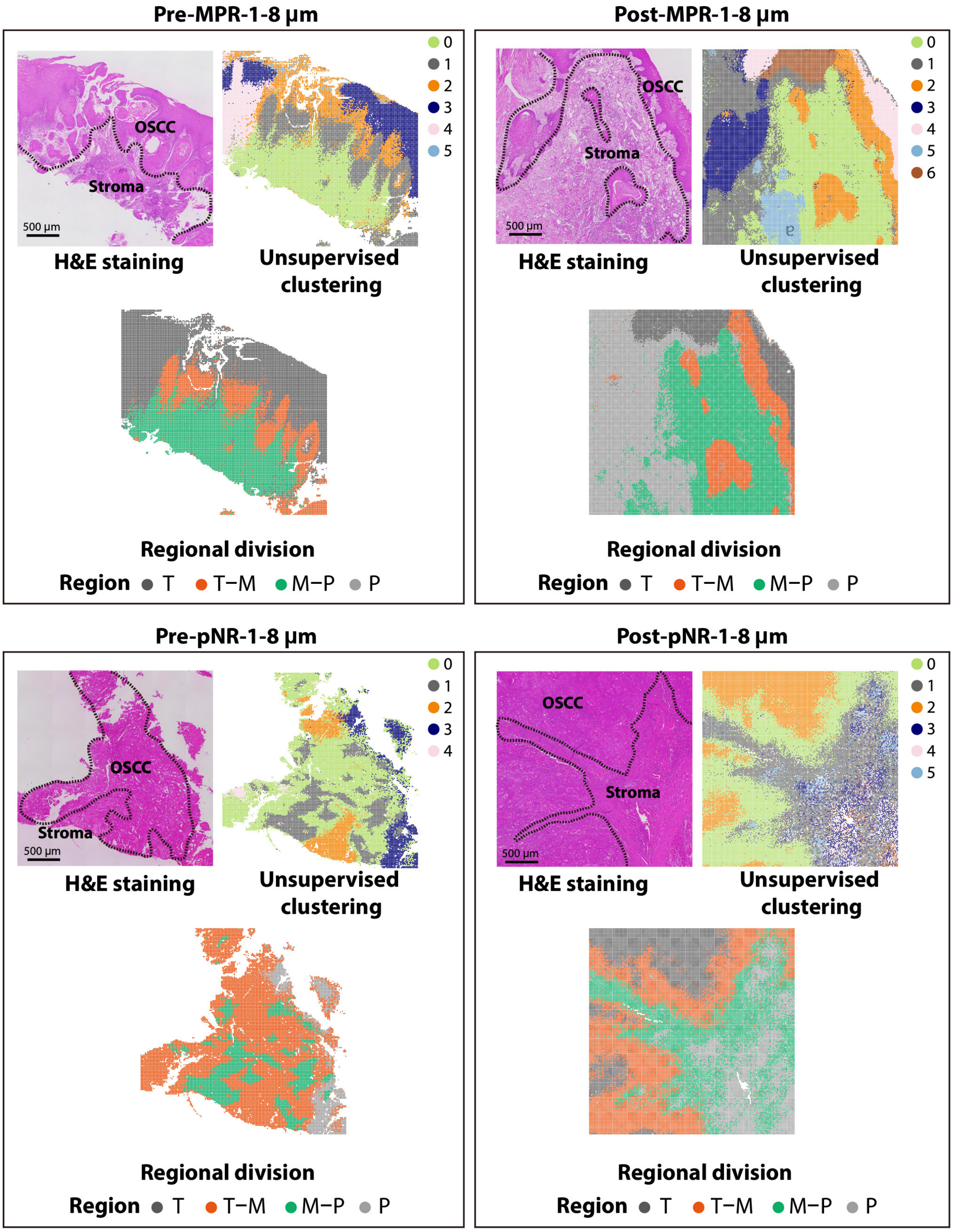

Fig. S26. Identifying different spatial regions in MPR-1 and pNR-1 samples of 8 μm-spot Decoder-FFPE-seq. Their H&E images are the same as those shown in **fig. S25**.

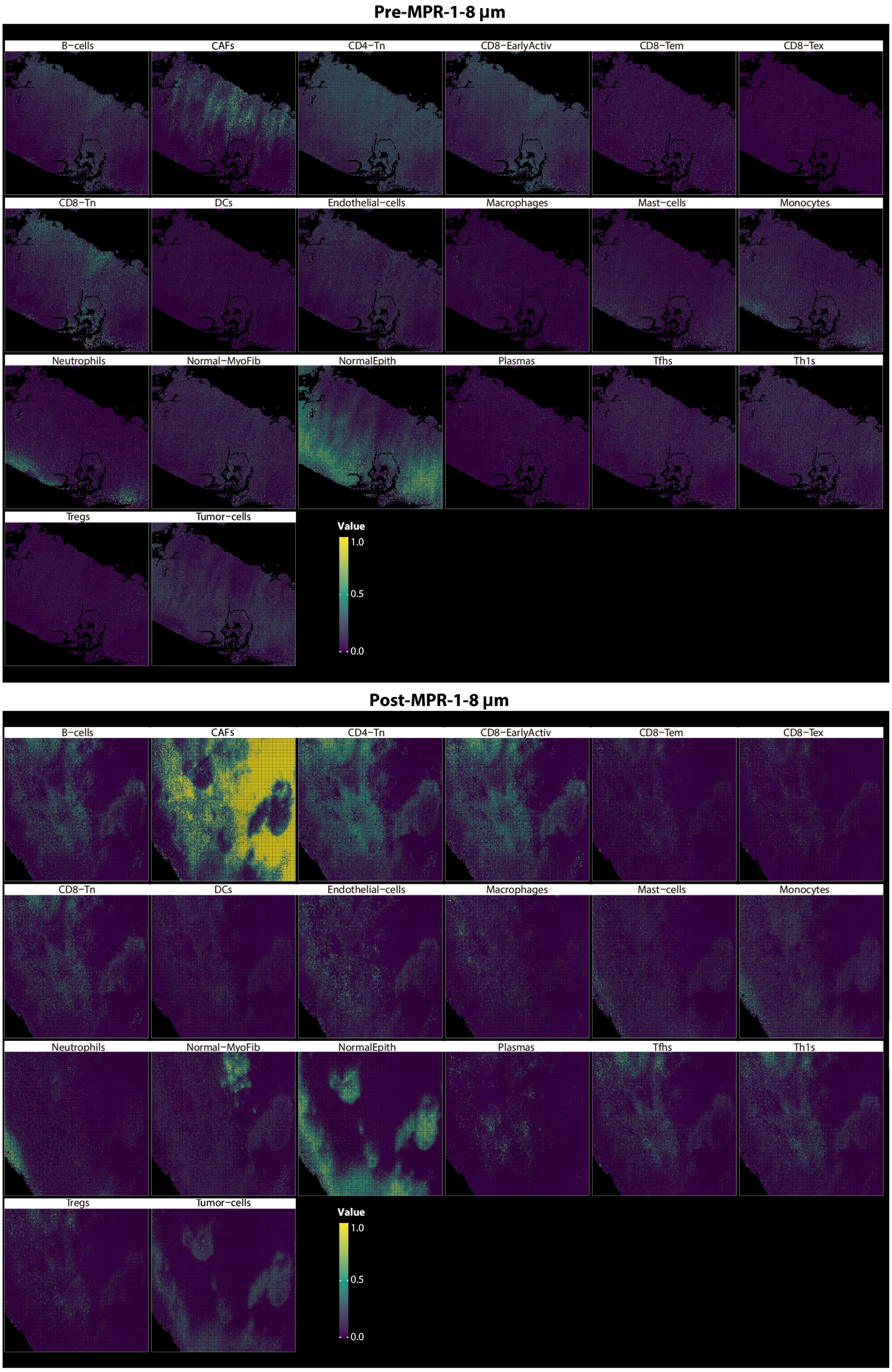

Fig. S27. The spatial distribution of CARD-inferred cell types of pre- and post-treatment MPR-1 from 8 µm-spot Decoder-FFPE-seq.

Pre-pNR-1-8 µm

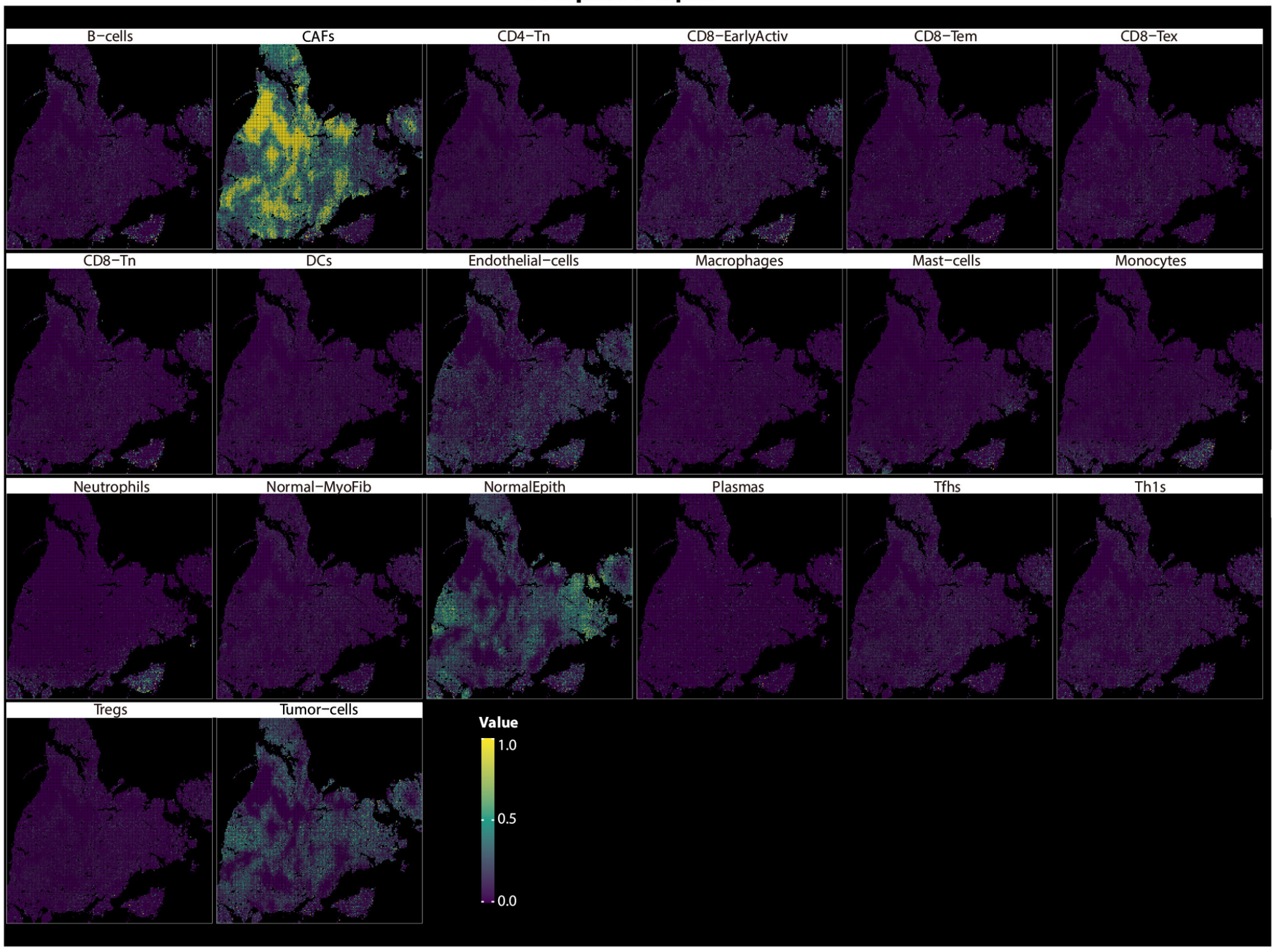

Post-pNR-1-8 µm

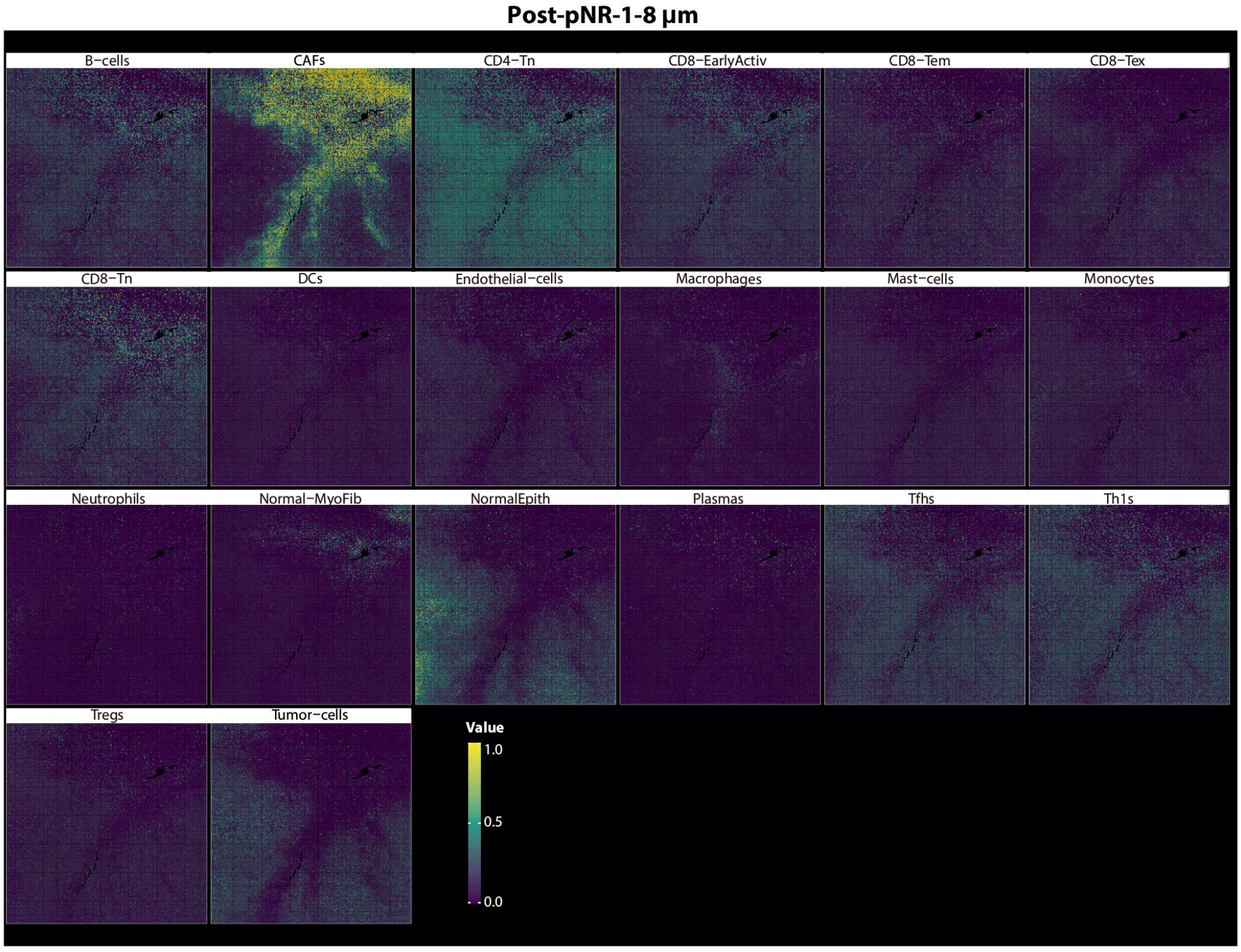

Fig. S28. The spatial distribution of CARD-inferred cell types of pre- and post-treatment pNR-1 from 8 µm-spot Decoder-FFPE-seq.

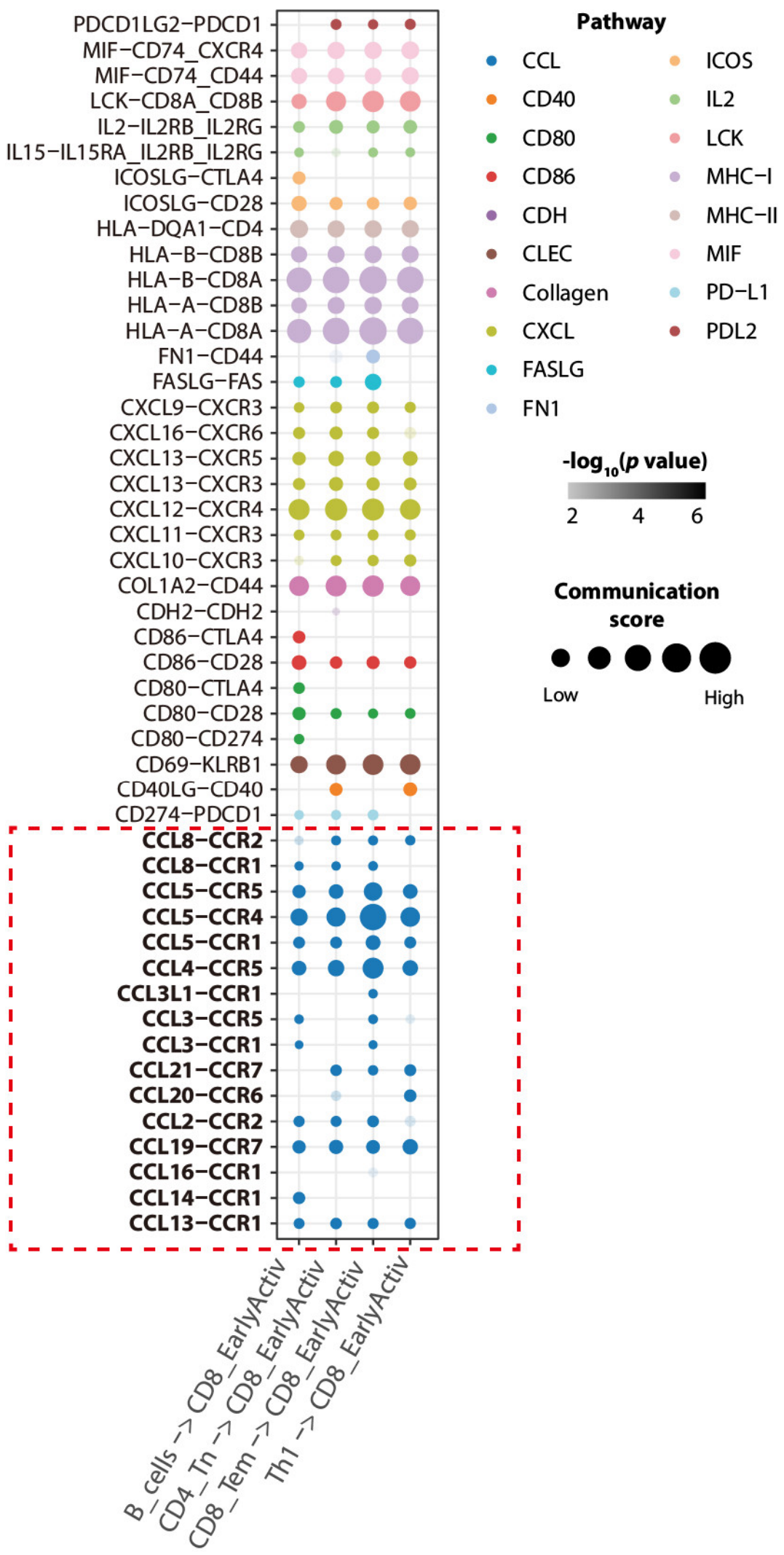

Fig. S29. Cell-cell communication analysis of CD8⁺ EarlyActive with their neighboring cells for post-treatment MPR-1 from Xenium. CCL-related ligand-receptor pairs are highlighted by red boxes.

Table S1. List of a probe panel for targeting the whole mouse transcriptome.

Please see the Sheet of Table S1 in Supplementary Excel Spreadsheet S1.

Sheet of Table S1: List of a probe panel for targeting the whole mouse transcriptome

Table S2. Consumable lists for 8 µm-spot Decoder slides.

Fabrication step	Reagents & consumables	Source	Identifier	Size	Price (USD)	Amount	Cost (USD)/ per slide
Silicon chip fabrication	Silicon chip	Cchip	NA	25 pieces/pack	363.77	1 piece/160 slide	0.09
	Photoresist	Microchem	SU-8 2015	250 mL	698.44	1 mL/8 slides	0.35
	developing solution	Microchem	NA	1L	87.30	5 mL/8 slides	0.05
Glass cleaning & activation	Glass slide	GULUO	NA	50 pieces/pack	58.20	1 piece/2 slide	0.58
	H_2SO_4	Sinopharm	10021618	500 mL	5.38	750 mL/32 slides	0.25
	H_2O_2	Sinopharm	10011218	500 mL	6.11	500 mL/40 slides	0.15
Glass modification	Glycidyloxypropyl trimethoxysilane (GOPTS)	Sigma	440167	100 mL	93.12	20 mL/40 slides	0.47
	Ethanol	Sinopharm	10009259	500 mL	2.84	500 mL/8 slides	0.36
	PAMAM dendrimer, ethylenediamine core, generation 4.0 solution	Sigma	412449	10 g (12mL)	531.83	3.7 mL/16 slides	11.65
	Methanol	Sinopharm	10014118	500 mL	2.40	500 mL/8 slides	0.30
PDMS chip	prepolymer A	Momentive	RTV615	18.16 kg	1321.21	568g/16 slides	2.58
	prepolymer B	Momentive	RTV615	1.82 kg	662.06	56.8g/16 slides	1.29
Cross-linkage of DNA barcode X	DNA barcode X	Tsingke	NA	7680 µL/ 192 barcodes	502.87	4 µL*192 Barcode/16 slides	3.14
	Disuccinimidyl suberate	Thermo	21555	1 g	380.50	40 mg/16 slides	0.95
	DMSO	Sigma	D8418	100 mL	202.69	4.8 mL/8 slides	1.22
	20× SSC	Sangon	B548109	3.5 L	78.43	5 mL/16 slides	0.3
Slide blocking	Succinic anhydride	Aladdin	S104823	500 g	9.17	25.6 g/16 slides	~0.03
	Triethylamine	Macklin	T818774	100 mL	17.17	1.6 mL/16 slides	~0.02
	DMF	Sinopharm	81007718	500 mL	6.91	160 mL/16 slides	0.14
Ligation of DNA barcode Y	DNA barcode Y	Sangon	NA	7680 µL/ 192 barcodes	614.63	4 µL*192 Barcode/16 slides	3.84
	Linker	Sangon	NA	180 µL	1.39	100 µL/16 slides	0.08
	20× SSC	Sangon	B548109	3.5 L	78.43	5 mL/16 slides	0.03
	T4 DNA ligase	Vazyme	C301-01	100 µL	15.28	1920 µL/16 slides	18.33

Total	46.2

Table S3. Comparison of spatially barcoded array-based FFPE-compatible ST platforms.

Method	Array features	Resolution (μm)	Center-to-center spacing (μm)	Barcode-active area fraction	Medium UMIs per spot/bin	Medium UMIs per μm^2	Medium UMIs per bin100 μm
Visium CytAssist_FFPE	Spotted DNAs	55	100	23.8%	13,622	5.8	13,622
Patho-DBiT	Microfluidic barcoded pixels	50	100	25%	19,120	7.6	19,120
Stereo-seq V2	DNA nanoballs	0.22	0.5	15.2%	429<br>10 μm bin	4.3	42,900
Visium HD	*In-situ* synthesized DNAs	2	2	100%	156<br>8 μm bin	2.4	24,375
**Decoder-FFPE-seq**	**Microfluidic barcoded pixels**	**50**	**100**	**25%**	**29,907**	**12.0**	**29,907**
**Decoder-FFPE-seq**	**Microfluidic barcoded pixels**	**8**	**16**	**25%**	**722**	**11.2**	**28,203**

Table S4. List of non-sensory GPCRs identified in Decoder-FFPE-seq data.

Please see the Sheet of Table S4 in Supplementary Excel Spreadsheet S1.
Sheet of Table S4: List of non-sensory GPCRs identified in Decoder-FFPE-seq data

Table S5. List of non-sensory GPCRs identified in Visium HD and Stereo-seq V2 datasets.

Please see the Sheets of Table S5-1 and Table S57-2 in Supplementary Excel Spreadsheet S1.

Sheet of Table S5-1: Non-sensory GPCRs identified in Visium HD data

Sheet of Table S5-2: Non-sensory GPCRs identified in Stereo- seq V2 data

Table S6. Detected the type and UMI counts of non-sensory GPCR in Fig. 2A.

Method	Type	Average nUMI per spot/bin	Average nUMI per um^2
Decoder-FFPE-seq (8 μm)	340	7.03	0.11
Visium HD (8 μm bin)	337	1.53	0.02
Stereo-seq V2 (10 μm bin)	341	5.72	0.06

Table S7. List of probe panels for targeting the whole human transcriptome.

Please see the Sheets of Table S7-1 and Table S7-2 in Supplementary Excel Spreadsheet S1.

Sheet of Table S7-1: List of a human panel with an average of one probe pair

Sheet of Table S7-2: List of a human panel with an average of three probe pairs

Table S8. Clinical information of OSCC samples in Fig. 3 and Fig. 4.

Sample ID	Sample	Sex	Age	Archived time
BC	BC	Female	62	2
LN	LN	Male	56	5
OSCC-6 year	OSCC	Male	66	6
OSCC-8 year	OSCC	Male	49	8
OSCC-12 year	OSCC	Male	62	12
OSCC-15 year	OSCC	Male	62	15
OSCC-20 year	OSCC	Male	58	20
OSCC-20’ year	OSCC	Male	26	20
OSCC-LncRNA	OSCC	Male	43	2
OSCC-HPV	OSCC	Male	68	2
OSCC-8 μm	OSCC	Female	46	2

BC: Breast cancer; LN: Lymph node

Table S9. Cell-cell interactions in cluster3, 6 and 32

Please see the Sheet of Table S9 in Supplementary Excel Spreadsheet S1.

Sheet of Table S9: Cell-cell interactions in cluster3, 6 and 32

Table S10. List of a probe panel for targeting the important IncRNAs of OSCC.

Please see the Sheet of Table S10 in Supplementary Excel Spreadsheet S10.

Sheet of Table S10: List of a probe panel for targeting the important IncRNAs of OSCC

Table S11. IncRNA transcript counts in Fig. 4d.

Genes	Decoder-FFPE-seq	smFISH
MALAT1	1670380 ± 583052	1890899 ± 245421
NEAT1	652970 ± 226104	892563 ± 40968
H19	878602 ± 515584	766192 ± 174213

Table S12. List of a probe panel for targeting HPV16/18-*E6/E7* mRNA in OSCC. Please see the Sheet of Table S12 in Supplementary Excel Spreadsheet S1.

Sheet of Table S12: List of a probe panel for targeting HPV16/18-E6/E7 mRNA in OSCC

Table S13. Clinical characteristics and pathological response of OSCC patients.

Patient ID	Sample ID	Sex	Age	Site	Stage	Grade	Treatment	Pathological response
P02	MPR-1	Male	56	tongue	T3N0M0	III	Camrelizumab+TPF	MPR
P07	MPR-2	Male	52	floor	T4aN2bM0	IVA	Camrelizumab+TPF	MPR
P43	MPR-3	Male	38	tongue	T3N1M0	III	Camrelizumab+TPF	MPR
P13	pPR-1	Male	37	tongue	T3N0M0	III	Camrelizumab	pPR
P45	pPR-2	Male	32	tongue	T3N0M0	III	Camrelizumab	pPR
P56	pNR-1	Male	50	buccal	T3N1M0	III	Camrelizumab+TPF	pNR
P49	pNR-2	Male	61	buccal	T2N2bM0	IVA	Camrelizumab	pNR

Abbreviation: TPF, docetaxel-cisplatin-5-fluorouracil; TP, docetaxel-cisplatin; pMPR, major pathological response; pPR, partial pathological response; pNR, pathological non-response

Table S14. List of 430-gene panel for the Xenium in situ high-plex assay in OSCC.

Please see the Sheet of Table S14 in Supplementary Excel Spreadsheet S1.

Sheet of Table S14: List of 430-gene panel for the Xenium in situ high-plex assay in OSCC

Table S15. The probe panel information for representative samples.

Sample	Targeted RNA types	Number of probe pairs	Number of RNA targets	Total genes detected	Reads mapped to probe panel	Reads mapped confidently to probe panel
MOB	mRNA	20,057	19,758	19,297	92.27%	90.95%
Mouse brain-50 μm	mRNA	20,057	19,758	19,293	91.81%	91.07%
Mouse brain-8 μm	mRNA	20,057	19,758	19,227	91.29%	90.16%
Human lymph node	mRNA	19,008	18,308	17,831	79.28%	78.17%
Human lymph node	mRNA	53,504	18,085	18,040	98.50%	97.78%
OSCC-8 μm	mRNA	53,504	18,085	18,004	99.10%	98.47%
OSCC-15 year	mRNA	53,504	18,085	18,036	98.71%	91.60%
OSCC-IncRNA	InRNA	2,177	275	275	98.56%	97.88%
OCSS-mRNA+IncRNA	mRNA + IncRNA	21,185 (19008+2177)	18,360 (18085+275)	18,039	81.76	80.99%
OSCC-mRNA+HPV	mRNA + HPV RNA	19,224 (19008+16)	18,087 (18085+2)	17,780	73.40%	72.48%
Post-pNR-1-50 μm	mRNA	19,008	18,308	17,734	79.38%	78.04
Post-pNR-1-8 μm	mRNA	53,504	18,085	17,989	98.57%	97.23%

Table S16. Study reagents for Decoder-FFPE-seq.

Reagent or resource	Source	Identifier
Xylene	Shanghai Lingfeng	1330-20-7
Ethanol	Shanghai Lingfeng	64-17-5
Hematoxylin, Mayer’s	Sigma-Aldrich	51275
Bluing Buffer	Dako	CS702

Alcoholic Eosin Y	Abcam	ab246824b
Glycerol	Sigma-Aldrich	G5516
0.1 M HCl	TMRM	BW2014-0.1
Sodium citrate	Aladdin	S189183
EDTA	Thermo Fisher Scientific	AM9260G
Sodium Dodecyl Sulfate (SDS)	Thermo Fisher Scientific	AM9820
Tween 20	Sigma-Aldrich	P9416
10× PBS	Thermo Fisher Scientific	70011044
Formamide	Thermo Fisher Scientific	AM9342
Yeast tRNA	Thermo Fisher Scientific	AM7119
DNase/RNase-Free Distilled Water	Thermo Fisher Scientific	10977023
20×Saline Sodium Citrate (SSC) Buffer	Thermo Fisher Scientific	AM9770
SplintR Ligase	New England Biolabs	M0375
RNase H	New England Biolabs	M0297
RNase If	New England Biolabs	M0243
Proteinase K	QIAGEN	19131
Buffer PKD	QIAGEN	1034963
PEG 8000	PERFEMIKER	PMA020380
Klenow fragments (3′–5′ exo-)	New England Biolabs	M0212
dNTP mix	Thermo Fisher Scientific	R0192
KOH	Sigma-Aldrich	P4494

Tris-HCl	Songon	B548137
Kapa Hotstart HiFi ReadyMix	Roche	KK2602
Kapa qPCR ReadyMix	Roche	KK4601
Ampure XP Beads	Vazyme	N411-03
Equalbit 1 × dsDNA HS Assay kit	Vazyme	EQ121-01
RNeasy FFPE Kit	QIAGEN	73504
Primers, Ligation linkers, DNA barcodes	Songon	table S17

Table S17. DNA sequences involved in Decoder-FFPE-seq workflow.

Name	Sequence
DNA barcode X	5' NH2-C6-CTACACGACGCTCTTCCGATCT [barcode X] AGGCCAGAGCATTCG 3'
DNA barcode Y	5' Phos- ATCCACGTGCTTGAG [barcode Y] NNNNNNNNNNNNTTTTTTTTTTTTTTTTTTTTTTTTTTTTTTVN
Ligation linker	CTCAAGCACGTGGATCGAATGCTCTGGCCT
Forward primer	CTACACGACGCTCTTCCGATCT
Reverse primer	GTGACTGGAGTTCCTTGGCACC
i5 primer	AATGATACGGCGACCACCGAGATCTACAC[i5] ACACTCTTTCCCTACACGACGCTC
i7 primer	CAAGCAGAAGACGGCATACGAGAT[i7] GTGACTGGAGTTCCTTGGCACCCGAGAATTCCA

Supplementary methods

Design of probe libraries

We designed IncRNA probes with three main steps: probe generation, probe filtering, and probe set selection for each gene. Specifically, probe RNA sequences for each target gene were first extracted from the genomic sequences, and candidate probe sequences of 50 nucleotides (k-mers) were generated with calculated melting temperature (Tm) and GC content. Second, probe filtering was conducted by sequence property and binding specificity: removal of probes that 1) contain runs of five or more identical nucleotides, 2) have a GC content or Tm outside a user-specified range (58 °C≤Tm≤80 °C; 40%≤GC content≤60%), 3) are predicted to form stable secondary structures or self-dimers, specifically, probes with a minimum free energy (*ΔG*) of secondary structure below -20 kcal/mol at 37 °C, or with self-complementary regions longer than 20 nucleotides, were discarded, and 4) potentially bind to non-target genes using BLASTn alignment search with ≤ 5 mismatched bases. Finally, the best set of non-overlapping probes is identified for each gene by grouping them into non-overlapping subsets using a graph-based maximal subset algorithm and calculating scores. The optimal design parameters were set as follows: target Tm = 66.92 °C, GC content = 50%. Non-overlapping probes and probe set were scored by the distance to the optimal GC content and Tm with Tm_weight of 1, GC_weight of 1, and isoform_weight of 0.5. The top ten non-overlapping probes with the highest scores were selected as the final probes for each gene.

Comparison of Decoder-FFPE-seq with similar ST platforms

In addition to comparing the detected genes and UMIs, we conducted saturation curve analysis in order to assess gene detection sensitivity at consistent sequencing depths. This was achieved through systematic down-sampling of sequence reads from FASTQ files, followed by realignment using our standard preprocessing pipeline to generate comparable gene counts matrices across varying sequencing depths. And the saturation was calculated by

$$saturation = 1 - \frac{n_{sampled\ reads}}{n_{original\ reads}},$$

where $n_{sampled\ reads}$ represents the UMI counts in the generated gene counts matrix , and $n_{original\ reads}$ enumerates the total count of sequencing reads that meet stringent quality criteria, including both confidently mapped validated barcode and UMI tags.

Decoder-FFPE-seq protocol

RNA quality evaluation

TIMING ~1.5h

1. About four 10-µm-thick tissue sections were sliced for RNA extraction using RNeasy FFPE Kit.
2. Extracted total RNA was then measured using the Agilent Bioanalyzer (Agilent, TapeStation 4150), and the proportion of RNA fragments larger than 200 nucleotides was obtained (DV200).

Tissue preparation

TIMING ~5h

1. The paraffin blocks were sliced into 5-µm sections and mounted onto the center of adhesive slides;
2. Tissue sections were subjected to 42°C baking for 3 hours to ensure complete drying;

▲ **NOTE：** Stored slides at -80°C for up to 1 month until use.

3. Tissue sections were subjected to 60°C baking for 2 hours to promote adhesion to glass slides;
4. Deparaffinization and rehydration were performed using a standardized process: xylene I (10 min), xylene II (10 min), anhydrous ethanol I (3 min), anhydrous ethanol II (3 min), 95% ethanol I (3 min), 95% ethanol II (3 min), 85% ethanol (3 min), 70% ethanol (3 min), and water (30 s).

Imaging

TIMING ~0.5h

5. The deparaffinized and rehydrated sections were stained with hematoxylin (3 min), rinsed with deionized water (15 dips), incubated with bluing buffer (2 min), rinsed deionized water (5 dips), and stained with alcoholic eosin (1 min) followed by deionized water rinse (20 dips).

▲ **NOTE：** After eosin washing, immediately mount sections in glycerol to prevent tissue dehydration-induced darkening during imaging.

6. Tissue sections were mounted with 85% glycerol and coverslipped for bright field imaging (e.g. BX43F, Olympus in our study). Following imaging, coverslips were removed by water immersion, and sections were dried at 37°C for 5 min.

■ **PAUSE POINT:** Store slides at 4°C up to 2 weeks.

Decrosslinking and permeabilization of tissue sections

TIMING 1.5h

7. For destaining, the tissue sections were incubated with 0.1 M HCl at 42°C for 15 min.
8. Tissue sections were incubated decrosslinking reagents, which were optimized to be 10 mM sodium citrate (pH = 6.5) with 0.05% Tween-20, at 95°C for 1 hour, followed by 10-min cooling to room temperature.
9. The tissue sections were permeabilized with 0.1% Tween-20 in PBS at room temperature for 15 min.

Hybridization and ligation of probes

TIMING 16h-24h

10. Each tissue section was incubated with 100 μL hybridization mix at 50°C for 16-24 hours. The mix contained 30 μL 20× SSC, 10 μL 100% formamide, 10 μL 10 mg/mL yeast tRNA, 20 μL probes (10 μL right probes + 10 μL left probes, 2.5 nM each probe), and 30 μL water.
11. Following hybridization, tissue sections were washed thrice (5 min/wash, 50°C) with 200 μL of wash buffer (2× SSC, 10% formamide).
12. Probe ligation was performed at 37 °C for 1 hour using a ligation mix consisting of 6 μL 10× ligation buffer, 3 μL SplintR Ligase (25 U/μL), 15 μL 100% glycerol and 36 μL water.
13. After probe ligation, tissue sections were washed three times (5 min/wash, 57°C) with 200 μL wash buffer.

▲ **Note:** To maintain tissue temperature, keep aspiration time to a minimum during post-hybridization and post-ligation washes.

■ **PAUSE POINT:** Store slides in 2× SSC at 4°C up to 1 day.

Probe-blotting

TIMING ~1h

Option 1: Manual probe-blotting protocol

14. Paste a 10-μm-thick film spacer around the barcoded DNA array on the Decoder slide;
15. Find a colored box to hold Decoder slides, which facilitate visual inspection of the transparent film with the naked eye;

16. Prepare 25 μL of probe-blotting mix, which was optimized as 2.5 μL of 10× RNase H buffer, 1.25 μL RNase H (25 U/μL), 1.25 μL RNase If (50 U/μL), 1.55 μL Diluted Proteinase K (8-fold diluted, 155 mg/ml) and 18.45 μL water;
17. Add probe-blotting mix onto the array;
18. Align the HE-stained tissue slides with the Decoder slides carefully

◆ **CRITICAL STEP:** Carefully align the two slide, avoid air bubbles, and do not readjust once aligned. Uneven contact, bubbles, or repositioning can compromise sequencing quality, causing UMI dropouts and increased RNA diffusion.

19. Pick up the small box (with two slides) and place it in a 37 °C oven to react for 30 minutes;

▲ **Note：** Ensure that the two glass slides remain aligned and do not shift.

20. After probe-blotting, remove the tissue slide carefully and then wash the Decoder slide three times with 2× SSC ;

▲ **Note：** After reaction, the fluid should still be present between two slides.

◆ **CRITICAL STEP:** Be extremely careful when removing tissue slides, avoiding excessive friction, as this will affect the final sequencing result.

Option 2: Probe-blotting protocol with instrument

21. To achieve probe blotting for spatial barcoding, the tissue section was stained using diluted eosin (10% alcoholic eosin in PBS) again for 1 min at room temperature. This enables precise alignment of each tissue section with the barcoded array on the Decoder slides using a home-made probe-blotting instrument.
22. After washing with PBS three times and slightly dried, the tissue slide was loaded on the lid of home-made probe-blotting instruments immediately, while the slide with dendrimeric DNA coordinate barcoding arrays was fastened to the base.
23. The tissue was maneuvered to align the selected regions precisely with the barcoded DNA array.
24. After alignment, 25 μL of probe-blotting mix, which was optimized as 2.5 μL of 10× RNase H buffer, 1.25 μL RNase H (25 U/μL), 1.25 μL RNase If (50 U/μL), 1.55 μL Diluted Proteinase K (8-fold diluted, 155 mg/ml) and 18.45 μL water, was added onto the array. The lid was closed and incubated for 30 min at 37°C.

◆ **Critical step:** Proteinase K was added immediately before probe-blotting operation, and the entire procedure was completed within 3 min post-addition.

25. Bright-field imaging was performed to document coordinates of the transferred tissue for downstream registration analysis.

Probe extension

TIMING 1h

26. After probe-blotting, the Decoder slide was washed three times with 2× SSC buffer.

◆ **CRITICAL STEP:** Slides must be washed immediately after blotting to prevent ectopic binding of residual probes.

27. The captured probes were extended by adding 75 μL extension mix and incubating at 37°C for 1 hour. The extension mix contained 7.5 μL 10X NEBuffer™, 7.5 μL 10 mM dNTP, 7.5 μL Klenow fragments (3′–5′ exo-) (5 U/μL), 18 μL 50% PEG8000, and 34.5 μL water.

28. After probe extension, the slide was washed with 2× SSC buffer three times. The resulting cDNA was eluted from the arrays by adding 35 μL KOH (80 mM, 10 min) and neutralized using 5 μL 1 M Tris (pH = 7.0).

■ **PAUSE POINT:** Store samples at −20°C up to 1 month.

Library construction and sequencing

TIMING ~4h

29. The cDNA (40 μL) was first pre-amplified with 50 μL 2× KAPA HiFi Hotstart ReadyMix and 10 μL cDNA primers (8 μM, reverse primer + forward primer) using the following PCR program: 98 °C for 3 min; 5 cycles of 98 °C for 20 s, 63 °C for 20 s and 72 °C for 1 min; 72 °C for 5 min, and 4°C hold. PCR products were then purified with DNA beads at a bead-to-sample ratio of 1.8×.

■ **PAUSE POINT:** Store samples at −20°C up to 1 month.

30. Quantitative PCR (qPCR) mix (10 μL) was prepared with 1 μl of purified products, 1× Kapa qPCR ReadyMix, 0.5 μM cDNA primer and water. The mix was immediately loaded into a qPCR instrument (LightCycler480 II, Roche Diagnostics) for operation with the following protocol: 98 °C for 3 min, 30 cycles at 98 °C for 5 s and 63 °C for 30 s.

31. The purified PCR products were subjected to a second round of amplification: 98 °C for 3 min; n cycles of 98 °C for 20 s, 67 °C for 30 s and 72 °C for 1 min; and 72 °C for 5 min, and 4°C hold. Final products were purified using 1.8× DNA beads and quantified with a Qubit dsDNA assay kit.

■ **PAUSE POINT:** Store samples at −20°C up to 1 month.

32. 10 ng cDNA products were input for sequencing library construction, which was amplified with 25 μL 2× KAPA HiFi Hotstart ReadyMix, 5 μL P5 Primer (4 μM), and 5 μL P7 Primer (4 μM).

33. Library construction with the following PCR program: 98 °C for 3 min; 8 cycles of 98 °C for 20 s, 60 °C for 20 s and 72 °C for 1 min; 72 °C for 5 min, and 4°C hold.

34. The resulting libraries were purified with 1.2× DNA beads and sequenced using an Illumina NovaSeq 6000 or MGI DNBSEQ-T7 sequencer with a paired-end 150 bp read length.

Supplementary Excel Spreadsheet S1. Compiled Supplementary Tables S1, S4, S5, S7, S9, S10, S12, S14.

Source Data S1. Source data for all figures in main article content and supplementary materials.
